# Japanese wolves are most closely related to dogs and share DNA with East Eurasian dogs

**DOI:** 10.1038/s41467-024-46124-y

**Published:** 2024-02-23

**Authors:** Jun Gojobori, Nami Arakawa, Xiayire Xiaokaiti, Yuki Matsumoto, Shuichi Matsumura, Hitomi Hongo, Naotaka Ishiguro, Yohey Terai

**Affiliations:** 1 SOKENDAI (The Graduate University for Advanced Studies), Research Center for Integrative Evolutionary Science, Shonan Village, Hayama, Kanagawa 240-0193 Japan; 2 Research and Development Section, Anicom Specialty Medical Institute, Naka-ku, Chojamachi, Yokohama, 231-0033 Japan; 3 Faculty of Applied Biological Sciences, Gifu University, Yanagido 1-1, Gifu, 501-1193 Japan

**Keywords:** Phylogenetics, Archaeology, Speciation, Palaeontology

## Abstract

Although the domestic dog’s origin is still unclear, this lineage is believed to have been domesticated from an extinct population of gray wolves, which is expected to be more closely related to dogs than to other populations of gray wolves. Here, we sequence the whole genomes of nine Japanese wolves (7.5–100x: Edo to Meiji periods) and 11 modern Japanese dogs and analyze them together with those from other populations of dogs and wolves. A phylogenomic tree shows that, among the gray wolves, Japanese wolves are closest to the dog, suggesting that the ancestor of dogs is closely related to the ancestor of the Japanese wolf. Based on phylogenetic and geographic relationships, the dog lineage has most likely originated in East Asia, where it diverged from a common ancestor with the Japanese wolf. Since East Eurasian dogs possess Japanese wolf ancestry, we estimate an introgression event from the ancestor of the Japanese wolf to the ancestor of the East Eurasian dog that occurred before the dog’s arrival in the Japanese archipelago.

## Abstract (web summary)

The evolutionary origin of the domestic dog is uncertain. Here, the authors sequence the whole genomes of 9 extinct Japanese wolves and 11 modern Japanese dogs, applying a phylogenetic analysis to show that dogs may have originated in East Asia from a common ancestor with the Japanese wolf.

## Introduction

The ancestor of the domestic dog is the gray wolf (*Canis lupus*). Extant gray wolves are divided into three groups: the North American, Eurasian, and domestic dog groups^1^. Recent phylogenomic analyses of gray wolves have shown that the North American gray wolf diverged at the basal ancestral position, followed by the Eurasian lineage^1,2^. Dogs form a monophyletic clade which is the sister group to the Eurasian lineage of the gray wolf ^1–3^. Therefore, the hypothesis that the dog lineages originated in Eurasia has been widely accepted. But there is still much debate concerning when, where, how many times, and from which population, the ancestor(s) of dogs was domesticated^2–10^. Because no extant population of gray wolves has been reported to be more closely related to dogs than the other wolf populations, it is believed that the dog lineage has been domesticated from an extinct population of gray wolves^1,3,5,9,11–13^. However, to our knowledge, no information has been available about this extinct population.

Many regions in Eurasia, including southern East Asia^4,8,13–15^, Central Asia^16^, Europe^9^, Siberia^17^, and both West and East Eurasia (dual origin)^12,13^, have been proposed as candidates for the origin of dogs. However, the origin of dog domestication is still under debate because the temporal origin of dog domestication, thus when the dog began to associate with humans, cannot be equated with the genetic split time between populations of wolves and dogs. Divergence between the Eurasian gray wolf and dog lineages has been estimated to be 20,000-40,000 years ago^11,18^. Based on phylogenomic analyses, dogs were initially reported to be genetically divided into two distinct lineages, i.e., the West and East Eurasian lineages^2,3,12,16–19^. A subsequent report suggested an ancient divergence of the Arctic sled dog lineage^17,20^, which is closely related to the pre-contact American dogs^2^. The West Eurasian and East Eurasian lineages diverged 17,000–24,000 years ago^18^, and a sled dog ancestry was present in the genome of a 9500-year-old Siberian dog^20^.

Studies have suggested that wolf populations have undergone introgression^9,10,14,16,20,21^ with dogs or bidirectional gene flow between African dogs and an Israeli wolf^3^. Moreover, recent ancient genome analyses have shown that dogs are overall closer to eastern Eurasian wolves and the wolf genome related to the modern southwestern Eurasian population has introgressed into the ancestry of early Near Eastern and African dogs^13^.

The Japanese wolf (*Canis lupus hodophilax* Temminck, 1839) was a subspecies of the gray wolf that inhabited Honshu, Shikoku, and Kyushu Islands in the Japanese Archipelago and became extinct 100–120 years ago^22^. Recently, the genome of a 19th century “Honshu wolf” (one of the common names for the Japanese wolf) specimen from the collection of the British Museum was sequenced to an average depth of coverage of 3.7x. The analysis of DNA from this specimen suggests that this individual is closely related to a lineage of Siberian wolves that existed in the Late Pleistocene and shows significant gene flow with Japanese dogs^23^. A subsequent report suggested a hypothesis that Pleistocene Eurasian wolves and a wolf close to the modern Eurasian wolves migrated to the Japanese archipelago and became the ancestors of the Japanese wolf^24^.

In this study, we sequence nine genomes of Japanese wolves and 11 genomes of modern Japanese dogs at high coverage and analyze these together with one hundred dog and wolf genomes from public databases. The analyses show that (1) the Japanese wolf was a unique subspecies of the gray wolf that is genetically distinct from both extant and ancient gray wolves known to date, (2) the Japanese wolf is most closely related to the monophyletic group of dogs. Furthermore, (3) Japanese wolf ancestry has introgressed into the ancestor of East Eurasian dogs at an early stage of the dog’s history after diverging from the West Eurasian lineages. The genome derived from Japanese wolf ancestry has been inherited by many modern dogs (at most 5.5%), even in the West Eurasian lineages, through admixture with East Eurasian lineages.

## Results

### Relationships between Japanese wolves and other dogs and gray wolves

For the present study, we assigned nine individuals (from Edo and Meiji periods, see Supplementary Data 1) with Japanese wolf-type mitochondrial DNA haplotypes^25^ as the Japanese wolf. There are 11 indigenous breeds of dogs in Japan^26^, and three breeds (Akita, Kishu, and Shiba) were used as Japanese dogs in this study. Genomic DNA sequences of these nine Japanese wolves (18.5–241 Gb: average depth of coverage, 7.5-100x) and 11 Japanese dog individuals (59-127 Gb: average depth of coverage, 24-53x) were determined (Supplementary Data 1, Supplementary Fig. 1). In addition, we used sequence data of 56 dogs and 23 modern gray wolves with a depth of coverage >20x, four ancient dogs and eight ancient canids with a depth of coverage >5x, and six outgroup species from the public database (Supplementary Data 2, Supplementary Figs. 2 and 3).

**Fig. 1:**
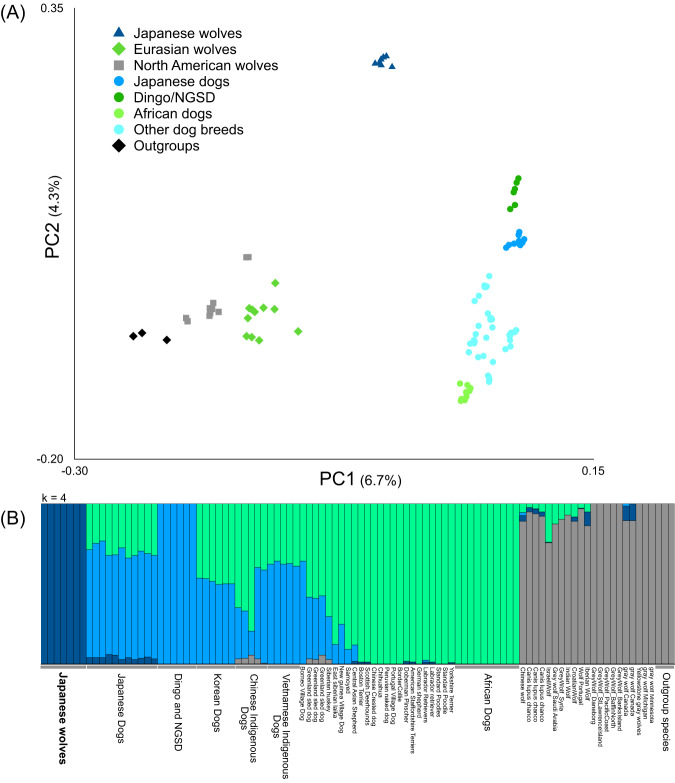
Relationships between Japanese wolves and other canids. **A** Principal Components Analysis (PC1 versus PC2) of 98 samples based on 290,414 unlinked biallelic SNPs extracted from 1,696,115 sites (see Supplementary Data 2 for sample information). Colored circle, square, and triangle correspond to the names of dog or wolf in the panel. **B** ADMIXTURE results based on SNP data for K = 4 (see Supplementary Data 2 for sample information). Outgroup species are Coyote, African Golden Wolf, and Golden Jackal. Source data are provided as a Source Data file.

All sequence data were mapped to the reference genome sequence (CanFam3.1). After haplotype calling and gvcf file merging, single nucleotide polymorphisms (SNPs) were genotyped. We created fifteen datasets to maximize the number of SNP sites for each analysis (see Methods). To examine the genetic relationship among the individuals used in this study, a principal component analysis (PCA) was performed using individuals with high coverage (Fig. 1A).

In the PCA, Japanese wolves formed a distinct cluster, suggesting that Japanese wolves were genetically separated from dogs, gray wolves, and any of the outgroup species. The PC1 axis separates the clusters of the gray wolves and the dogs. The dogs formed a cluster spread along the PC2 axis. Dingoes and New Guinea singing dogs (NGSD) were the closest to Japanese wolves among dogs along the PC2 axis, followed by a cluster of Japanese dogs (Fig. 1A). Note that the positions in PCA plot indicates the genetic characteristics of the populations but do not indicate phylogenetic relationships and a past introgression event. Using the same data set we also generated an ADMIXTURE result with the lowest CV error at *K* = 4 (Fig. 1B). In this analysis, Japanese wolves also formed clusters with higher K such as *K* = 5 or *K* = 6, indicating that their genetic composition was unique compared with that of the other dogs and gray wolves (Fig. 1B and Supplementary Figs. 4 and 5).

Next, we added the three Japanese wolf individuals with low coverage [a “Honshu wolf”^23^: average coverage 3.7x] and with a low proportion of the reference genome covered (Leiden b^27^: 12%, Leiden c^27^: 23%, see Supplementary Data 1 and 2) into the analysis. We excluded these three samples from the other analyses to avoid reducing the number of SNPs in the dataset. PCA showed that Leiden b and Honshu wolf were very close to the Japanese wolf cluster, while Leiden c was placed at an intermediate position between dogs and Japanese wolves (Supplementary Fig. 6). ADMIXTURE analysis showed that Leiden b and Honshu wolf exhibit the same ancestry pattern as the other Japanese wolf individuals, while Leiden c seemed to contain the dog genetic compositions (Supplementary Fig. 7). We used Patterson’s *f*4 statistic^28^ to identify dog individuals with high genetic affinity to Leiden c to see which dog population was the source of the introgression. The dog that showed the highest affinity to Leiden c was the Japanese dog Shiba (Supplementary Fig. 8A), and Leiden c contained 39% of the Shiba’s genome (Supplementary Fig. 8B). Note that the modern Shiba breed was not assigned until the 1930s and 1940s, well after the birth of the 19th-century Leiden c specimen. Therefore, we presumed that the source dog that provided the dog genome for the Leiden c individual was an indigenous dog of the 19th century. In contrast, Leiden b showed no affinity with dogs (Supplementary Fig. 8C). These results indicate that Leiden b and Honshu wolf are included in the group of Japanese wolves, while Leiden c is a hybrid individual between Japanese wolves and dogs.

To confirm a previous study^23^ analyzing Honshu wolf, we added the Pleistocene wolves (Bunge-Toll-1885, Tirekhtyakh, Tumat 2, Ulakhan Sular, Yana RHS in Supplementary Data 2)^20,21^ to our dataset and performed PCA (Supplementary Fig. 9). Pleistocene wolves were closely related to Eurasian wolves, while Japanese wolves formed a distinct cluster. Our ADMIXTURE analysis (Supplementary Fig. 7) suggests that Honshu wolf does not contain more DNA components of dogs than the other Japanese wolf individuals. These results differed from Niemann et al. 2020, which analyzed a single Honshu wolf. The difference between the Niemann et al. 2020 and our data is in the number of Japanese wolf individuals and sequence coverage. Therefore, we modified our analysis to create a total of eight datasets, seven with the number of Japanese wolves ranging from 1 to 7 and one with only one Honshu wolf instead of Japanese wolves. The results of PCA and ADMIXTURE using these eight data sets (Supplementary Figs. 10–12) showed that the differences between the results of Niemann et al. 2020 and this study were mainly due to the number of Japanese wolves used in the analysis. Differences in the coverage of the DNA sequences or the genetic affinity between the Honshu Wolf and Pleistocene wolves^24^ likely have a small effect on these differences (see detailed explanation in Supplementary Note 1).

To further confirm previous studies proposing gene flow between the Japanese wolf and the Pleistocene wolves^24^, we added the Pleistocene wolves (aka_Bunge_Toll, BelayaGora, JK2183, and PJ35k in Supplementary Data 2) and 5000-year-old Japanese wolf (Jw5k in Supplementary Data 2) to our dataset (Supplementary Figs. 13–18). Each Honshu wolf and Jw5k formed a monophyletic group with other Japanese wolves with a high bootstrap support (Supplementary Fig. 13B and C), respectively, suggesting that these two individuals are a member of the Japanese wolf. In addition, *f4*-statistics suggested that gene flow between the Pleistocene wolves and the Japanese wolf (Supplementary Fig. 14A) and the Honshu wolf (Supplementary Figs. 14B and 16B) were under the detection limit (see detailed explanation in Supplementary Note 2).

### Phylogenetic position of the Japanese wolves

**Fig. 2:**
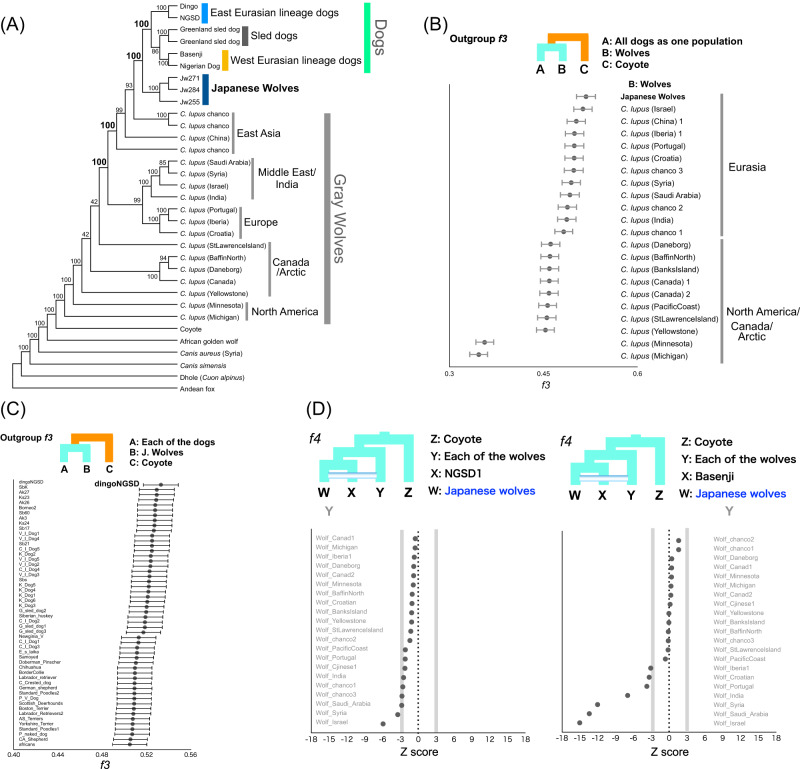
Phylogenetic relationships and genetic affinity between Japanese wolves and other canids. **A** Maximum likelihood tree based on 17,489 unlinked biallelic SNPs extracted from 179,397 sites (see Supplementary Data 2 for sample information) **B** Shared genetic drift between dogs and gray wolves measured by outgroup *f*3 statistics. Each of all dogs and Japanese wolves were used as populations. The wolves are shown on the right side of the panel. Data are presented as *f3* values +/− standard errors (*n* = 52) (represented by error bars). **C** Shared genetic drift between Japanese wolf and all dogs measured by outgroup *f*3 statistics. Each of the African dogs and Dingo/NGSD individuals were used as populations. The names of the dogs are shown on the left side of the panel. Data are presented as *f3* values +/− standard errors (*n* = 9) (represented by error bars). **D** *f4* statistics testing the relationships between the Japanese wolf and all other wolves compared with NGSD1 (left panel) and Basenji (right panel). The names of wolves are shown on the left and right sides of the left and right panels, respectively. Gray lines show the Z score −3 and 3 (*n* = 10). Source data are provided as a Source Data file.

To determine the phylogenetic position of the Japanese wolves, a phylogenetic tree was constructed using the maximum likelihood (ML) method (Fig. 2A). Among gray wolves, North American/Arctic individuals branched off first at the basal position of the tree, followed by European/Middle Eastern and East Asian gray wolves (also see Supplementary Fig. 19). Dogs formed a monophyletic clade (Fig. 2A and Supplementary Fig. 19), as shown in previous studies^2,3,12,16,18,20^. Japanese wolves formed a monophyletic clade that was a sister group to the monophyletic clade of dogs (Fig. 2A and Supplementary Fig. 19). The sister group relationship between Japanese wolves and dogs was also supported by a tree inferred by SVDQuartets, a Neighbor-Joining tree based on identity-by-state (IBS) distance, a Maximum parsimony tree, a species-tree type phylogenetic tree based on coalescent, and a ML tree without minor allele frequency cut off and pruning (Supplementary Figs. 20–24). We confirmed that the Pleistocene wolves belonged to the Eurasian gray wolf clade and were distantly related to the dog/Japanese wolf clade (Supplementary Fig. 15). Analysis using outgroup-*f3* statistics^28^ also showed that the Japanese wolf was the most closely related to dogs among wolves (Fig. 2B). When we further divide the dogs into subpopulations, outgroup-*f3* statistics showed different results between dingo/NGSD and African dogs; dingo/NGSD is related most closely to Japanese wolf while African dog is related most closely to the Middle Eastern gray wolves (Supplementary Fig. 25A and B). The different genetic affinities of dog populations to the Japanese wolf may have resulted from introgression between African dogs and Middle Eastern gray wolves^10,13,29^.

Since the tree topology in phylogenetic analyses could be affected by introgression between taxa, a phylogenetic tree using taxa showing minimal introgression effects is expected to be the most accurate representation of population branching. Therefore, in order to obtain such a tree, we examined introgression between Japanese wolves and other dogs and wolves. We compared the genetic affinity of each dog with the Japanese wolves using *f3* and *f4* statistics, and found that dogs of the East Eurasian lineage (Supplementary Figs. 19–21), in particular, dingoes, NGSDs, and Japanese dogs, showed significant affinity with the Japanese wolves (Z score > 3) (Fig. 2C and Supplementary Figs. 26 and 27). In contrast, dogs of the west Eurasian lineage, in particular dogs from Africa, showed low affinity to Japanese wolves (Fig. 2C and Supplementary Figs. 26 and 28). *f4* statistics showed no affinity between any of the gray wolf populations and Japanese wolves (Fig. 2D).

Possibilities of gene flow between gray wolves except for the Japanese wolf and dogs were also examined using *f4* statistics. Gray wolves in the Middle East showed strong affinity with dogs (Supplementary Fig. 29), consistent with previous reports^10,13,29^. Based on these results, we reperformed phylogenetic analysis to confirm the relationship between Japanese wolves and dogs. To minimize the effect of introgression between wolves and dogs, we included African dogs as the sole representatives of dogs, and excluded gray wolves from the Middle East. Even in the phylogenetic tree obtained from this analysis, the Japanese wolf still formed a sister clade with African dogs (Supplementary Fig. 30). We also confirmed the Japanese wolf and the dog relationship by constructing phylogenetic trees using Japanese dogs excluded (Supplementary Fig. 31A) and East Eurasian dogs excluded (Supplementary Fig. 31B) data sets. Thus, we concluded that the most closely related wolves to the dog lineage are the Japanese wolves.

### The genome of the Japanese wolf ancestor in the dog genome

**Fig. 3:**
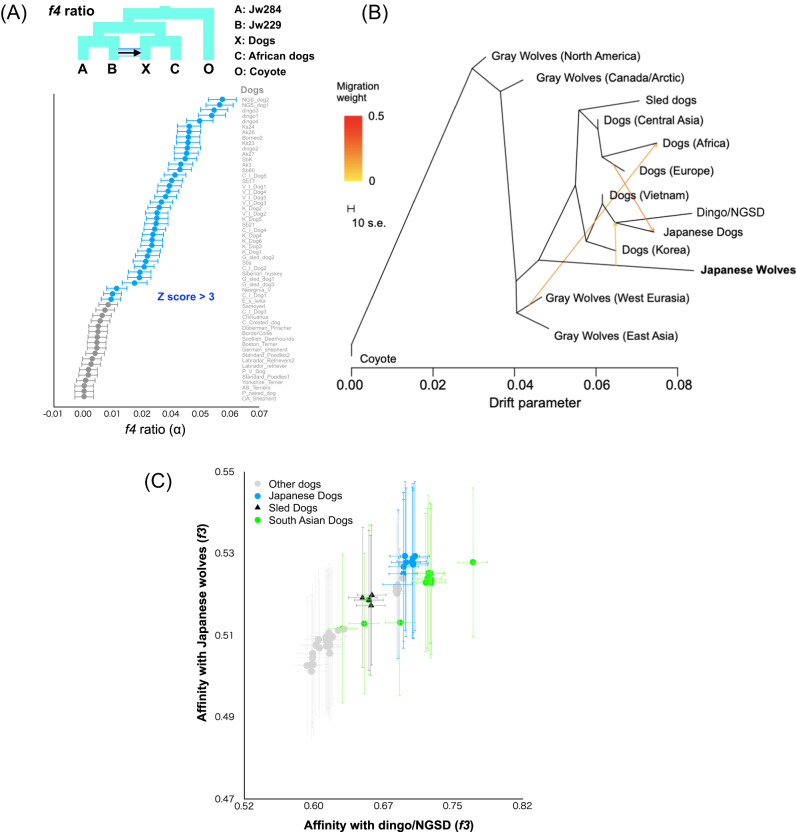
Admixture between Japanese wolves and the other canids. **A** *f4*-ratio test to estimate proportion of genome introgression from the Japanese wolf to dogs. Each *f4*-ratio α value is plotted in order of highest to lowest value from the top, and the names of the dogs are shown on the right side of the panel. Data are presented as *f4*-ratio α values +/− standard errors (*n* = 14) (represented by error bars). Z score above 3 is colored in blue. **B** TreeMix admixture graph built using LD-pruned data (150,502 sites) on a dataset consisting of 88 dogs/wolves merged into 13 groups according to the phylogenetic relationship. **C** *f3* statistics testing whether dogs share more alleles with dingo/NGSD (x-axis) or Japanese wolf (y-axis). Dots show the *f3* statistics, and horizontal and vertical error bars represent standard errors for tests with the African dogs (x-axis, *n* = 12) and dingo/NGSD (y-axis, *n* = 8), respectively. Data are presented as *f3* values +/− standard errors. Each of the Japanese wolves and dingo/NGSD individuals were used as populations. Source data are provided as a Source Data file.

Japanese wolves showed strong affinity with many East Eurasian dogs (*f3*, *f4* statistics) (Fig. 2C and Supplementary Figs. 26–28), which may be caused by the introgression of dog genomes into the Japanese wolf or its ancestor (hereafter, the Japanese wolf ancestry) or vice versa. We investigated the direction of gene flow between Japanese wolves and East Eurasian dogs using the *f4*-ratio^28^. We found that the degree of genome introgression from the Japanese wolf lineage to dogs was the highest in dingoes and NGSDs (5.5%) followed by Japanese dogs (3–4%), as well as in dogs of other East Eurasian lineages (Fig. 3A). In contrast, genomic introgression from dogs to the Japanese wolf genome was not supported (Supplementary Fig. 32), and the Japanese wolf genome contains a small proportion of the dog genome that is undetectable by *f*4-ratio (Supplementary Figs. 33 and 34).

The degree of genomic introgression from Japanese wolves to dogs was higher in East Eurasian than in West Eurasian dogs. It also varied among the dogs of East Eurasia. This variation may have been caused by multiple introgression events between the ancestors of Japanese wolves and dogs in different regions, or by a single introgression followed by diffusion of the Japanese wolf ancestry genome into various dog populations.

To determine which hypothesis is more likely, we first examined the degree of gene flow among dogs in different regions. African dogs and dingo/NGSD represent opposite edges of the dog cluster in the PCA (Fig. 1A), and show the lowest and highest affinities with Japanese wolves, respectively (Fig. 2C). Among dogs, African dogs show the lowest affinity with dingo/NGSD and dingo/NGSDs show the lowest affinity with African dogs (Supplementary Figs. 35 and 36). Dingoes are estimated to have diverged from South Asian dogs 8300 years ago (CI: 5400–11200)^30^, with archaeological evidence supporting their arrival in Australia at least 3500 years ago^31^ or 3348−3081 years ago^32^. It is considered that the dingoes have been isolated in Australia since then^1,5^. African dogs are estimated to have diverged from European breeds 14,000 years ago^33^ with archaeological evidence dating the earliest dog in Africa at 6300-5600 BC^34^. These studies also indicate that they have remained isolated since this time^1,5^. The African Dog and Dingo/NGSD are included in the West Eurasian and East Eurasian clades, respectively (Supplementary Figs. 19–21). Therefore, they are likely to be the oldest diverging lineages in their respective clades.

The *f4* statistics biplot showed that dogs showing higher affinity with dingo/NGSD show lower affinity with African dogs while dogs showing higher affinity with African dogs show lower affinity with Dingo/NGSD (Supplementary Fig. 37). This negative correlation suggests most dog populations were formed through extensive past mixing between East and West Eurasian lineages represented by dingo/NGSD and African dogs, respectively. Indeed, several dogs in South and East Asia are genomically characterized as dingo/NGSD admixed with African dogs by negative values of *f3* statistics^28^ (Supplementary Fig. 38).

**Fig. 4:**
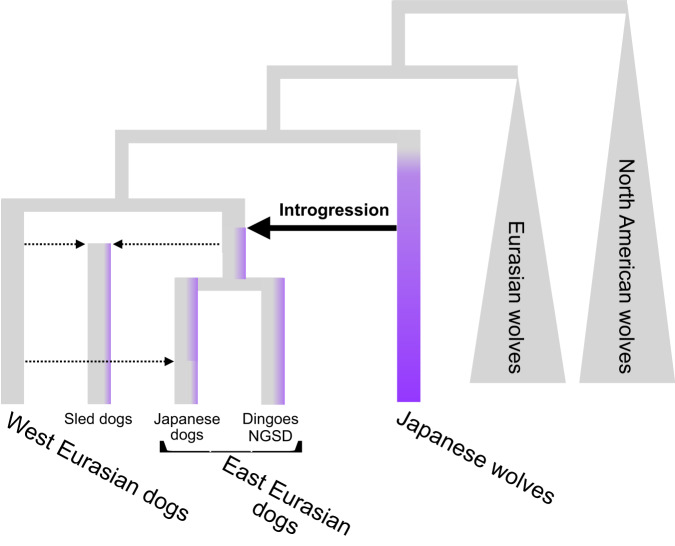
A model of Japanese wolf introgression. Dotted and solid lines indicate introgression events. Purple represents the Japanese wolf genome.

Next, we examined the degree of introgression between dogs from different regions and Japanese wolf ancestry. TreeMix analysis indicates an introgression from the ancestor of Japanese wolves into the common ancestor of dingo/NGSD and Japanese dogs (Fig. 3B). The *f3* biplot of affinities with dingo/NGSD and with Japanese wolves (Fig. 3C) shows a positive correlation among dogs, indicating that the Japanese wolf ancestry genome has become widespread through the admixture between West and East Eurasian dog lineages and persists in the modern genomes of the East Eurasian lineage (see Supplementary Fig. 39 and the legend of Supplementary Fig. 39 for a detailed explanation of this event). Therefore, it is likely that the genome of the Japanese wolf ancestor was introgressed into an ancestral lineage of the East Eurasian lineage after the split of West and East Eurasian lineages (Fig. 4). Subsequently, the East Eurasian lineage containing the Japanese wolf ancestry admixed with the West Eurasian lineage, resulting in differences in affinities with the Japanese wolves. Hence, it is likely that the difference in the degree of genomic introgression from Japanese wolves to dogs was caused by a single introgression followed by diffusion of the Japanese wolf genome into various dog populations.

## Discussion

The Japanese wolves are likely to have been isolated in the Japanese archipelago until their extinction only 100 years ago. This study reveals that they form a monophyletic group with no evidence of gene flow with other Eurasian gray wolves.

The tree topology in phylogenetic analyses could be affected by introgression between taxa because the introgressed genomic regions make the taxa more closely related. Therefore, if we exclude introgressed genomic regions from the phylogenetic analysis, we can reconstruct the most accurate phylogenetic relationships. In this study, we showed an introgression from the ancestor of the Japanese wolf to the ancestor of the East Eurasian dog. We reconstructed a phylogenetic tree using only African dogs to exclude the introgressed genomic regions from the phylogenetic analysis. The tree showed that the Japanese wolf and dog are most closely related. Therefore, we conclude that the dog lineage has diverged from the common ancestor of the dog and the Japanese wolf.

One notable aspect of Japanese wolves is their phylogenetic position. In Eurasia, our phylogenetic analysis showed that the European/Middle East lineage of the gray wolves diverged at the basal position, followed by the East Asian lineage. In the East Asian lineage, the monophyletic group of Japanese wolves and the dog lineage form a sister-group relationship. The order in which Eurasian wolf lineages diverged is from west to east in geographical order on the Eurasian continent. Considering these phylogenetic and geographic relationships, it is most likely that it was in East Asia that the divergence between the Japanese wolf and the dog lineages has occurred. In other words, the extinct population of the gray wolf from which dogs are suspected to have been domesticated^1,3,5,9,11,12^ was closely related to the ancestor of the Japanese wolf and was likely to inhabit East Asia. This hypothesis does not directly imply that the origin of dog domestication was in East Asia. Although the domestication process would have been initiated with the animals’ association with humans^5^, our phylogenetic analyses provide no evidence for when dog lineages began to associate with humans. Further archaeological evidence in the studies of ancient “proto-dog” populations are required to clarify the beginnings of the dog-human relationship.

This study suggests ancient genomic introgression from the Japanese wolf ancestry to dogs, most likely to the ancestor of the East Eurasian lineage. The divergence between the dog lineage and the Eurasian gray wolves has been estimated to be 20,000-40,000 years ago^11,18^. Dogs have been reported to have split into West Eurasian, East Eurasian, and sled dog lineages in their early divergence^2,3,12,16,18^. A 9500-year-old sled dog (ca. 9514.5 years ago: radiocarbon dating)^20^ already contained the same proportion of the Japanese wolf ancestry genome as the modern sled dog (2%: Supplementary Data 3, Supplementary Fig. 40). Therefore, the genomic introgression of the ancestor of the Japanese wolf to the East Eurasian lineage of dogs must have occurred before the establishment of the sled dog lineage at least 9500 years ago (before the radiocarbon date) during the transitional period from the Pleistocene to the Holocene and shortly after the divergence of the East and West Eurasian dog lineages. We estimate that the genome of NGSD contains 5.5% of the Japanese wolf genome. It is estimated that the NGSD lineage already existed by 10,900 years ago^29^, which also supports the hypothesis that the introgression from the ancestor of Japanese wolves into dogs had occurred in the Pleistocene. The ancient dog genome data from two European individuals (4800 and 7000 years ago) already contained about 1.6% of the Japanese wolf ancestry genome (Supplementary Data 3, Supplementary Fig. 40). Since the gene flow from the Southeast Asian dog ancestry to the ancestor of these two ancient European dogs has been reported^18^, the genome of the Japanese wolf ancestry may have introgressed into European dogs via the Southeast Asian dog ancestry more than 7000 years ago.

The oldest Japanese dog excavated from the Natsushima shell mound is morphologically similar to other examples of dogs from the Jomon Period^35^, and was estimated to be the same age as the shells (9450 ± 400 years ago: radiocarbon dating) and charcoal (9240 ± 500 years ago: radiocarbon dating) from the same layer at this site^36^. The genome of an ancient sled dog (9514.5 years old)^20^ already contained the genome of the Japanese wolf ancestry. These two contemporaneous ancient dogs strongly suggest that introgression between the ancestor of Japanese wolves and dogs of the East Eurasian lineage had occurred before dogs were brought to the Japanese archipelago. Therefore, the introgression between the ancestral Japanese wolf and the East Eurasian lineage of dogs is most likely to have occurred somewhere in East Asia. The high proportion of the Japanese wolf ancestry genome in the dingo/NGSD (5.5%) is inferred to be due to their isolation in the islands of Southeast Asia and Australia, where they have escaped admixture with the West Eurasian dog lineage.

In this study, we demonstrated that the Japanese wolf is a sister group with the monophyletic clade of dogs. Our results support the hypothesis that the modern dog lineage was domesticated from an extinct population of gray wolves^1,3,5,9,11,12^, and the Japanese wolf is the closest to this now-extinct gray wolf population. In addition, we estimated the levels of introgression from the ancestor of Japanese wolves to the ancestor of East Eurasian dogs. Accordingly, the ancestor of the Japanese wolf genome is expected to be involved in the early stages of dog domestication. Further analysis of the genome of the Japanese wolf and ancient dog genomes, in particular from East Eurasia, will shed further light on the origins of dog domestication.

## Methods

### Samples, DNA extraction, and sequencing

Permission to analyze the Japanese wolf DNA was obtained for Jentink b (Leiden b): RMNH.MAM.39183, and Jentink c (Leiden c): RMNH. MAM.39181(52994) in Naturalis Biodiversity Center, Leiden, the Netherlands, and Jw284 (ZMB48817): ZMB_Mam_48817 in Museum für Naturkunde, Berlin, Germany. Six Japanese wolf samples from personal collections (Jw229, Jw255, Jw258, Jw269, Jw271, and Jw275) were analyzed with the permission of the owners. Blood or saliva samples for Japanese dogs were analyzed with the permission of the owners. Japanese Wolf DNAs were extracted and used in our previous mitochondrial DNA studies: Ishiguro et al. 2009 (Jw229, Jw255, and Jw258)^22^, Ishiguro et al. 2016 (Jw269 and Jw271)^37^, and Matsumura et al. 2021 (Jw275, Jw284, Leiden b, and Leiden c)^25^. The sample locations are listed in Supplementary Data 1. Bone powder (0.1 to 0.3 g) was obtained from the mandible (Jw229, Jw255, Jw258, Jw269) and Cranium (Jw271 and Jw275) specimens by using an electric drill after removal of the outer layers of bone by scraping with a sterile razor blade. Powders were also obtained from ventral nasal concha specimens (Jw284, Leiden b, and Leiden c) with a Multi-beads Shocker. According to methods described by Okumura et al. (1999)^38^, all bone powders were suspended in 10 ml of 0.5 M ethylenediamine tetraacetic acid (EDTA) at pH 7.0, and rotated for 24 hours for decalcification. This procedure was repeated several times until the supernatant became clear. The pellets of bone powder were collected by centrifugation, and the decalcification was repeated by washing with 10 ml of 0.5 M EDTA at pH 7.0 until the supernatant was clear. The samples were then treated with 5 ml of 0.5 M EDTA with proteinase K (300 μg/ml) and N-lauryl sarcosine (0.5%) for 24 hours. After centrifugation, the supernatant containing DNA was extracted twice with phenol, once with chloroform/phenol (1:1), and once with chloroform to remove protein. The supernatant was concentrated by using a Centricon 30 spin column (Amicon, Beverly, MA).

In recent years, Japanese dog breeding has become popular in Japan, and wide varieties of Shiba have been seen, including miniature Shiba and white or black Shiba (Shiba was initially light brown). However, what breeds were crossbred with Japanese dogs was unknown. Therefore, DNA extracted 26 years ago^39^ was used to avoid the effects of recent crossbreeding between Japanese dogs and other dog breeds for the eight modern dogs (Akita26, Akita27, Akita3, Kishu23, Kishu24, Shiba17, Shiba21, and Shiba60). Blood samples for these individuals were provided by the veterinary clinics with the permission of the owners. Peripheral blood leukocytes were suspended in a lysis buffer containing proteinase K (1 mg/ml). Genomic DNA was extracted twice with phenol, once with chloroform/isoamyl alcohol (24:1), then precipitated by ethanol.

Blood samples for two individuals of Shiba (Shiba_shiro and Shiba_kuro) were provided by the veterinary clinics with the permission of the owners. The saliva of an individuals of Shiba (Jm) was scrubbed by the owner with cotton swabs. DNAs of these three individuals of Shiba were extracted using a DNeasy Blood & Tissue Kit (Qiagen) following the manufacturer’s instructions. Paired-end (2 × 150 bp) sequencing was performed on the Illumina HiSeq X or NovaSeq 6000 platforms. The Institutional Animal Care and Use Committee of Anicom Specialty Medical Institute approved the animal protocols and procedures (No. 2020-02).

For Leiden b and Leiden c, the genome capture was performed using the SeqCap EZ Hybridization and Wash Kit (Roche, Basel, Switzerland), SeqCap EZ Accessory Kit v2 (Roche), SeqCap HE-Oligo Kit (Roche), and SeqCap EZ Pure Capture Bead Kit (Roche) following the manufacturer’s instructions for SeqCap EZ Library SR (Roche), with minor modifications. Briefly, Biotin-labeled genomic DNA fragments from Shiba were used as hybridization probes, instead of the SeqCap EZ library (Roche). Leiden b and Leiden c libraries were mixed with 135 ng of Biotin-labeled genomic DNA fragments and were hybridized at 47 °C for 72 h. Other procedures were performed in accordance with the manufacturer’s instructions. Total mapped reads are listed in Supplementary Data 1.

### Extraction of SNPs and vcf file preparation

We downloaded sequencing data of 56 modern dogs, 23 modern gray wolves, four ancient dogs, five ancient canids, and six outgroup species from the database (Supplementary Data 2). Sequence reads from the genomic DNA libraries of nine Japanese wolves, eleven Japanese dogs (Supplementary Data 1) as well as 94 samples from the database (Supplementary Data 2) were trimmed to remove nucleotides with base qualities lower than 35 on average of a 150 bp read (sum of the base-calling error probability <0.05 in a 150 bp read) and adaptor sequences using CLC Genomics Workbench (https://www.qiagenbioinformatics.com/). The trimmed reads were mapped to the dog reference genome (CanFam3.1) using CLC Genomics Workbench. Reads showing high similarity ( > 90% in > 90% of read length) were mapped to the reference genome sequences to avoid mapping the low similarity reads. Reads mapped to more than one position were removed (“ignore” option for reads mapped to multiple positions) to prevent mapping to non-unique regions. The mapping data was exported in bam file format and sorted and indexed using samtools^40^. The duplicated reads in bam files were marked by the MarkDuplicates algorithm implemented in GATK v4.2 (https://gatk.broadinstitute.org/hc/en-us). We performed genotype calling on all individuals analyzed in this study using the HaplotypeCaller algorithm in GATK v4.2. Genotypes of all individuals were output as gvcf format (-ERC GVCF option). All gvcf files were combined into a single gvcf format file by the CombineGVCFs algorithm in GATK v4.2. The combined file was genotyped by the GenotypeGVCFs algorithm and filtered by Filtervcf in GATK v4.2 with parameters; --filter-expression “QD<2.0” --filter-name “QD2” --filter-expression “QUAL<30.0” --filter-name “QUAL30” --filter-expression “FS>200.0” --filter-name “FS200” --filter-expression “SOR>10.0” -filter-name “SOR10” --filter-expression “ReadPosRankSum<−20.0” --filter-name “ReadPosRankSum-20”.

Following the method of vonHoldt et al. (2010)^10^, we excluded the sample pairs that show IBS > 0.8 within groups (the same dog breeds or wolves from the same location). IBS values were obtained by the “—distance ibs” function of plink^41^. The IBS of two wolf pairs, Wolf_Chinese1-Wolf_Chinese2 and Wolf_Iberia1-Wolf_Iberia2, show higher IBS than the threshold (0.8). Therefore, we excluded Wolf_Chinese2 and Wolf_Iberia2 from all of our analyses.

To maximize the number of SNPs for analyses, we prepared datasets from the genotyped vcf file for each analysis by following filtering using vcftools^42^.

#### Dataset 1: PCA and ADMIXTURE using the Japanese wolf (excluding Leiden b, Leiden c, and a Honshu wolf) and modern samples (Fig. 1, Supplementary Figs. 4 and 5)

We removed four ancient dogs, five ancient canids, three Japanese wolves (Leiden b, Leiden c, and a Honshu wolf), two modern wolves (a Chinese wolf and an Iberian wolf), and three outgroup species (Andean fox, Dhole, and Ethiopian wolf) from the genotyped vcf file (Supplementary Data 2). We removed all sites with missing data. Then, we removed all indels, singleton, and doubleton sites to eliminate PCR and sequencing errors that may have occurred in one individual by minor allele frequency (MAF) filtering (MAF < 0.015). We extracted bi-allelic sites with coverage equal to or more than three in all individuals and with GQ values equal to or more than 20 in all individuals. Mutations due to DNA damage at both ends of fragments were less than 1% in Japanese wolves (Supplementary Fig. 1), therefore we can infer that mutations by DNA damage in the sequences of Japanese wolves were removed by this filtration. The final dataset consisted of 1,696,115 sites (97 individuals).

#### Dataset 2: PCA and ADMIXTURE using the Japanese wolf (including Leiden b, Leiden c, and a Honshu wolf) and modern samples (Supplementary Figs. 6 and 7)

We removed four ancient dogs, five ancient canids, two modern wolves (a Chinese wolf and an Iberian wolf), and three outgroup species (Andean fox, Dhole, and Ethiopian wolf) from the genotyped vcf file (Supplementary Data 2). We removed sites with missingness higher than 3% and minor allele frequency (MAF) < 0.04. We extracted bi-allelic sites with coverage equal to or more than three in all individuals and with GQ values equal to or more than eight in all individuals. The final dataset consisted of 342,931 sites (100 individuals).

#### Dataset 3: PCA using the Japanese wolf (excluding Leiden b, Leiden c, and a Honshu wolf), ancient dogs, ancient canids, and modern samples (Supplementary Fig. 9)

We removed two modern wolves (a Chinese wolf and an Iberian wolf), three Japanese wolves (Leiden b, Leiden c, and a Honshu wolf), and three outgroup species (Andean fox, Dhole, and Ethiopian wolf) from the genotyped vcf file (Supplementary Data 2). We removed all sites with missing data. Then, we removed all indels, singleton, and doubleton sites to eliminate PCR and sequencing errors that may have occurred in one individual by minor allele frequency filtering (MAF < 0.01). We extracted bi-allelic sites with coverage equal to or more than three in all individuals and with GQ values equal to or more than eight in all individuals. We extracted transversion sites to eliminate the effect of DNA damage in the ancient samples. The final dataset consisted of 100,588 sites (106 individuals).

#### Dataset 4: PCA and ADMIXTURE using the Japanese wolf (excluding Leiden b, and Leiden c), ancient canids, and modern samples (Supplementary Figs. 10–12)

We removed two modern wolves (a Chinese wolf and an Iberian wolf), and three outgroup species (Andean fox, Dhole, and Ethiopian wolf) from the genotyped vcf file (Supplementary Data 2). We modified this dataset to create a total of eight datasets, seven with the number of Japanese wolves ranging from 1 to 7 and one with only one Honshu wolf instead of Japanese wolves. We removed all sites with missing data. Then, we removed all indels and filtered by minor allele frequency filtering (MAF < 0.05). We extracted bi-allelic sites with coverage equal to or more than three in all individuals and with GQ values equal to or more than eight in all individuals. We extracted transversion sites to eliminate the effect of DNA damage in the ancient samples. The numbers of sites in the eight datasets are listed in Supplementary Fig. 12.

#### Dataset 5: *f3*, *f4* statistics, and *f4*-ratio using the Japanese wolf (excluding Leiden b, Leiden c, and a Honshu wolf) and modern samples (Figs. 2B–D, 3A, C, Supplementary Figs. 25–29, 32−38)

We removed four ancient dogs, five ancient canids, three Japanese wolves (Leiden b, Leiden c, and a Honshu wolf), two modern wolves (a Chinese wolf and an Iberian wolf), and five outgroup species (Andean fox, Dhole, Ethiopian wolf, Golden Jackal, and African Golden Wolf) from the genotyped vcf file (Supplementary Data 2). We removed all sites with missing data. Then, we removed all indels, singleton, and doubleton sites to eliminate PCR and sequencing errors that may have occurred in one individual by minor allele frequency filtering (MAF < 0.01). We extracted bi-allelic sites with coverage equal to or more than three in all individuals and with GQ values equal to or more than 20 in all individuals. The final dataset consisted of 489,529 sites (95 individuals).

#### Dataset 6: *f4* statistics and *f4*-ratio using the Japanese wolf (including Leiden b or Leiden c) and modern samples (Supplementary Fig. 8)

We removed four ancient dogs, five ancient canids, a Japanese wolf (a Honshu wolf), two modern wolves (a Chinese wolf and an Iberian wolf), and five outgroup species (Andean fox, Dhole, Ethiopian wolf, Golden Jackal, and African Golden Wolf) from the genotyped vcf file (Supplementary Data 2). We modified this dataset to create two datasets, one with Leiden b and the other with Leiden c. We removed all sites with missing data. Then, we removed all indels, singleton, and doubleton sites to eliminate PCR and sequencing errors that may have occurred in one individual by minor allele frequency filtering (MAF < 0.015). We extracted bi-allelic sites with coverage equal to or more than three in all individuals and with GQ values equal to or more than eight in all individuals. The final datasets with Leiden c and Leiden b consisted of 38,254 and 83,259 sites, respectively (70 individuals).

#### Dataset 7: Phylogenetic analyses using the Japanese wolf (excluding Leiden b, Leiden c, and a Honshu wolf) and modern samples (Supplementary Figs. 19–21, 31)

We removed four ancient dogs, five ancient canids, three Japanese wolves (Leiden b, Leiden c, and a Honshu wolf), and two modern wolves (a Chinese wolf and an Iberian wolf) from the genotyped vcf file (Supplementary Data 2). We removed all sites with missing data. Then, we removed all indels, singleton, and doubleton sites to eliminate PCR and sequencing errors that may have occurred in one individual by minor allele frequency filtering (MAF < 0.01). We extracted bi-allelic sites with coverage equal to or more than three in all individuals and with GQ values equal to or more than 20 in all individuals. The final dataset consisted of 2,065,002 sites (99 individuals).

#### Dataset 8: Phylogenetic analyses using the Japanese wolf (excluding Leiden b, Leiden c, and a Honshu wolf), African dogs, modern wolves, and outgroup samples (Supplementary Figs. 30, 31)

We removed four ancient dogs, five ancient canids, three Japanese wolves (Leiden b, Leiden c, and a Honshu wolf), five modern wolves (a Chinese wolf, an Iberian wolf, and three Middle East wolves), and dogs except for African dogs from the genotyped vcf file (Supplementary Data 2). We removed all sites with missing data. Then, we removed all indels, singleton, and doubleton sites to eliminate PCR and sequencing errors that may have occurred in one individual by minor allele frequency filtering (MAF < 0.03). We extracted bi-allelic sites with coverage equal to or more than three in all individuals and with GQ values equal to or more than 20 in all individuals. The final dataset consisted of 1,916,277 sites (36 individuals).

#### Dataset 9: Phylogenetic analyses using three Japanese wolves (Jw225, Jw271, and Jw284), selected dogs (three samples each from dingo/NGSD, African, and sled dogs), modern wolves, and outgroup samples with BQSR (Fig. 2A, Supplementary Figs. 22 and 24)

After exporting bam files, marking duplicated reads, haplotype calling, combining gvcf files, and genotyping, all SNP sites were extracted. The vcf file including SNP sites was filtered by Filtervcf in GATK v4.2 with parameters; “QD<2.0” --filter-name “QD2” -filter “QUAL<30.0” --filter-name “QUAL30” -filter “SOR>4.0” --filter-name “SOR4” -filter “FS>60.0” --filter-name “FS60” -filter “MQ<40.0” --filter-name “MQ40” -filter “MQRankSum<-12.5” --filter-name “MQRankSum-12.5” --filter “ReadPosRankSum<-8.0” --filter-name “ReadPosRankSum-8”. Using the filtered vcf file as “known-sites”, base quality of the bam files of three Japanese wolves (Jw225, Jw271, and Jw284), selected dogs (three samples each from dingo/NGSD, African, and sled dogs), modern wolves, and outgroup samples (Supplementary Data 2) were recalibrated using the BaseRecalibrator and the ApplyBQSR algorithm in GATK v4.2 (BQSR). The recalibrated bam files were genotype called again, and output in gvcf format using the HaplotypeCaller algorithm in GATK v4.2. All gvcf files were combined into a single gvcf format file by the CombineGVCFs algorithm, and a combined file was genotyped by the GenotypeGVCFs algorithm in GATK v4.2. We removed all sites with missing data. Then, we removed singleton and doubleton sites to eliminate PCR and sequencing errors that may have occurred in one individual by minor allele frequency filtering (MAF < 0.035). We extracted bi-allelic sites with coverage equal to or more than three in all individuals and with GQ values equal to or more than 20 in all individuals. The final dataset consisted of 179,397 sites (33 individuals). For a Maximum parsimony tree (Supplementary Fig. 22), we extracted bi-allelic sites with coverage equal to or more than five in all individuals and with GQ values equal to or more than 20 in all individuals without minor allele frequency filtering (the final dataset: 4,864,602 SNPs).

#### Dataset 10: Phylogenetic analyses using the Japanese wolf (including a Honshu wolf) and modern samples (Supplementary Fig. 13B)

We selected six modern dogs, ancient dogs, four Japanese wolves (Jw255, Jw271, Jw284, and a Honshu wolf), and modern wolves from the genotyped vcf file (Supplementary Data 2). We removed all sites with missing data. Then, we removed all indels, singleton, and doubleton sites to eliminate PCR and sequencing errors that may have occurred in one individual by minor allele frequency filtering (MAF < 0.04). We extracted bi-allelic sites with coverage equal to or more than three in all individuals and with GQ values equal to or more than eight in all individuals. The SNP dataset was pruned and the final dataset consisted of 60,512 sites (36 individuals).

#### Dataset 11: Phylogenetic analyses using the Japanese wolf (including Jw5k) and modern samples (Supplementary Fig. 13C)

We selected six modern dogs, ancient dogs, four Japanese wolves (Jw255, Jw271, Jw284, and a Jw5k), and modern wolves from the genotyped vcf file (Supplementary Data 2). We removed all sites with missing data. Then, we removed all indels, singleton, and doubleton sites to eliminate PCR and sequencing errors that may have occurred in one individual by minor allele frequency filtering (MAF < 0.04). We extracted bi-allelic sites with coverage equal to or more than three in all individuals and with GQ values equal to or more than eight in all individuals. We extracted transversion sites to eliminate the effect of DNA damage in the ancient samples. The final dataset consisted of 1019 sites (36 individuals).

#### Dataset 12: Phylogenetic analyses, *f3*, and *f4* statistics using the ancient canids and modern samples (Supplementary Figs. 14A, 15, 17, and 18)

We selected six modern dogs, ancient dogs, eight ancient canids, three Japanese wolves (Jw255, Jw271, and Jw284), and modern wolves from the genotyped vcf file (Supplementary Data 2). We removed all sites with missing data. Then, we removed all indels, singleton, and doubleton sites to eliminate PCR and sequencing errors that may have occurred in one individual by minor allele frequency filtering (MAF < 0.03). We extracted bi-allelic sites with coverage equal to or more than three in all individuals and with GQ values equal to or more than eight in all individuals. We extracted transversion sites to eliminate the effect of DNA damage in the ancient samples. The final dataset consisted of 164,265 sites (43 individuals).

#### Dataset 13: *f4* statistics using the Japanese wolf (including a Honshu wolf) and the ancient canids samples (Supplementary Fig. 14B)

We selected six modern dogs, ancient dogs, eight ancient canids, four Japanese wolves (Jw255, Jw271, Jw284, and a Honshu wolf), and modern wolves from the genotyped vcf file (Supplementary Data 2). We removed all sites with missing data. Then, we removed all indels, singleton, and doubleton sites to eliminate PCR and sequencing errors that may have occurred in one individual by minor allele frequency filtering (MAF < 0.03). We extracted bi-allelic sites with coverage equal to or more than three in all individuals and with GQ values equal to or more than eight in all individuals. We extracted transversion sites to eliminate the effect of DNA damage in the ancient samples. The final dataset consisted of 6190 sites (44 individuals).

#### Dataset 14: *f3* and *f4* statistics using the Japanese wolf and the ancient canids (including PJ35k) samples (Supplementary Figs. 14, 16–18)

We selected six modern dogs, ancient dogs, nine ancient canids (including PJ35k), three Japanese wolves (Jw255, Jw271, and Jw284), and modern wolves from the genotyped vcf file (Supplementary Data 2). We removed sites with missingness higher than 20% and minor allele frequency (MAF) < 0.03. We extracted bi-allelic sites with coverage equal to or more than three in all individuals. We extracted transversion sites to eliminate the effect of DNA damage in the ancient samples. The final dataset consisted of 593,0719 sites (44 individuals).

#### Dataset 15: a species-tree type phylogenetic tree based on coalescent (Supplementary Fig. 23)

We selected individuals listed in Supplementary Data 2 from dataset 1.

### Phylogenetic analysis

The SNP dataset 7 was pruned using PLINK ver. 1.9^41^ with an option “--indep-pairwise 50 10 0.1”. The pruned SNP vcf file was converted to PHYLIP format. 10 kb sequences from the 5’ end of the PHYLIP format file were extracted and a model for the Maximum Likelihood method was selected using MEGA ver. X^43^. A phylogenetic tree was constructed using the Maximum Likelihood (ML) method using PhyML ver. 3.2^44^ with a model selection option “-m GTR” and with 100 bootstrap replications (Supplementary Figs. 19: 327,402 SNPs). The same pruned-vcf file was converted to NEXUS format. A phylogenetic tree was constructed by the svdq algorithm^45^ in PAUP* ver. 4a (https://paup.phylosolutions.com) with 100 bootstrap replications (Supplementary Fig. 20: 327,402 SNPs).

Using the same pruned-vcf file, we used PLINK ver. 1.9^41^ with an option “—distance 1-ibs” to calculate an Identity By State (IBS) distance matrix using 327,402 SNPs. A neighbour joining tree was constructed from the IBS distance matrix using MEGA ver. X^43^ (Supplementary Fig. 21).

The SNP datasets 8 and 9 were pruned and a model for the Maximum Likelihood method was selected. Phylogenetic trees for each dataset were constructed using the Maximum Likelihood (ML) method described above (Fig. 2A: 17,489 SNPs and Supplementary Figs. 30 and 31: 157,906 SNPs). A phylogenetic tree for dataset 9 was constructed by the Maximum parsimony method using MEGA ver. X^43^ (Supplementary Fig. 22) with 1000 bootstrap replications.

We performed StarBeast2 (ver. 1.0.0) to obtain a species-tree type phylogenetic tree based on coalescent^46^. Because it seems that using every 30 kb window in the genome for the StarBeast2 was practically impossible due to the huge amount of required computation, we decided to sample haploblocks from the genome and use a limited number of samples instead of all the samples we have. The list of samples used (dataset 15) and the regions used are shown in Supplementary Data 2 and 4, respectively.

In order to sample haploblocks, we wish to choose genomic regions within high LD. First, we run plink with “—indep-pairwise 50 10 0.1” option against our genome wide SNPs data as if we are going to do LD pruning. As a result, we obtained a list of SNPs that are in linkage equilibrium. By using this information, we were able to know the region within high LD. Then, we randomly chose 19 regions from those haploblocks longer than 20 kb. We chose one region per one odd number chromosomes to ensure that each region is in linkage equilibrium and that we do not use too many regions for computational ease.

For performing StarBeast2, we used relatively simple models to save computational time. For setting up StarBeast2, we used BEAST software (ver. 2.7.4)^47^. We used “strict clock model” for all the loci and set the clock as “0.001”. We used HKY for the mutation model setting “Kappa=2” and “empirical.” Population model was set as “constant population.” We ran MCMC for a chain length of 1,000,000,000 to achieve at least 200 of ECC for every parameter to be estimated. We chose “Birth Death Model” for the prior of “Tree.t:Species”. For the other prior, we used the default setting. The output was checked by Tracer ver 1.7.2^48^, and we checked that every parameter has at least 200 ECC. The output trees were visualized by Densitree ver. 2.7.4^49^. In order to obtain the most plausible species tree, “Root Canal” option is used for displaying together with a sampled tree (indicated by a blue tree in Supplementary Fig. 23).

### Principal component analysis and ADMIXTURE

We performed a principal component analysis (PCA) using PLINK ver. 1.9^41^ with an option “--indep-pairwise 50 10 0.1” to explore the affinity among gray wolves, Japanese wolves, and dogs (Fig. 1A). We also performed PCA with type specimens of Japanese wolf (Supplementary Fig. 6), and with ancient canids (Supplementary Figs. 9–12).

ADMIXTURE ver. 1.3^50^ was run on the dataset of modern samples (Fig. 1B and Supplementary Figs. 4 and 5) and modern specimens with type specimens of Japanese wolf (Supplementary Figs. 7, 10–12) assuming 2 to 8 clusters (*K* = 2–8).

### *f 3*, *f4* statistics, and *f4*-ratio

*f 3*, *f4* statistics, and *f4*-ratio implemented in ADMIXTOOLS ver. 7.0.1^28^ were used to evaluate the shared genetic drift among gray wolves, Japanese wolves, and dogs using SNP dataset 5 (489,524 SNPs). For Liden b and Leiden c analyses, we prepared two vcf files to maximize the number of SNPs (dataset 6).

Outgroup *f3* statistics were calculated to explore shared genetic drift between all dogs and each of the wolves (Fig. 2B), African dogs and each of the wolves (Supplementary Fig. 25A), dingo/NGSD dogs and each of the wolves (Supplementary Fig. 25B), Japanese wolf and each of the dogs (Fig. 2C), dingo/NGSD dogs and each of the other dogs (Supplementary Fig. 35A), and African dogs and each of the other dogs (Supplementary Fig. 35B).

*f3* statistics were calculated to test the genomic mixture of African and dingo/NGSD dogs in all dogs (Supplementary Fig. 38).

*f4* statistics were calculated to explore shared genetic drift between each of the dogs and Leiden c (Supplementary Fig. 8A: 38,254 SNPs), each of the dogs and Leiden b (Supplementary Fig. 8C: 83,259 SNPs), each of the wolves and Japanese wolves (Fig. 2D), each of the dogs and Japanese wolves (Supplementary Fig. 26), each of the dogs and each of the wolves (Supplementary Fig. 29), dingo/NGSD and Japanese wolves (Supplementary Fig. 33), each of the dogs and Japanese wolves (Supplementary Fig. 34), NGSD1 or Basenji and each of the dogs (Supplementary Fig. 36), and dingoes and each of the other dogs, and African dogs and each of the other dogs (Supplementary Fig. 37).

### TreeMix

To examine the admixture events, we used TreeMix ver. 1.13^51^ to build a tree with admixture edges. We used major groups of gray wolves and dogs as follows; Gray Wolves (North America: *n* = 2), Gray Wolves (Canada/Arctic: *n* = 8), Gray Wolves (East Asia: *n* = 5), Gray Wolves (West Eurasia: *n* = 7), Japanese Wolves (Japan: *n* = 7), Dogs (Central Asian: *n* = 4), Dogs (Europe: *n* = 11), Dogs (Africa: *n* = 10), Dogs (sled dogs: *n* = 4), Dogs (Vietnamese Indigenous: n = 5), Ding/NGSD (Oceania: *n* = 7), Japanese Dogs (Japan: *n* = 11), and Dogs (Korea: *n* = 6). The SNP dataset was pruned based on linkage disequilibrium (LD-pruning) by using plink with an option “--indep-pairwise 50 10 0.12”. As a result of LD-pruning, 150,502 SNPs were used for TreeMix.

### Assessing DNA damage patterns

We used mapDamage ver. 2.2.0^52^ to assess DNA damage patterns in the Japanese wolf samples sequenced in this study. Mapped reads from the Japanese wolf samples showed slightly increased proportion (equal to or less than 1%) of C to T and G to A substitutions at the 5’ and 3’ read ends, respectively (Supplementary Fig. 1).

### Calculation of maximum contamination rate

We used substitutions in mitochondria DNA specific to Japanese wolf to assess the contamination rate of the other animal DNA in Japanese wolf DNA. Fifteen fixed substitutions unique to Japanese wolf were selected using an alignment of mitochondria DNA sequences including gray wolves, Japanese wolves, and dogs used in previous studies^25,53^. The lowest coverage at fifteen sites was 48 (highest was 28,324) in the mapping result to the mitochondria genome in CanFam3.1. We calculated the average mapping ratio in fifteen sites. The ratio of the reads mapped to the fifteen sites without substitutions specific to the Japanese wolf were assumed as the maximum contamination rate (Supplementary Data 5), because the mitochondria DNA-like sequences are found in the nuclear genome.

### Reporting summary

Further information on research design is available in the Nature Portfolio Reporting Summary linked to this article.

### Supplementary information

Supplementary Information

Description of Additional Supplementary Files

Supplementary Data 1

Supplementary Data 2

Supplementary Data 3

Supplementary Data 4

Supplementary Data 5

Reporting Summary

### Source data

Source data

## Data Availability

The authors declare that the data supporting the findings of this study are available within the paper and its Supplementary Information files. The nucleotide sequences of the Japanese wolves and Japanese dogs were deposited in the DDBJ Sequenced Read Archive under a Bioproject PRJDB12777. The other sequence data were from public database with the accession numbers listed Supplementary Data 2. All other data are provided in the supplementary information files. Source data are provided as a Source Data file. Source data are provided with this paper.

## Code availability

The analyses presented in this manuscript did not involve any custom code or custom statistical algorithms and did not involve any modifications to the codes/packages used.
