# Supplementary material for: Japanese wolves are most closely related to dogs and share DNA with East Eurasian dogs
Source: Nat Commun. 2024 Feb 23;15:1680. doi: 10.1038/s41467-024-46124-y (PMC10891106; doi:10.1038/s41467-024-46124-y)
Supplement: Supplementary file 1 — Supplementary Information [file 41467_2024_46124_MOESM1_ESM.pdf]

Supplementary Information for

Japanese wolves are most closely related to dogs and share DNA with East Eurasian dogs

Jun Gojobori[1†], Nami Arakawa[1], Xiayire Xiaokaiti[1], Yuki Matsumoto[2], Shuichi Matsumura[3], Hitomi Hongo[1], Naotaka Ishiguro*[1, 3], Yohey Terai*[1†]

[1]SOKENDAI (The Graduate University for Advanced Studies), Research Center for Integrative Evolutionary Science, Shonan Village, Hayama, Kanagawa 240-0193, Japan.

[2]Research and Development Section, Anicom Specialty Medical Institute. Naka-ku, Chojamachi Yokohama 231-0033, Japan.

[3]Faculty of Applied Biological Sciences, Gifu University, Yanagido 1-1, Gifu 501-1193, Japan.

TABLE OF CONTENTS

Supplementary Notes — Pages S2-S3

Supplementary Figures. — Pages S4-43

Supplementary References — Page S44

Supplementary Note 1

The effect of the number of Japanese wolf individuals on the analyses.

In Supplementary Figure 10A, the PCA showed that the Japanese wolf plot was closer to the Pleistocene wolf plots in the dataset that included only one Japanese wolf. This result is most likely due to the lack of population-specific variations in the Japanese wolf. In the ADMIXTURE result, the Japanese wolf was represented by a gray wolf and a genetically related dog genetic composition due to the absence of Japanese wolf population-specific variation (Supplementary Figure 10A). As the Japanese wolf numbers increased from 2 to 7, the Japanese wolf cluster in PCA separated from Pleistocene wolves and other gray wolves (Supplementary Figure 10B, 10C, 11A-11C, 12A). They formed an independent cluster in ADMIXTURE results when the Japanese wolf population was 6-7 individuals (Supplementary Figure 11C, 12A). This independency of the Japanese wolf population is estimated to be due to an increase in variations that the Japanese wolf population has at high frequencies and variations that only the Japanese wolf population does not have as the Japanese wolf population increases. The number of sites in the dataset increased as the Japanese wolf population increased (Supplementary Figure 12: table).

In the dataset with only the Honshu wolf, the Honshu wolf is located even closer to the Pleistocene wolves as in Niemann et al. 2020 (Supplementary Figure 12B). In the ADMIXTURE results, the Honshu wolf shows a similar pattern to the Pleistocene wolves (Supplementary Figure 12B). When the dataset includes 7 Japanese wolves and a Honshu wolf, the Honshu wolf belongs to the same cluster as the Japanese wolf (Supplementary Figure 5). Therefore, the Honshu wolf is presumed to be closer to the Pleistocene wolf (Supplementary Figure 12B) than the single Japanese wolf data (Supplementary Figure 10A) due to its lower coverage. These results indicate that the differences between Niemann et al. 2020 and this manuscript are affected by differences in the number of individuals and the sequence coverage.

Supplementary Note 2

No gene flow between the Japanese wolf and the Pleistocene wolves.

Each Honshu wolf and Jw5k formed a monophyletic group with other Japanese wolves with a high bootstrap support (Supplementary Figure 13B and 13C), respectively, suggesting that these two individuals are a member of the Japanese wolf. *f4*-statistics suggested no gene flow between the Pleistocene wolves and the Japanese wolf (Supplementary Figure 14A) and the Honshu wolf (Supplementary Figure 14B). The phylogenetic relationship in *f4*-statistics is based on a tree in (Supplementary Figure 15). Outgroup *f3* statistics showed a high genetic affinity of PJ35k to the Pleistocene wolves (Supplementary Figure 16A). Therefore, we considered that the phylogenetic position of PJ35k is in the Pleistocene wolves clade and used this phylogenetic relationship for *f4*-statistics in (Supplementary Figure 16B), showing no gene flow between PJ35k and the Japanese wolf.

The phylogenetic analyses (Supplementary Figure 13B and 13C) did not support the hypothesis that two different origins of the Japanese wolf (Segawa et al. 2022). *f4*-statistics in (Supplementary Figure 14A, 14B, and 16B) did not support gene flow between the Japanese wolf and the Pleistocene wolves suggested by Niemann et al.2020 and Segawa et al. 2022.

Instead, we found evidence of gene flow between the Pleistocene wolves and the most recent common ancestor of the Japanese wolf and dog lineage. The Pleistocene wolves show different genetic affinities to dog/Japanese wolves (Supplementary Figure 17B), with the highest affinity of Tumat2. Therefore, we analyzed a gene flow between Tumat2 and dog/Japanese wolf individuals, and all individuals showed gene flow (Supplementary Figure 18A). However, the genetic affinities of dog/Japanese wolf individuals to the Pleistocene wolves are close to each other (Supplementary Figure 17A), suggesting gene flow between the Pleistocene wolves and the most recent common ancestor of the Japanese wolf and dog lineage (Supplementary Figure 18B).

We used a sister relationship between the Japanese wolf and the dog lineage in our analyses, but Niemann et al.2020 and Segawa et al. 2022 did not. This may cause a difference in the results of the analyses in our study and previous studies.

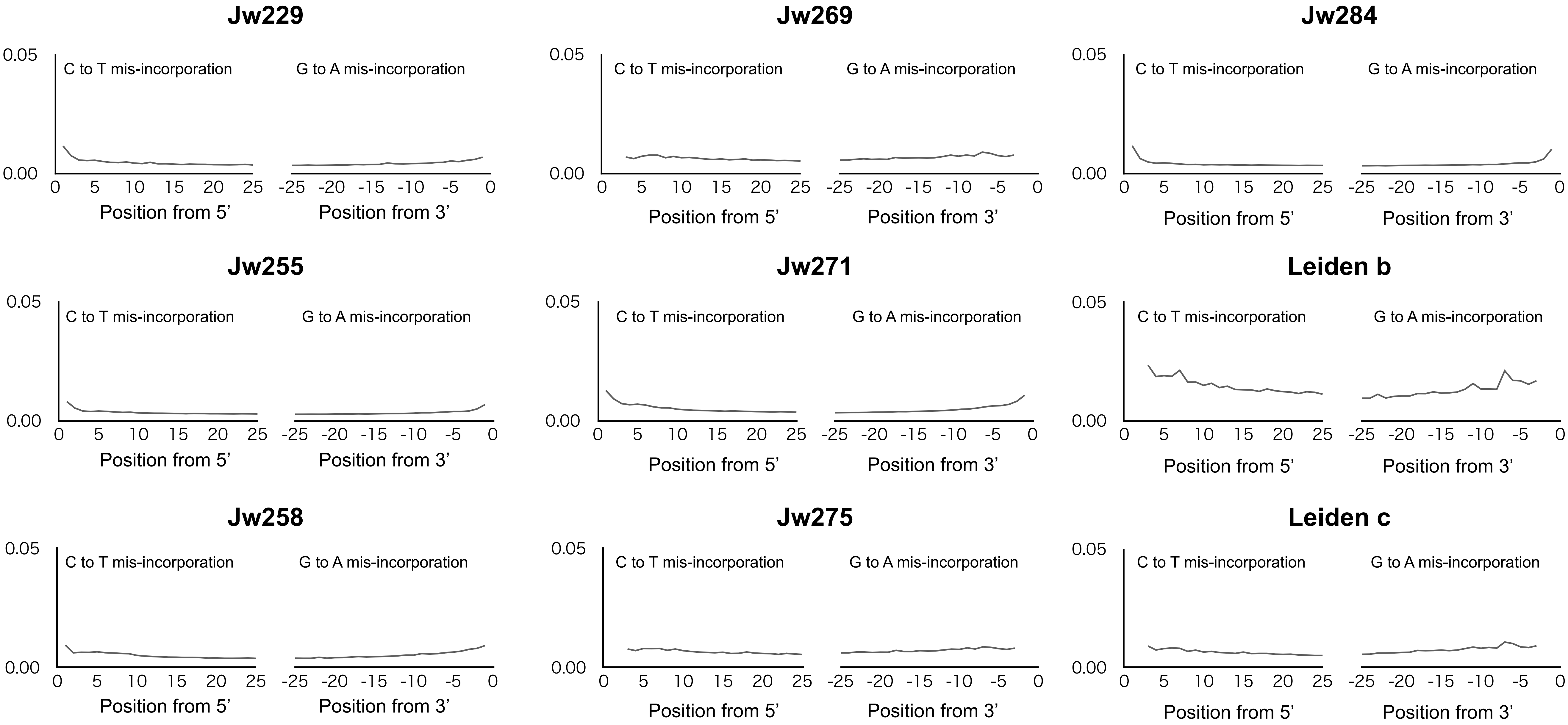

Supplementary Figure 1

C to T and G to A frequency of mis-incorporation at 3’ and 5’ end of reads determined in this study. Source data are provided as a Source Data file.

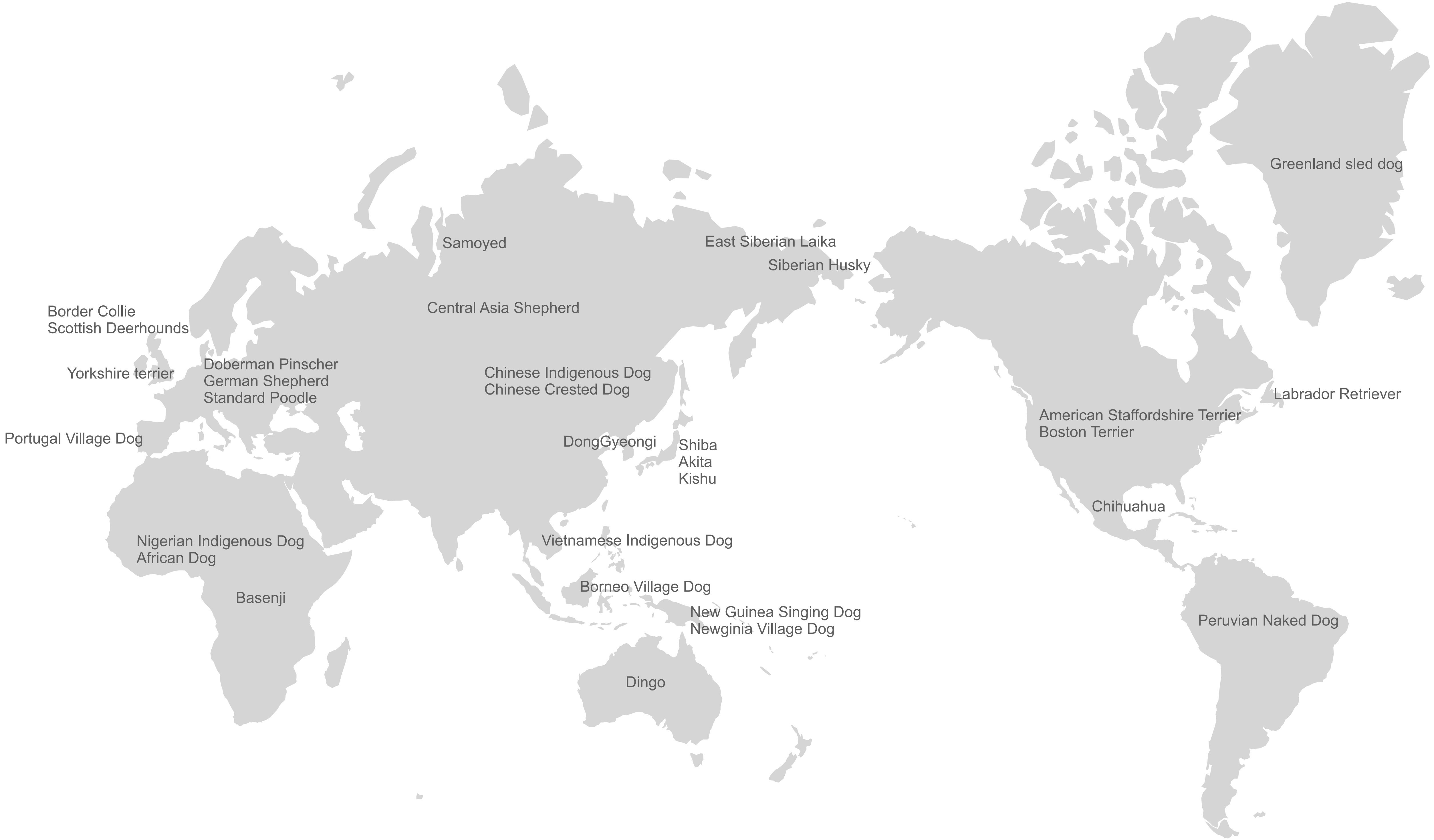

Supplementary Figure 2

Maps with the location of dog breeds.

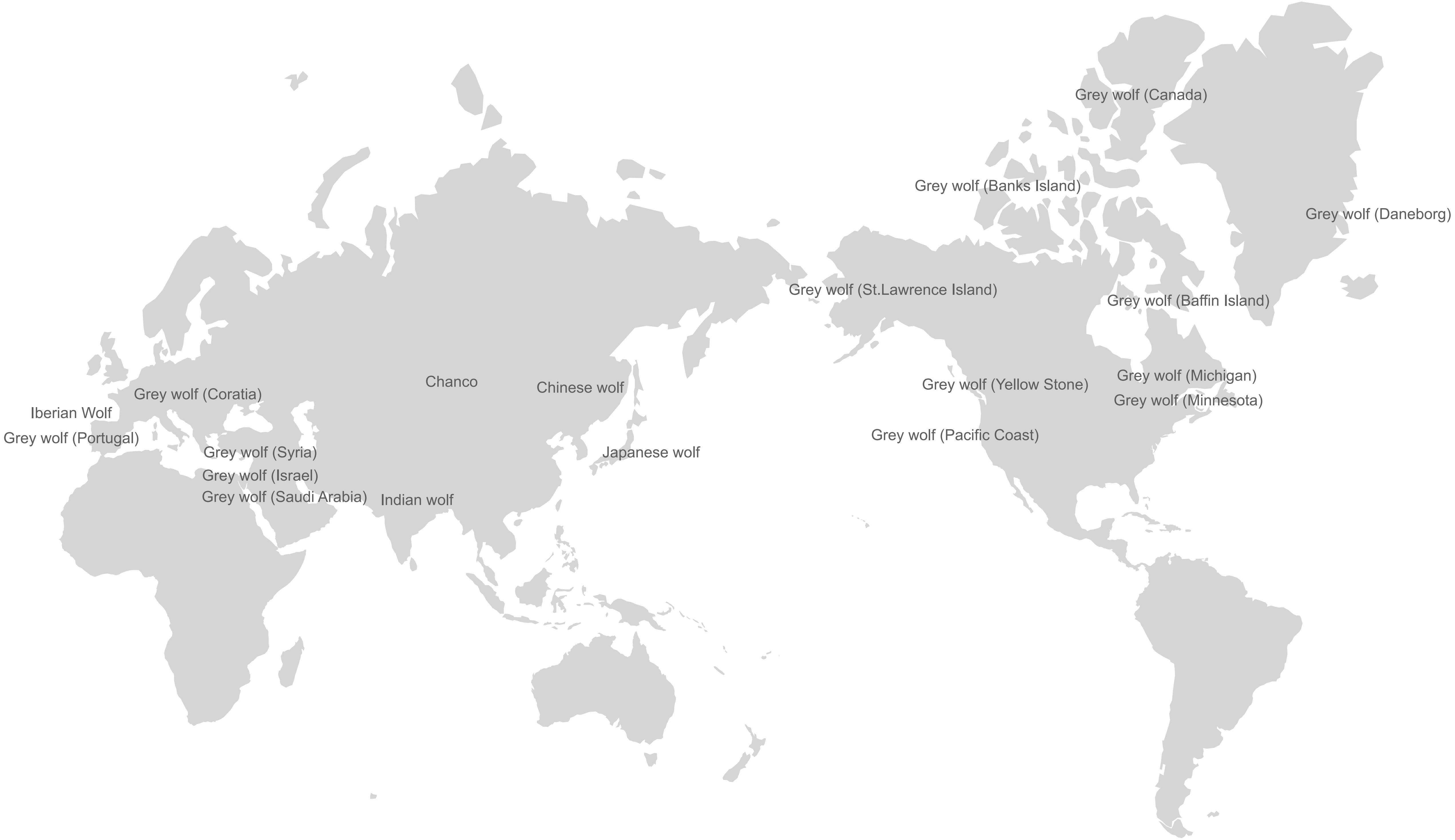

Supplementary Figure 3
Maps with the location of gray wolf populations.

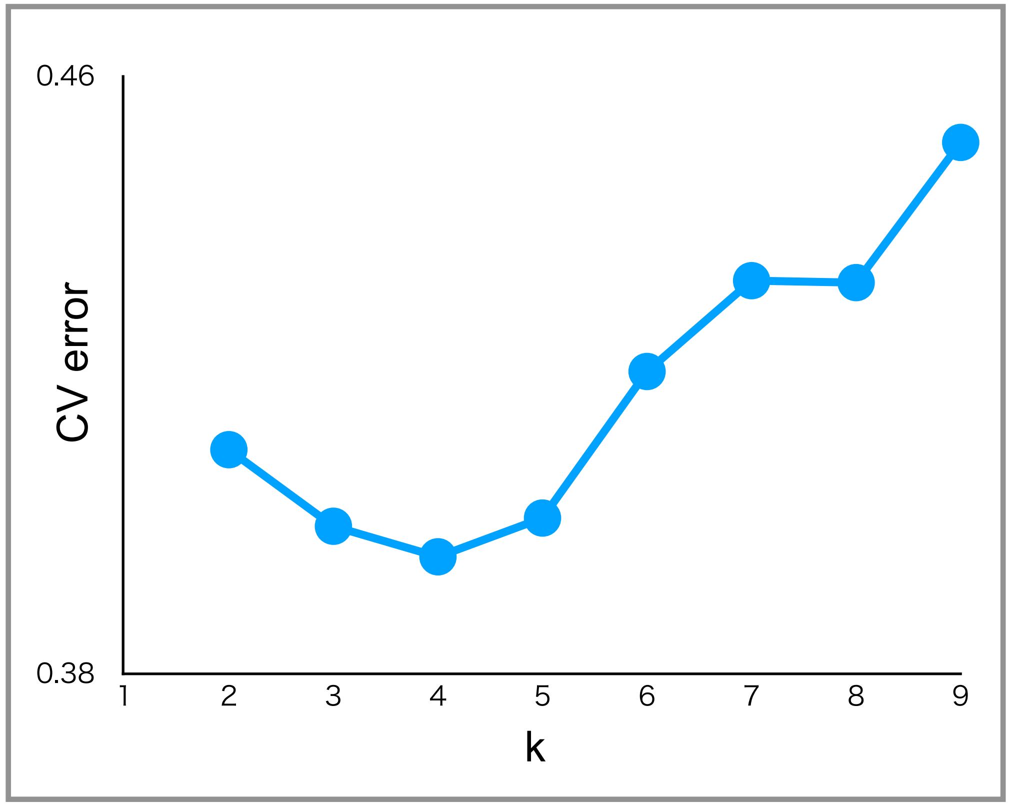

Supplementary Figure 4

Cross validation (CV) values for ADMIXTURE analysis (Supplementary Figure 5) based on 290,414 unlinked biallelic SNPs extracted from 1,696,115 sites. Source data are provided as a Source Data file.

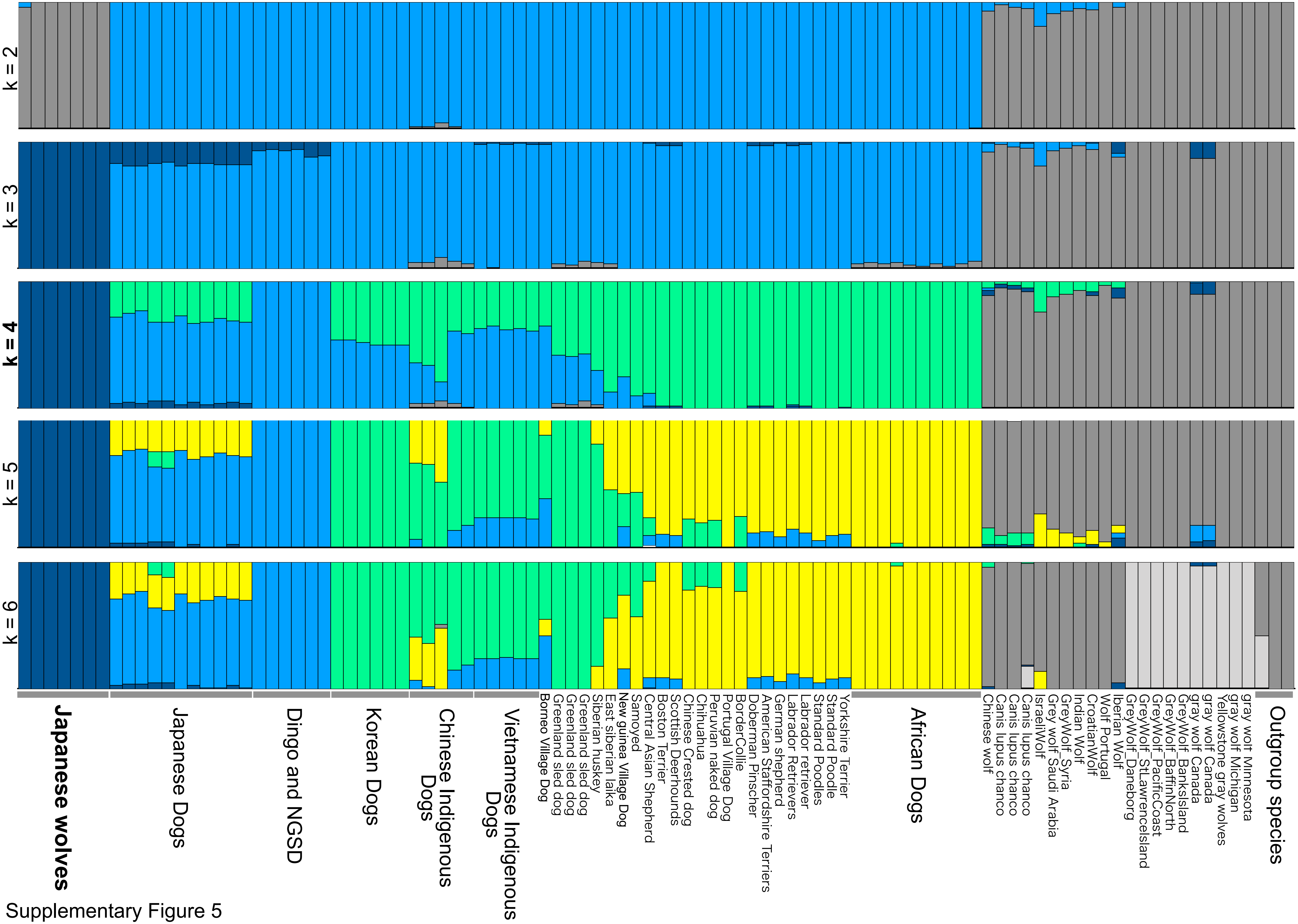

Supplementary Figure 5

ADMIXTURE results based on 290,414 unlinked biallelic SNPs extracted from 1,696,115 sites for K = 2-6 (see Supplementary Data 2 for sample information). Source data are provided as a Source Data file.

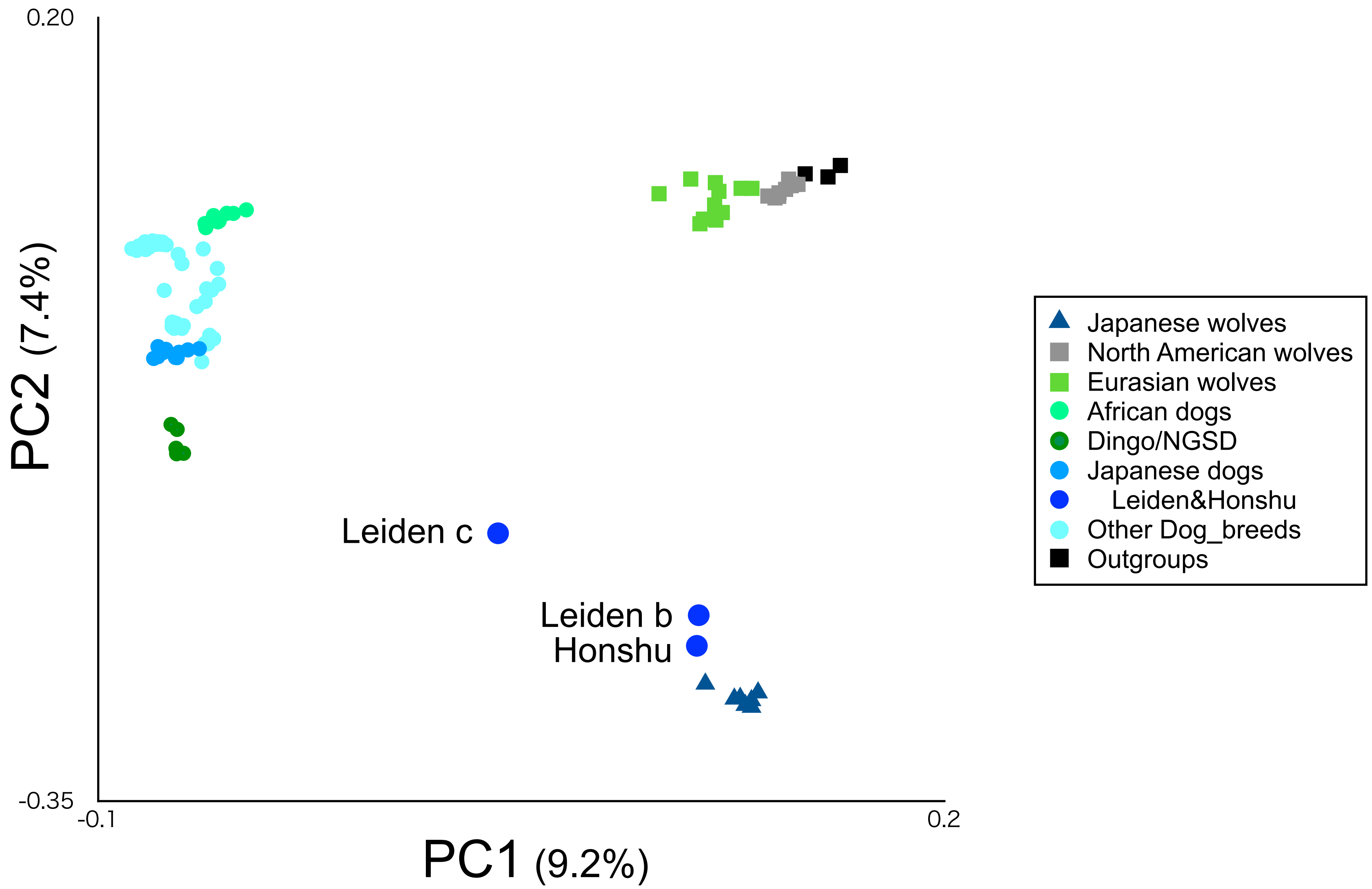

Supplementary Figure 6

Principal Components Analysis (PC1 versus PC2) of 102 samples based on 55,300 unlinked biallelic SNPs extracted from 342,931 sites (see Supplementary Data 2 for sample information). Colored circle, square, and triangle correspond to the names of dogs or wolves in the panel. Source data are provided as a Source Data file.

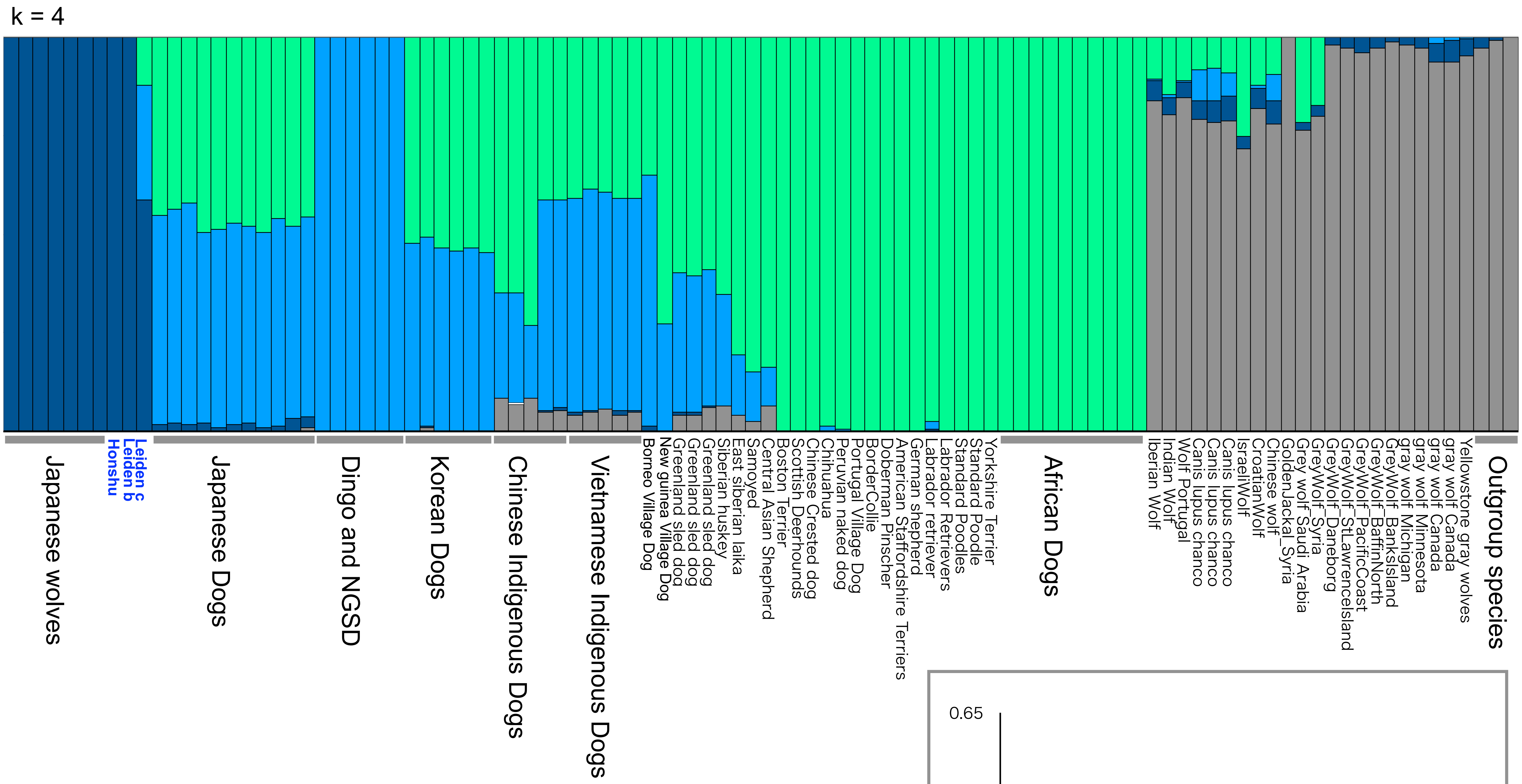

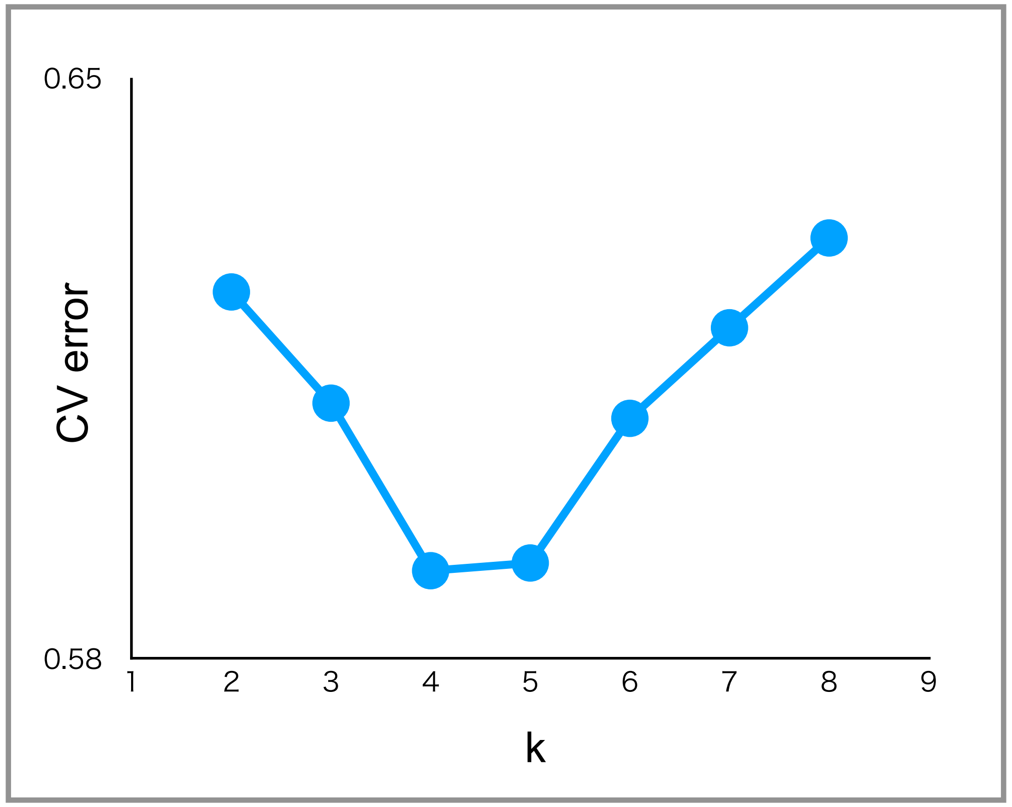

Supplementary Figure 7

An ADMIXTURE result based on 55,300 unlinked biallelic SNPs extracted from 342,931 sites for K = 4 (see Supplementary Data 2 for sample information). Cross validation (CV) values for ADMIXTURE analysis of SNP data is shown in the panel. Source data are provided as a Source Data file.

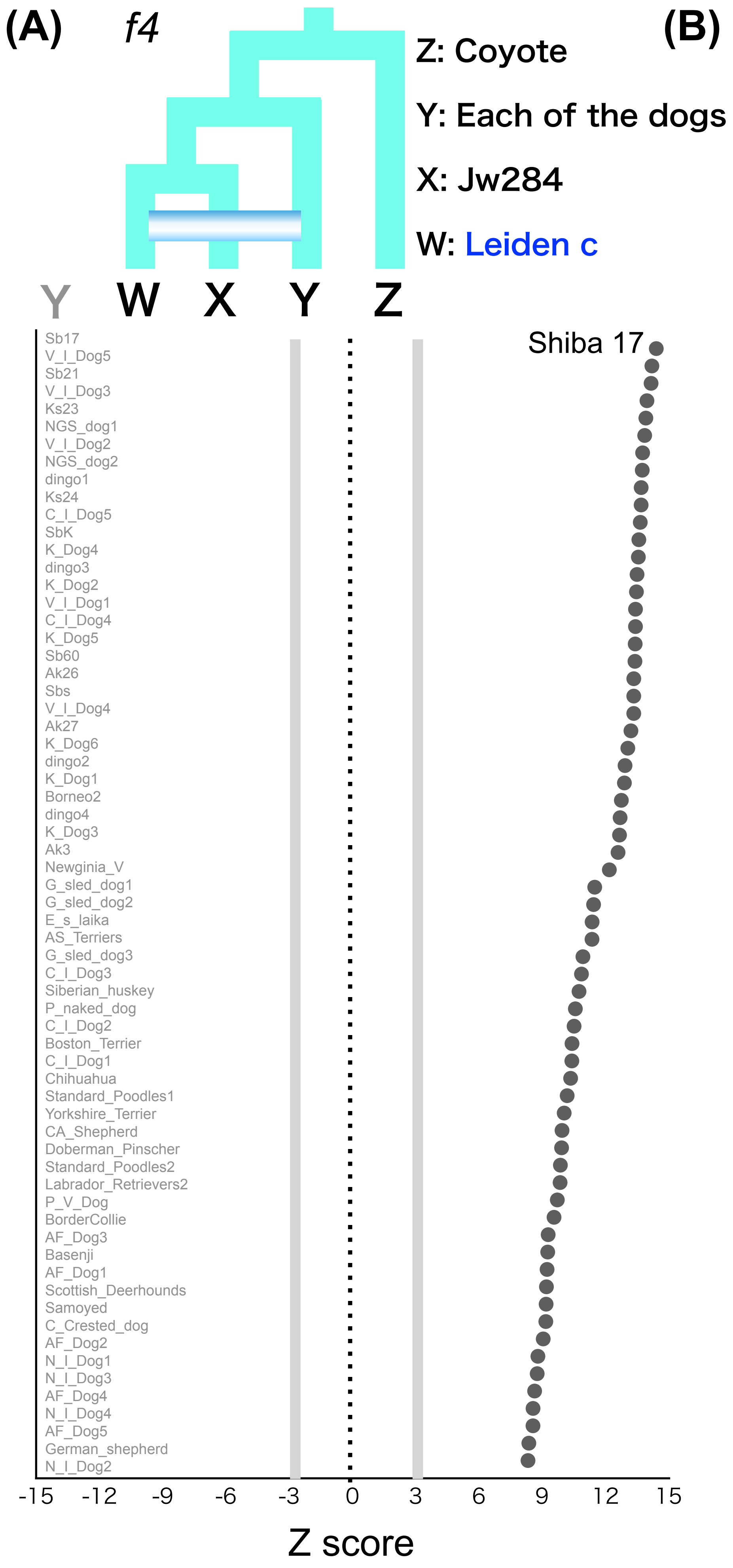

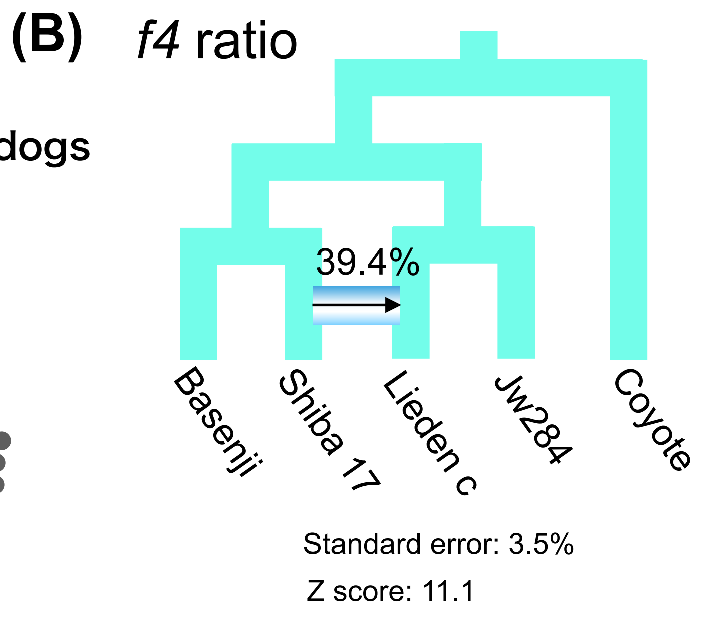

Supplementary Figure 8

(A) *f4*-statistics testing the relationships between the Japanese wolf (Jw284), Leiden c, and all other dogs. Each Z score is plotted in order of highest to lowest value from the top, and the names of dogs are shown on the left side of each panel. Gray lines show the Z score -3 and 3. Leiden c shows genetic affinity with all other dogs (Z score > 3, n = 4). (B) *f4*-ratio test to estimate proportion of genome introgression from a Japanese dog (Shiba 17) to Leiden C. (C) *f4*-statistics testing the relationships between the Japanese wolf (Jw284), Leiden b, and all other dogs. The genetic affinities of Leiden b with all other dogs are rejected (-3 < Z score < 3, n = 4). The estimated contamination rate of Leiden c (0.7%, Supplementary Data 5) rejected a possibility of contamination of dog DNA. Source data are provided as a Source Data file.

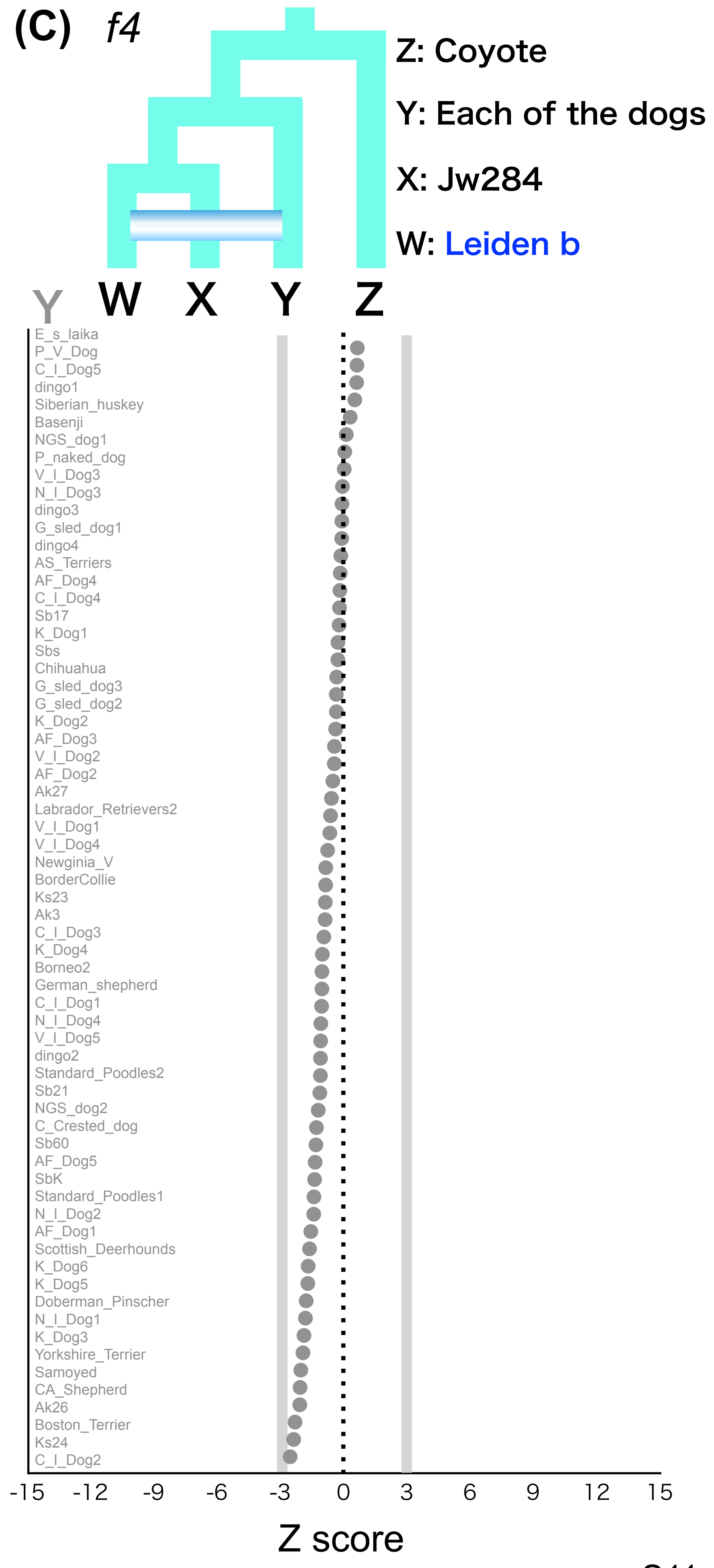

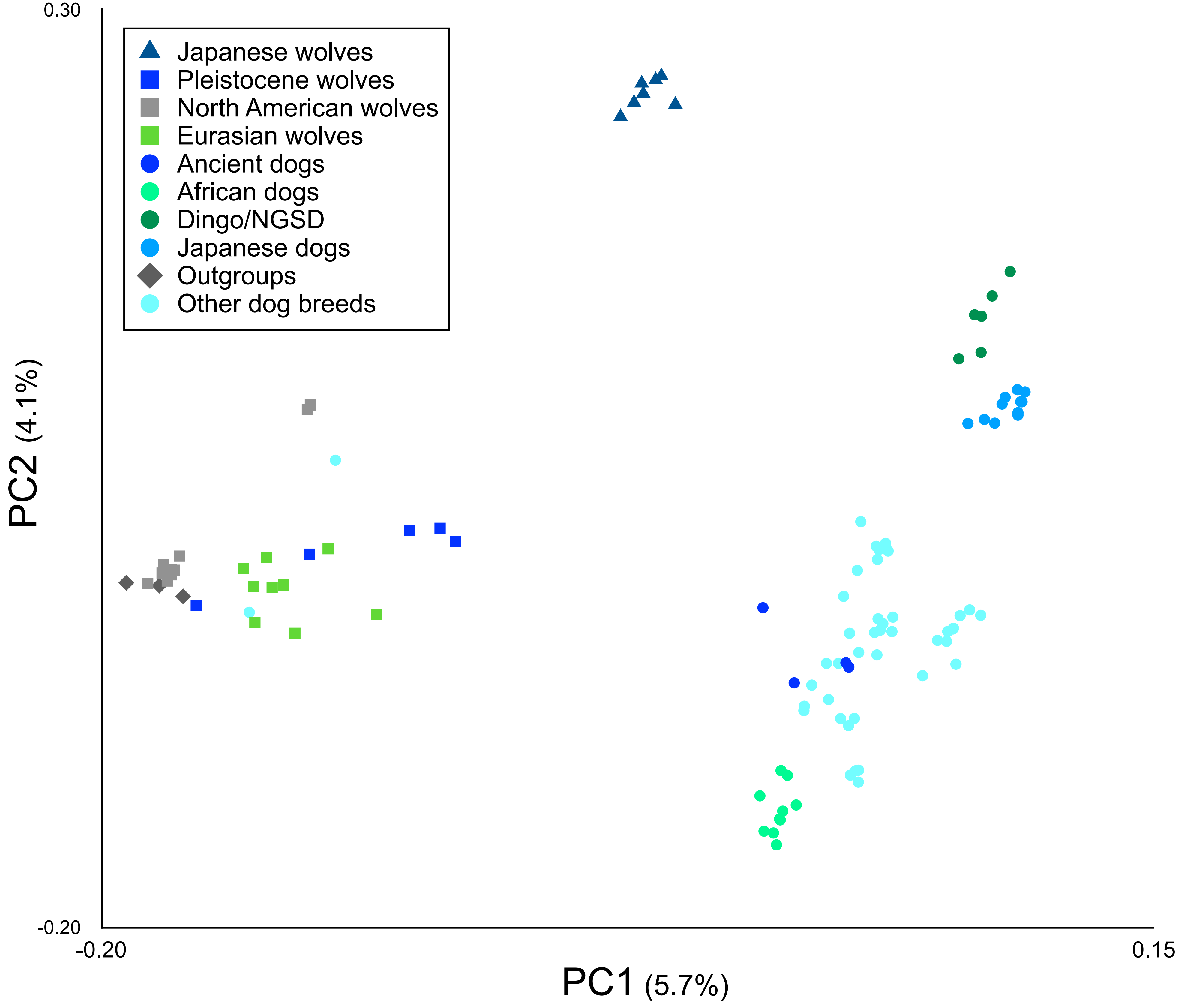

Supplementary Figure 9
Principal Components Analysis (PC1 versus PC2) of 109 samples based on 39,895 unlinked biallelic SNPs extracted from 100,588 sites(transversion sites, see Supplementary Data 2 for sample information). Colored circle, square, and triangle correspond to the names of dogs or wolves in the panel. Source data are provided as a Source Data file.

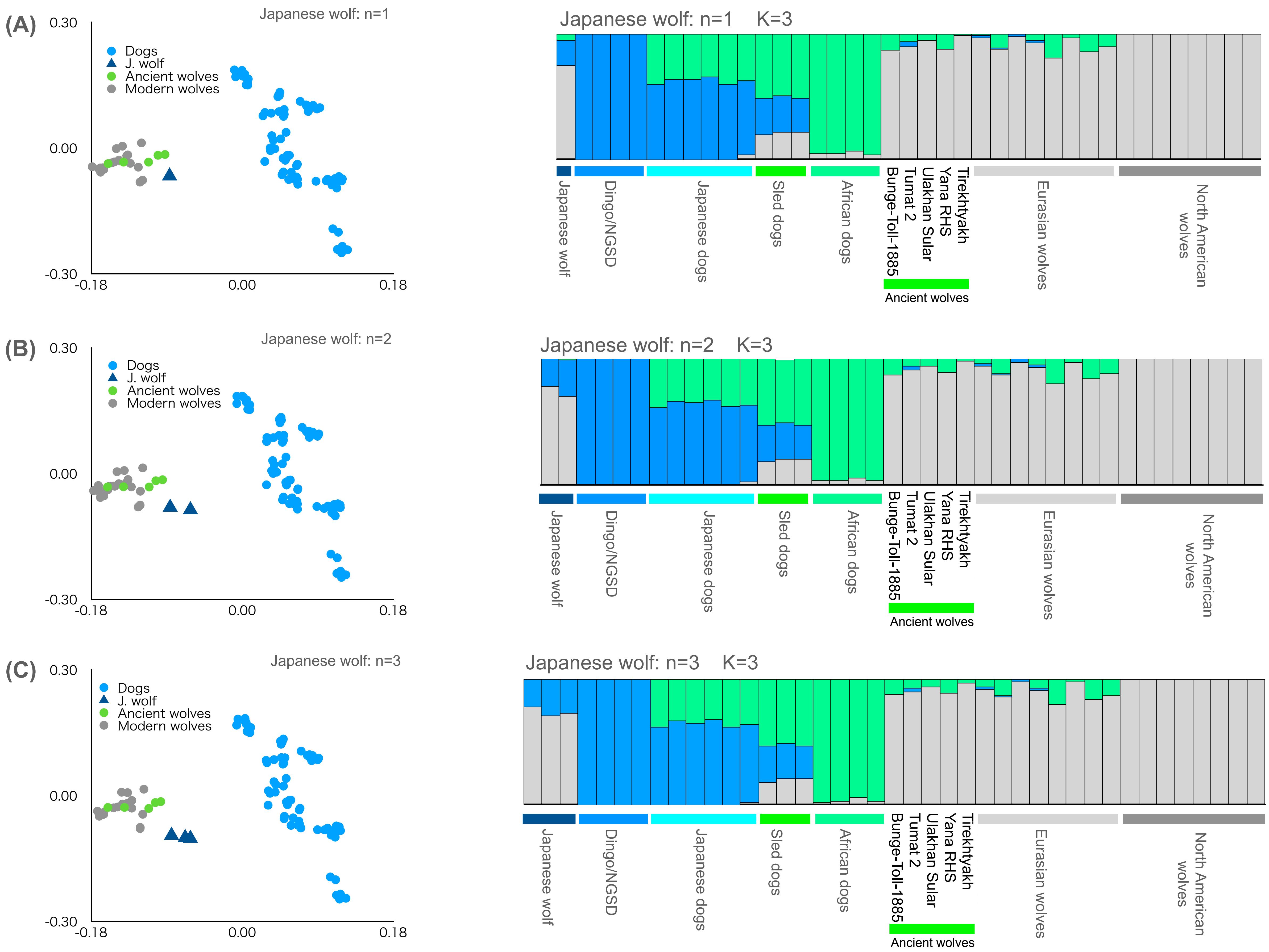

Supplementary Figure 10

Principal Components Analysis (PC1 versus PC2) and an ADMIXTURE result based on unlinked biallelic SNPs (only transversion sites) from dogs, modern wolves, ancient wolves, and Japanese wolf individuals ranging from 1 (A) to 3 (C). Only the ADMIXTURE results with the lowest Cross validation (CV) values were shown. Source data are provided as a Source Data file.

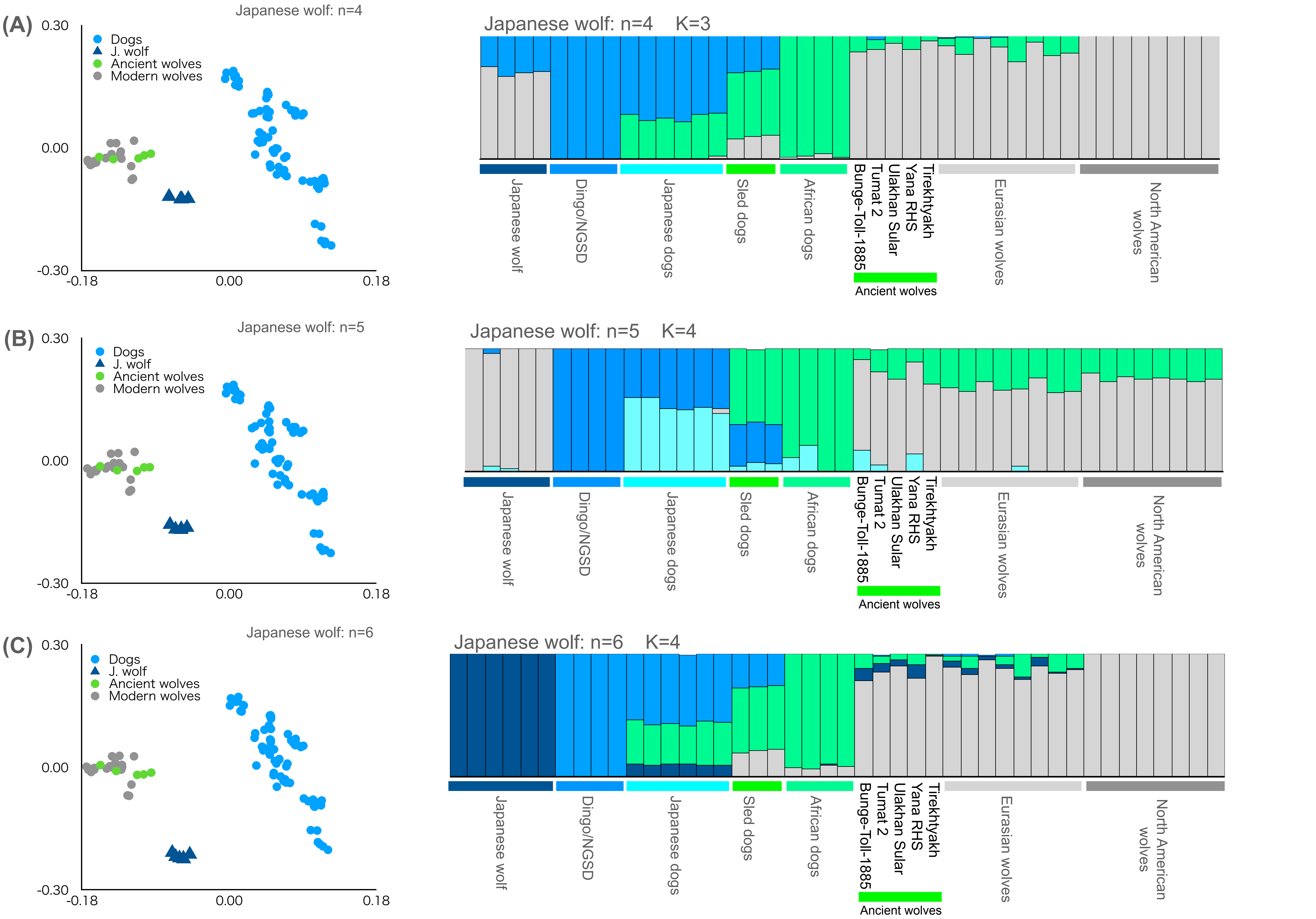

Supplementary Figure 11

Principal Components Analysis (PC1 versus PC2) and an ADMIXTURE result based on unlinked biallelic SNPs (only transversion sites) from dogs, modern wolves, ancient wolves, and Japanese wolf individuals ranging from 4 (A) to 6 (C). Only the ADMIXTURE results with the lowest Cross validation (CV) values were shown. Source data are provided as a Source Data file.

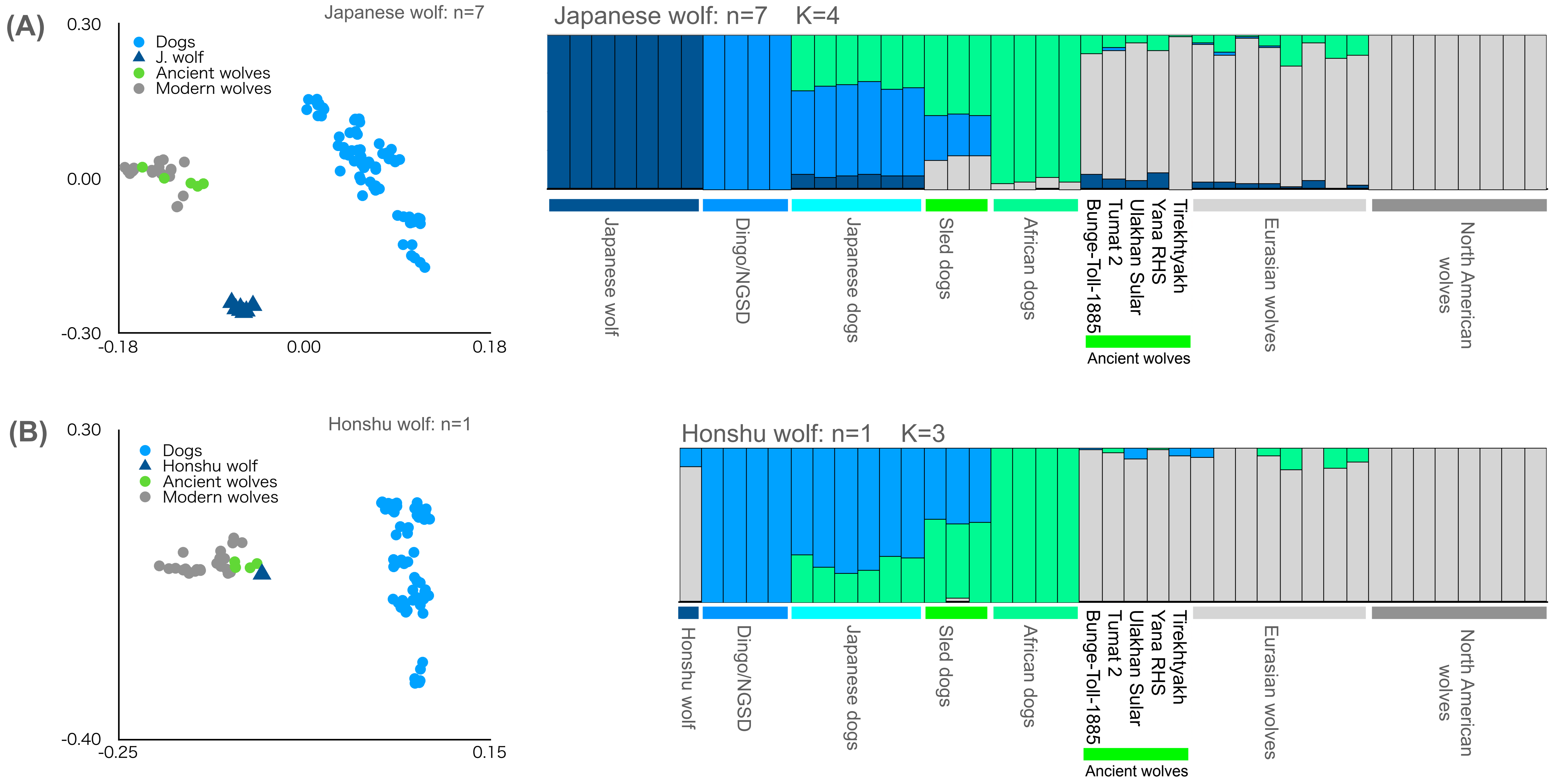

Number of SNPs site after filtering

Japanese wolves	Number of SNPs site
n=1	47882
n=2	48175
n=3	48483
n=4	48831
n=5	49224
n=6	49742
n=7	50036
Honshu wolf n=1	23473

Supplementary Figure 12

Principal Components Analysis (PC1 versus PC2) and an ADMIXTURE result based on unlinked biallelic SNPs (only transversion sites) from dogs, modern wolves, ancient wolves, and seven Japanese wolf individuals (A) or a Honshu wolf individual (B). Only the ADMIXTURE results with the lowest Cross validation (CV) values were shown. The numbers of SNPs sites used for Supplementary Figure 10-12 are shown in the table. Source data are provided as a Source Data file.

(A)

Study	ID	Period	Average coverage at SNP site*	Average coverage**	Reference covered
**Niemann et al. 2020**					
ERS5374233	Honshu wolf	Meiji		1.3x	62%
**Segawa et al. 2022**					
PRJDB10477	Jw5k	5,000 ya	2.4x*	0.02x	1%
PRJDB10477	PJ35k	35,000 ya	1.8x*	0.4x	2%

*In Segawa et al. 2022, the average depth of coverage of the nuclear genome was calculated by averaging the read coverage of the SNP sites, indicating average coverage of only mapped positions.

**The average coverage was calculated by (total length of mapped reads) / (genome size).

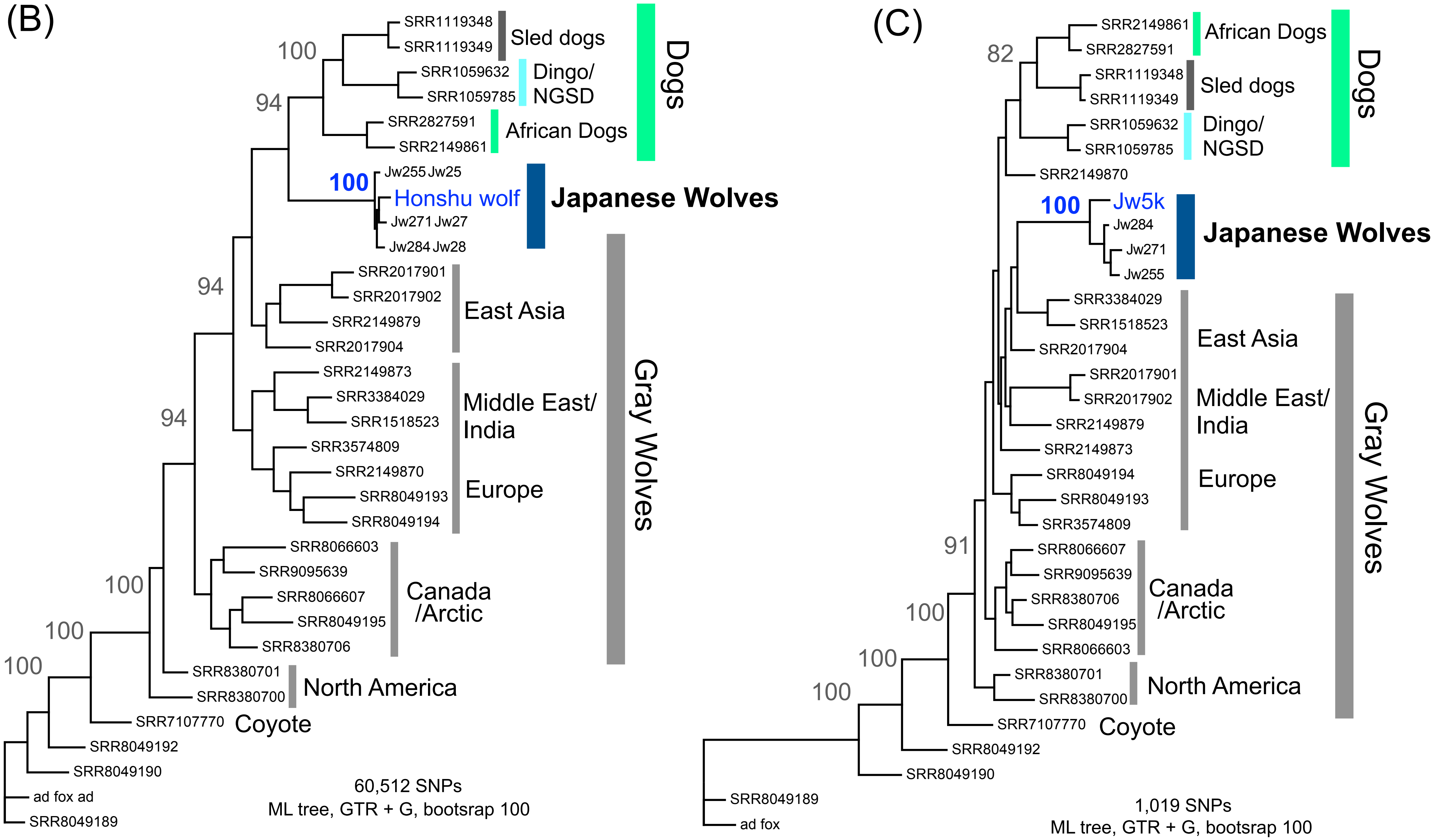

Supplementary Figure 13

(A) Summary of the sequencing data of the Honshu wolf, a 5,000 years-old Japanese wolf (Jw5k), and a 35,000 years-old Pleistocene wolf (PJ35k) from Japan. Maximum likelihood trees based on (B) 60,512, and (C) 1019 SNPs. Node labels indicate bootstrap replicates (> 80).

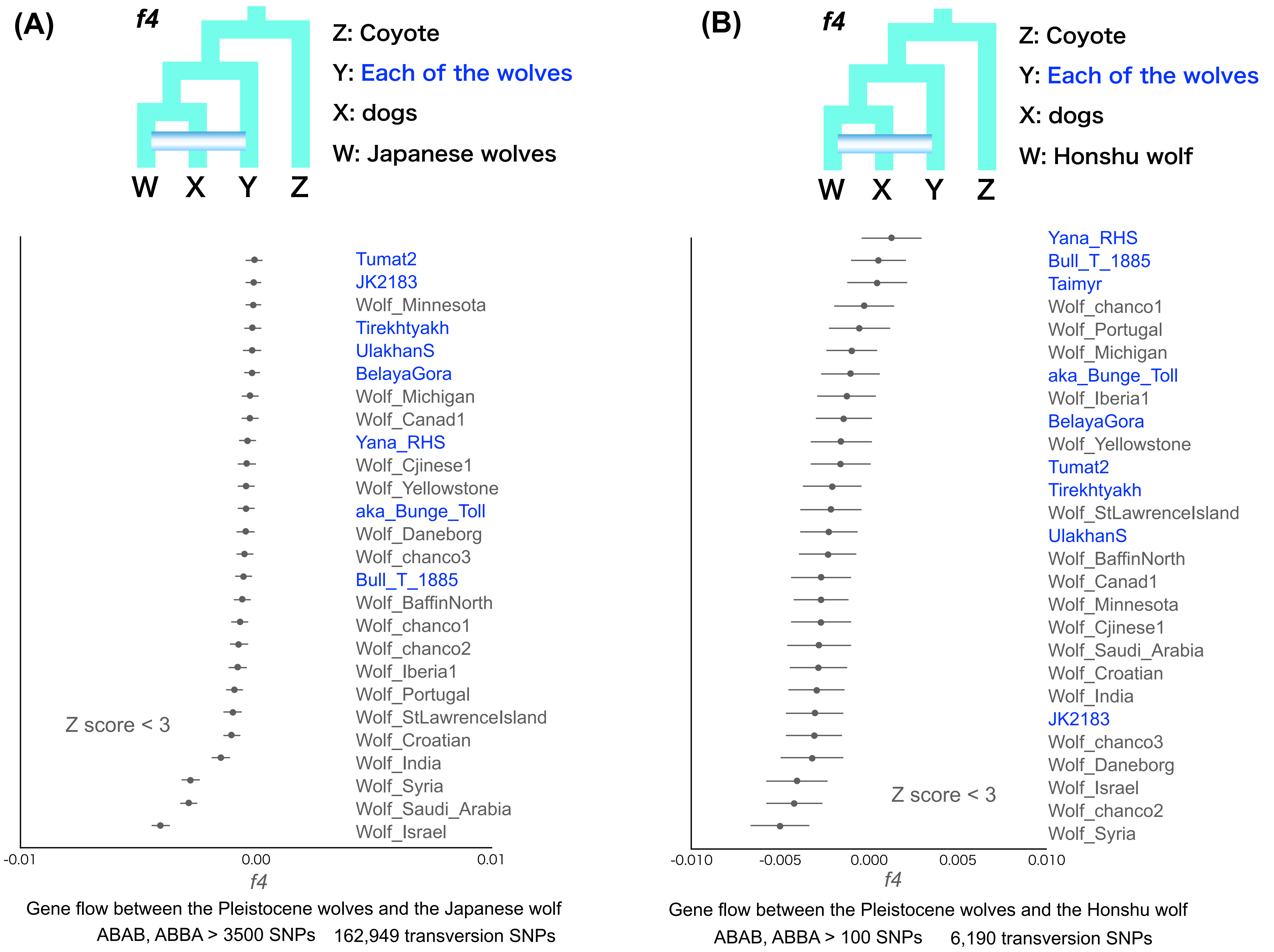

Supplementary Figure14

f4-statistics testing the relationships between the Pleistocene wolves and the Japanese wolf (A), the Pleistocene wolves and the Honshu wolf (B). Source data are provided as a Source Data file. Error bars represent standard errors (n = 6 for A, n = 9 for B)

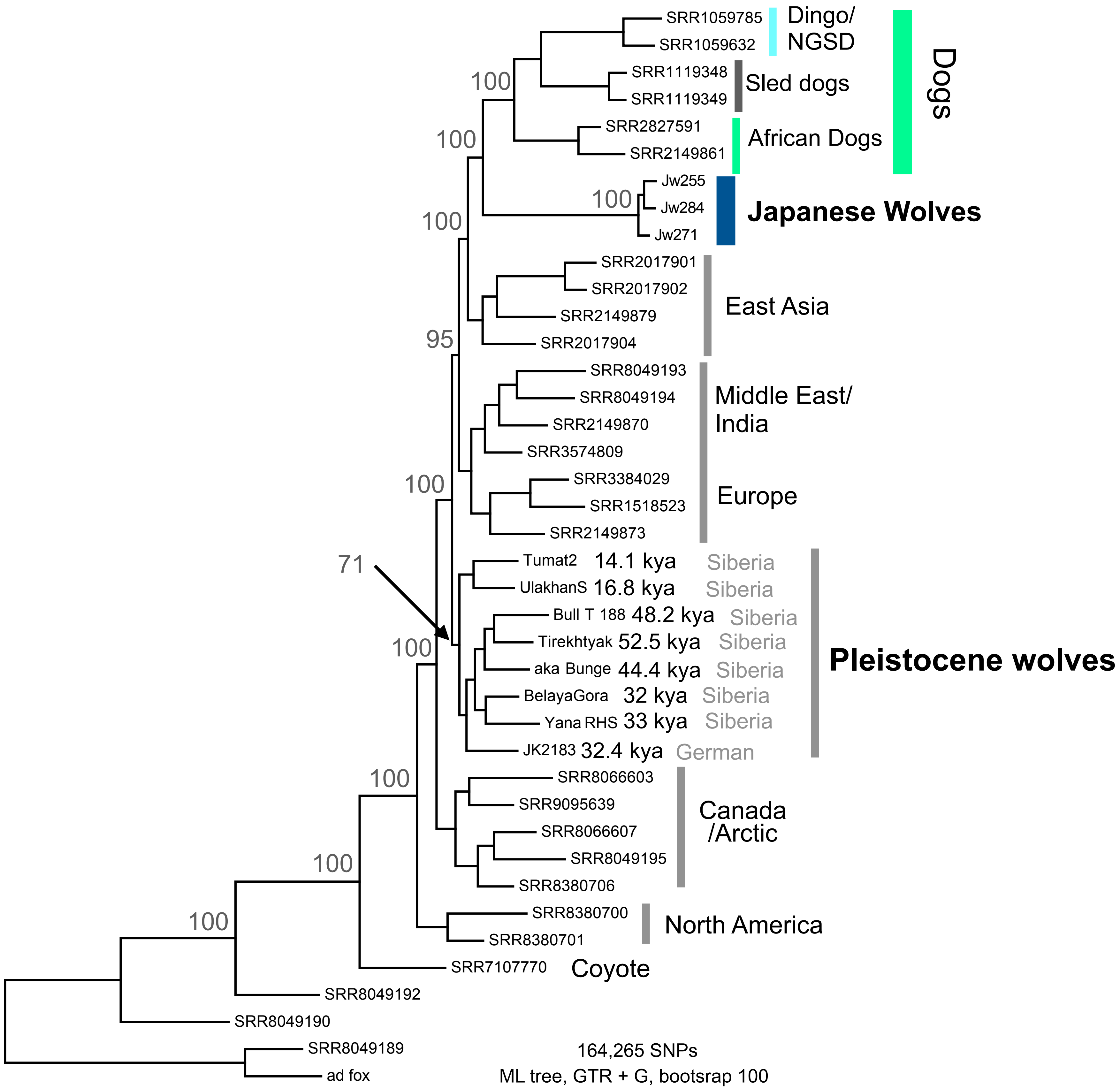

Supplementary Figure 15
A maximum likelihood tree based on 164,265 SNPs.

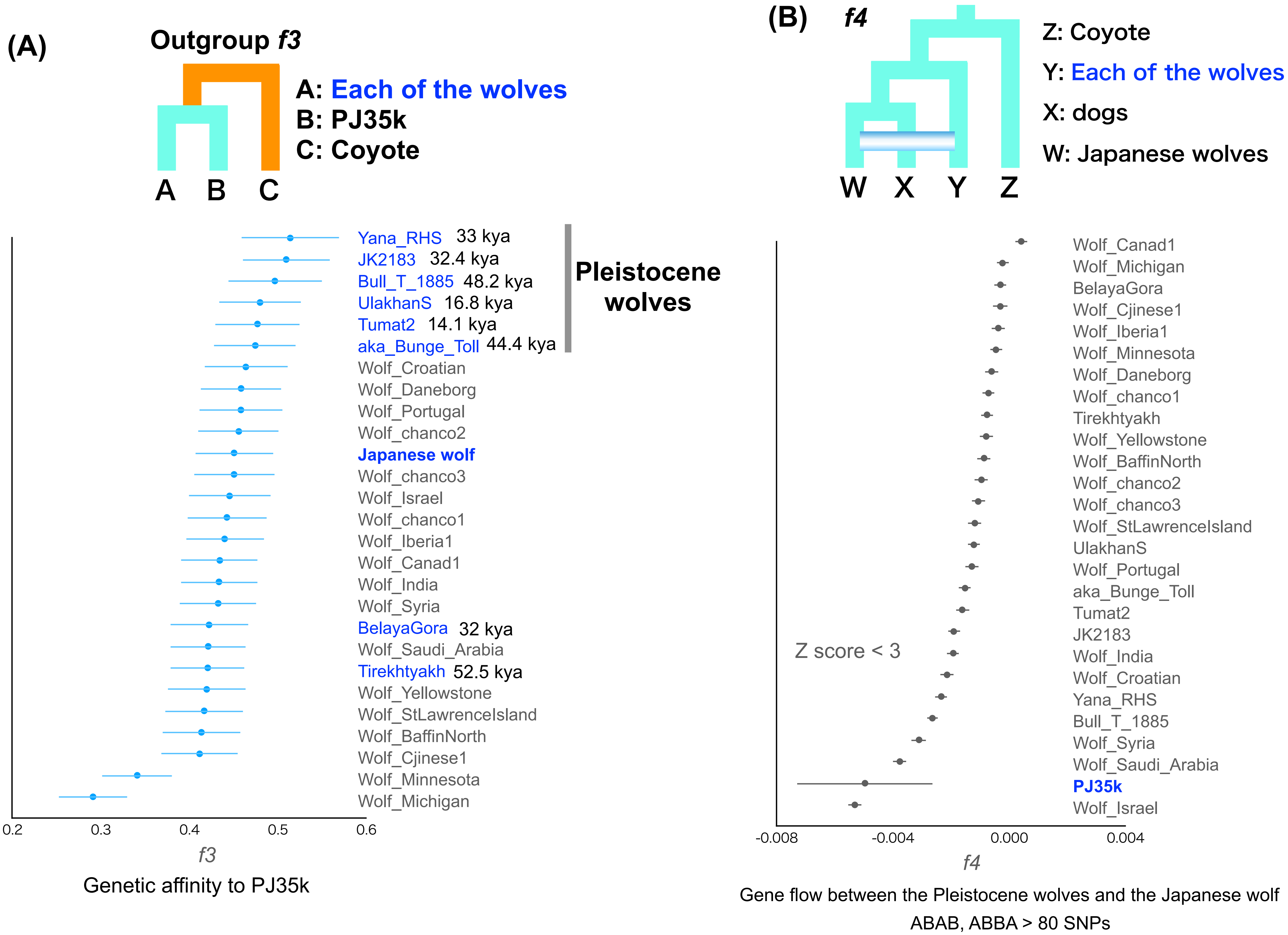

Supplementary Figure 16

Shared genetic drift between PJ35k and all wolves (A) measured by outgroup *f3* statistics. *f4*-statistics testing the relationships between the Pleistocene wolves including PJ35k and the Japanese wolf (B). Source data are provided as a Source Data file. Error bars represent standard errors (n = 3 for A, n = 11 for B)

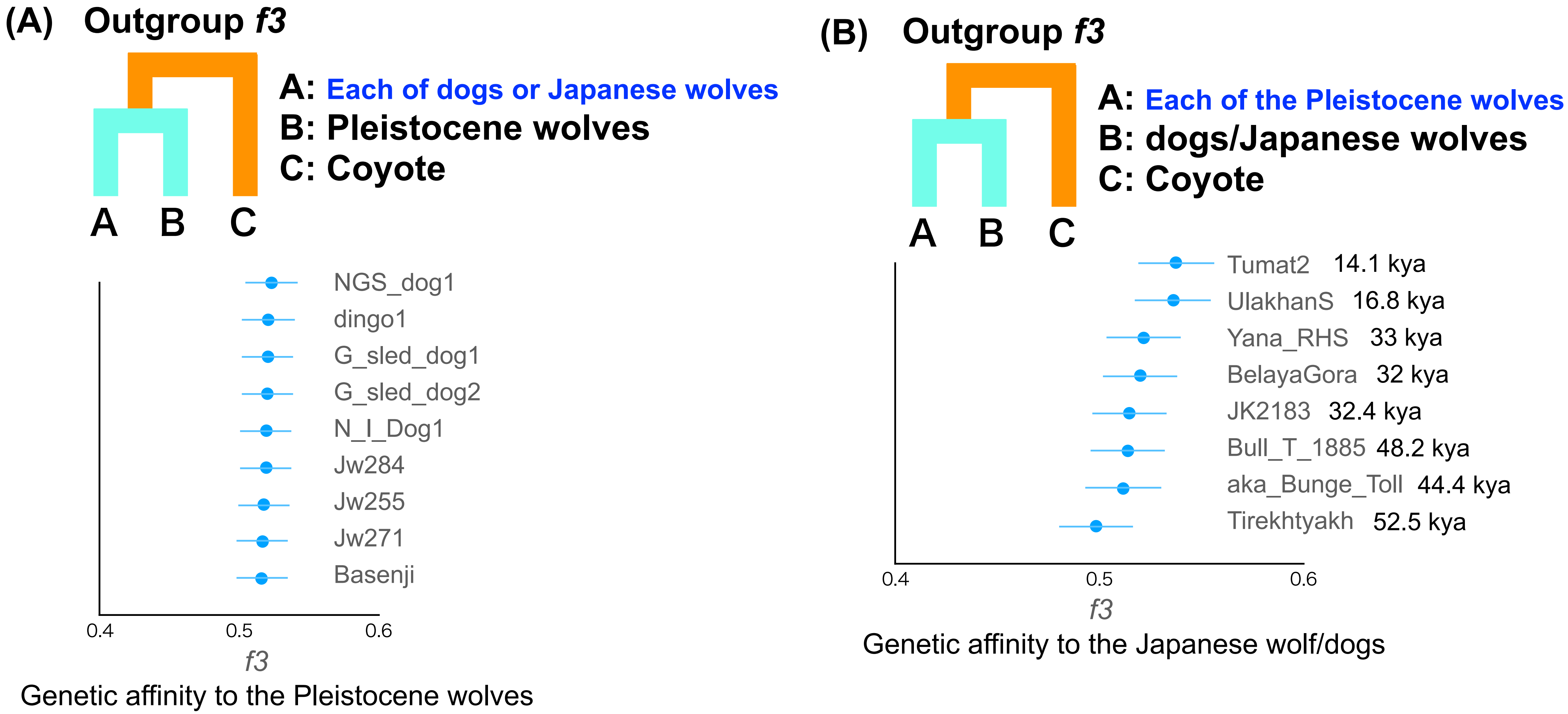

Supplementary Figure 17
Shared genetic drift between the Pleistocene wolves (grouped) and dogs/the Japanese wolves (A), and dog/Japanese wolves (grouped) and the Pleistocene wolves (B) measured by outgroup *f3* statistics. Source data are provided as a Source Data file. Error bars represent standard errors (n = 10 for A, n = 18 for B)

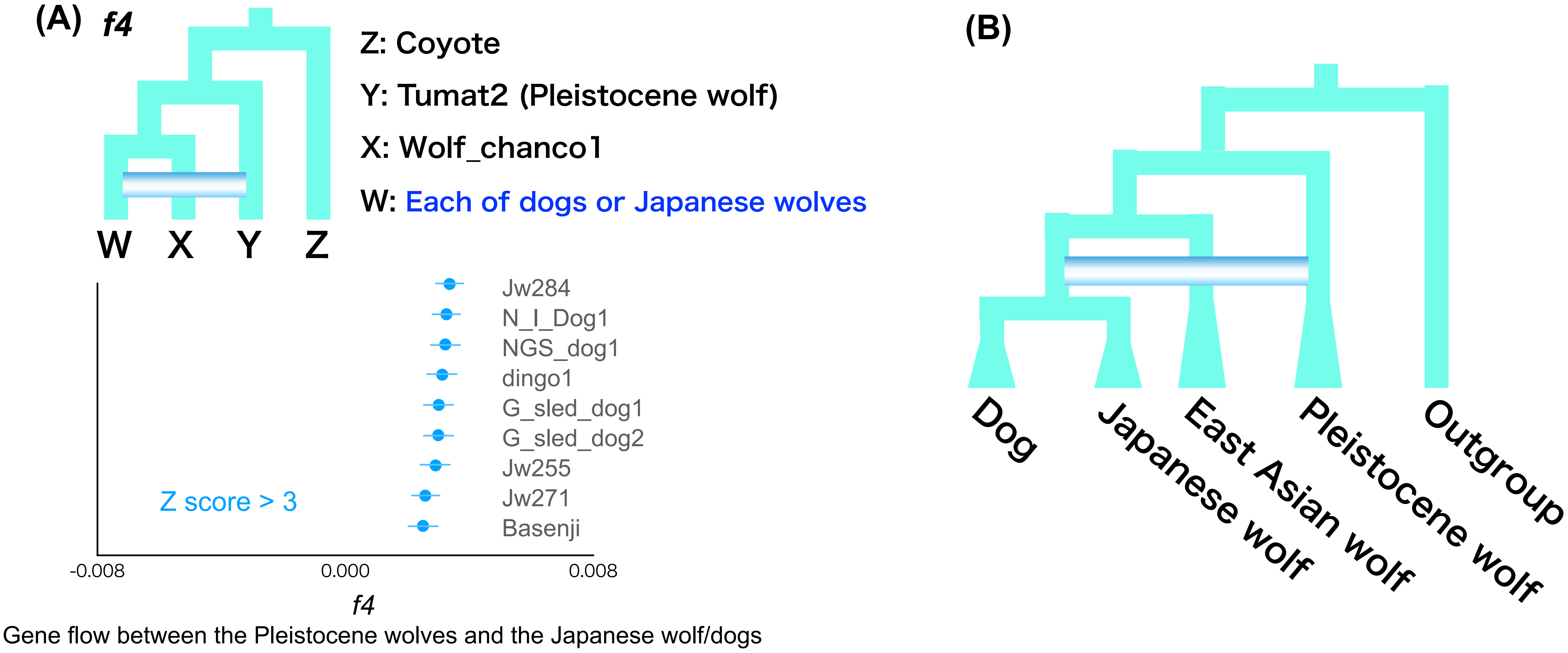

Supplementary Figure 18

f4-statistics testing the relationships between Tumat2 (Pleistocene wolf) and dogs/Japanese wolves (A) Error bars represent standard errors (n = 4) . (B) a model of gene flow between the most recent common ancestor of the dog/Japanese wolf and the Pleistocene wolf. A dot with a Z score > 3 is colored in light blue. Source data are provided as a Source Data file.

Supplementary Figure 19
Maximum likelihood tree based on 327,402 unlinked biallelic SNPs extracted from 2,065,002 sites. Node labels indicate bootstrap replicates.

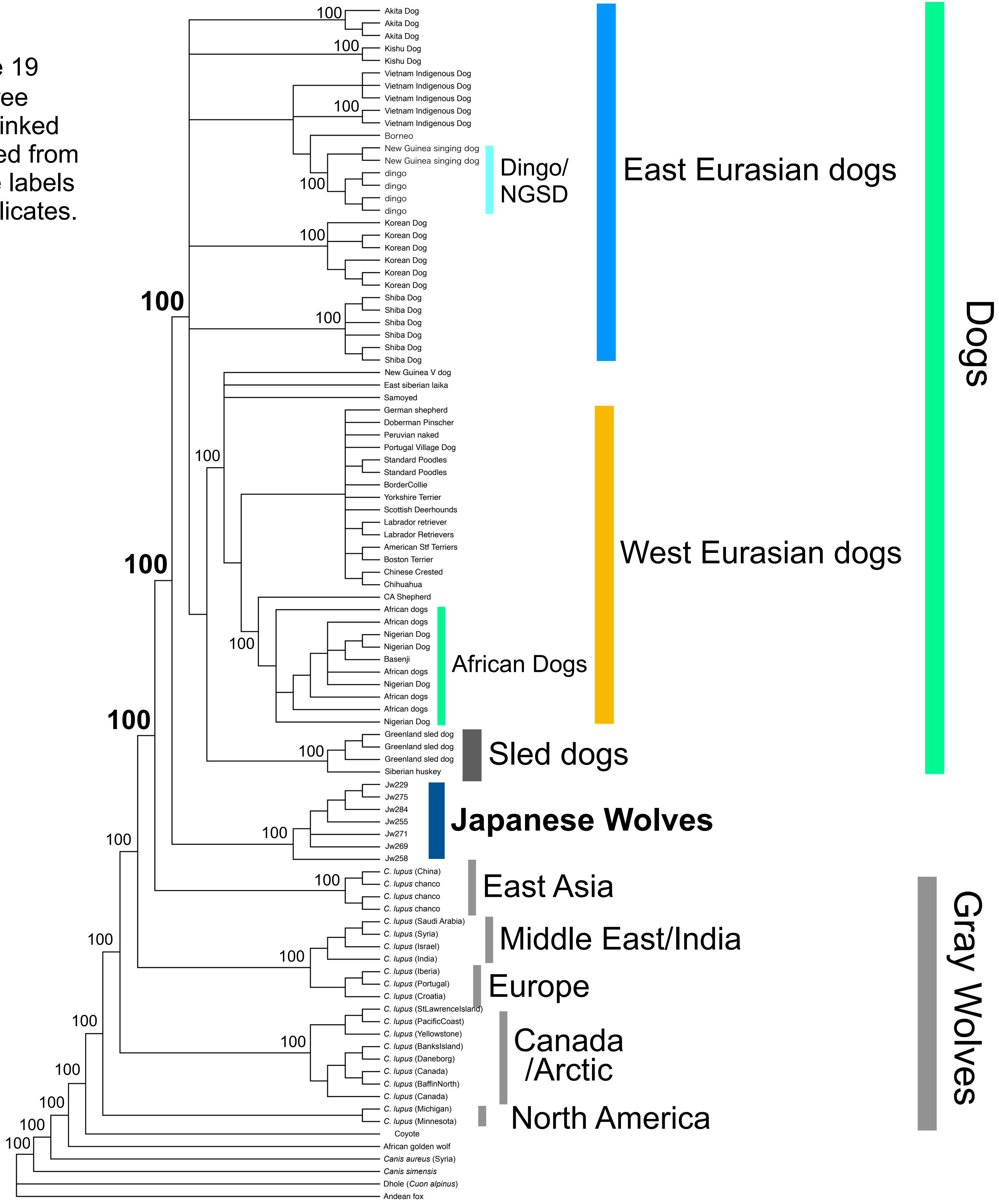

Supplementary Figure 20
Phylogenetic tree constructed by SVDquartets based on 327,402 unlinked biallelic SNPs extracted from 2,065,002 sites. Node labels indicate bootstrap replicates.

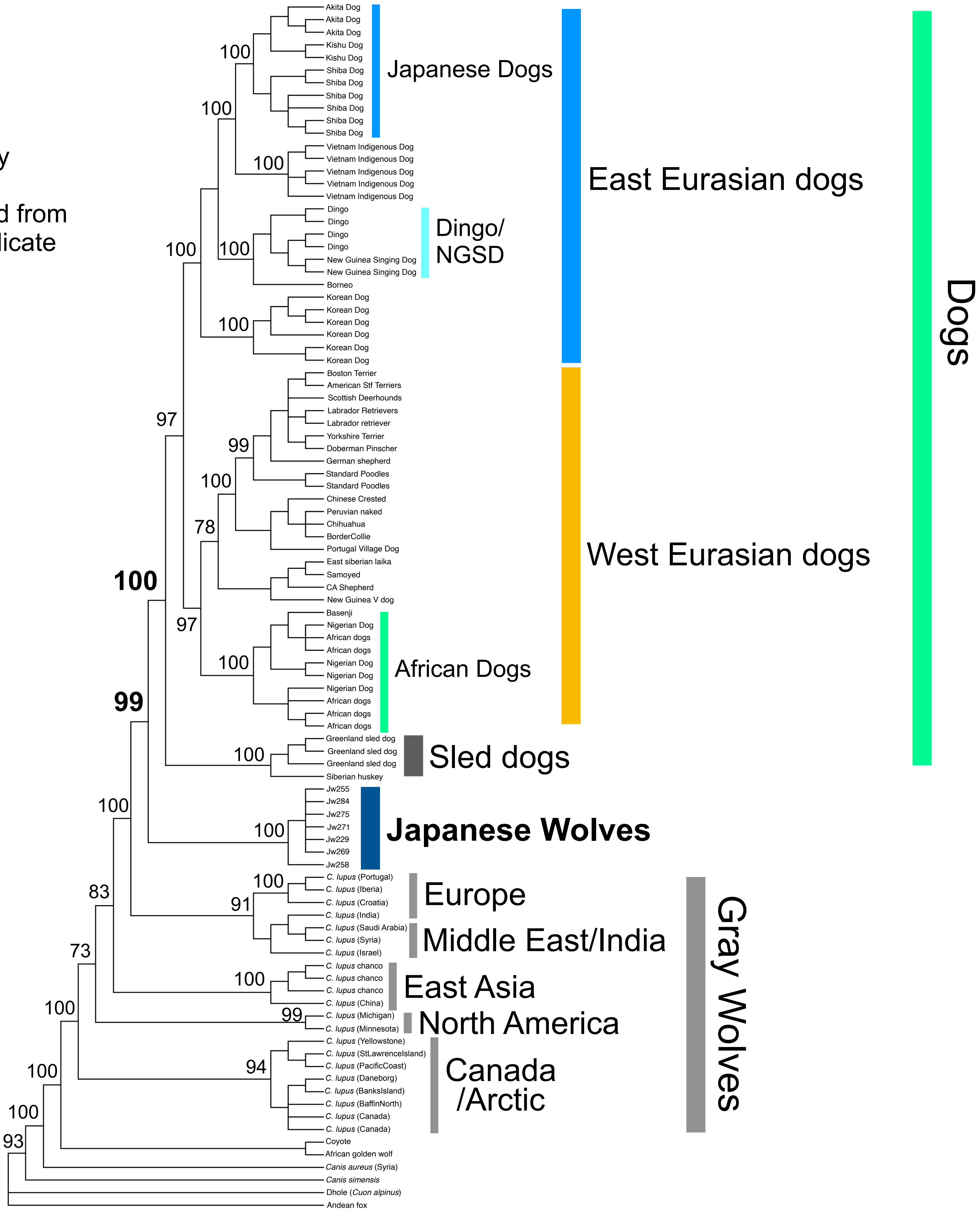

Supplementary Figure 21
NJ tree based on IBS matrix from 327,402 unlinked biallelic SNPs extracted from 2,065,002 sites.

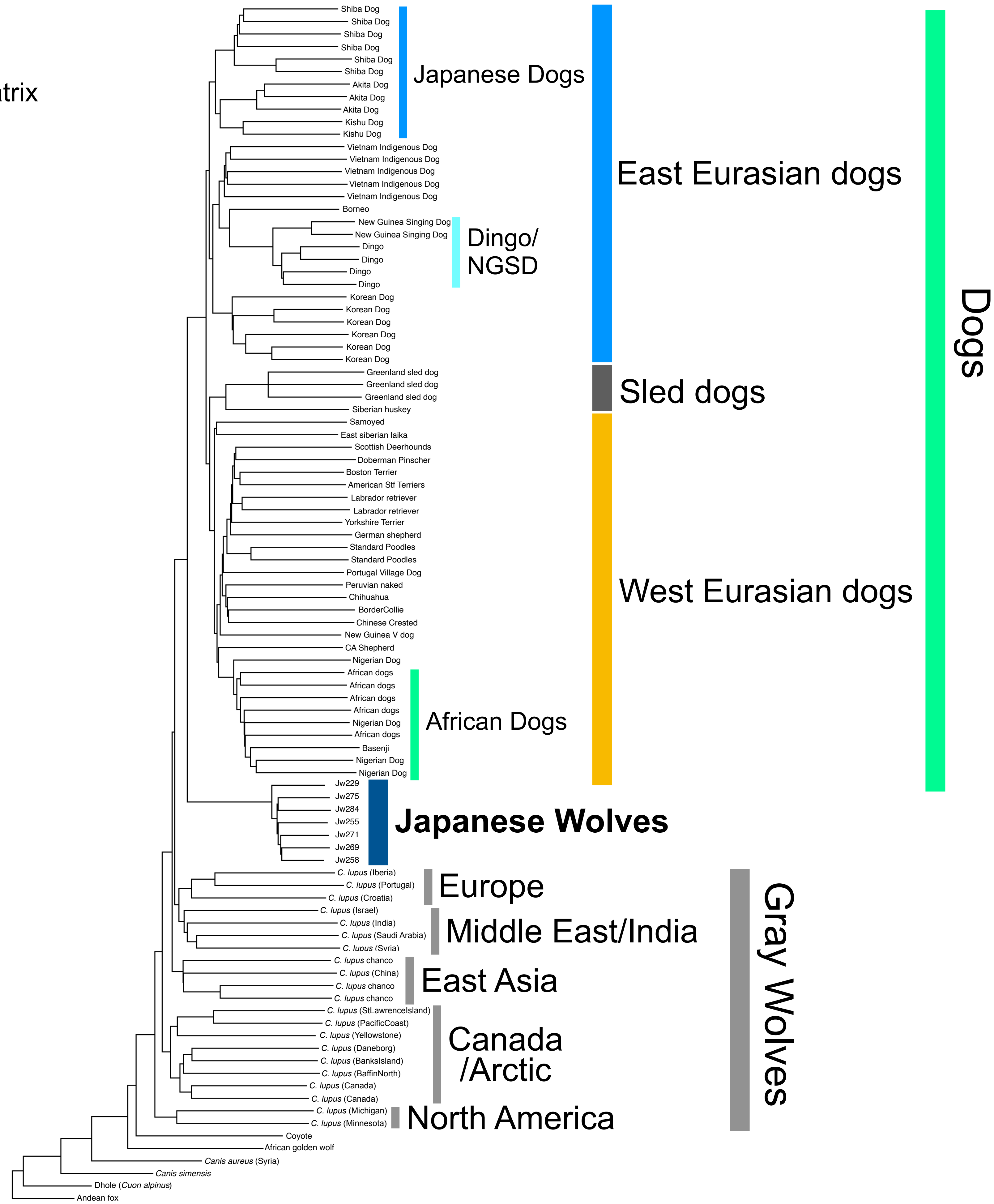

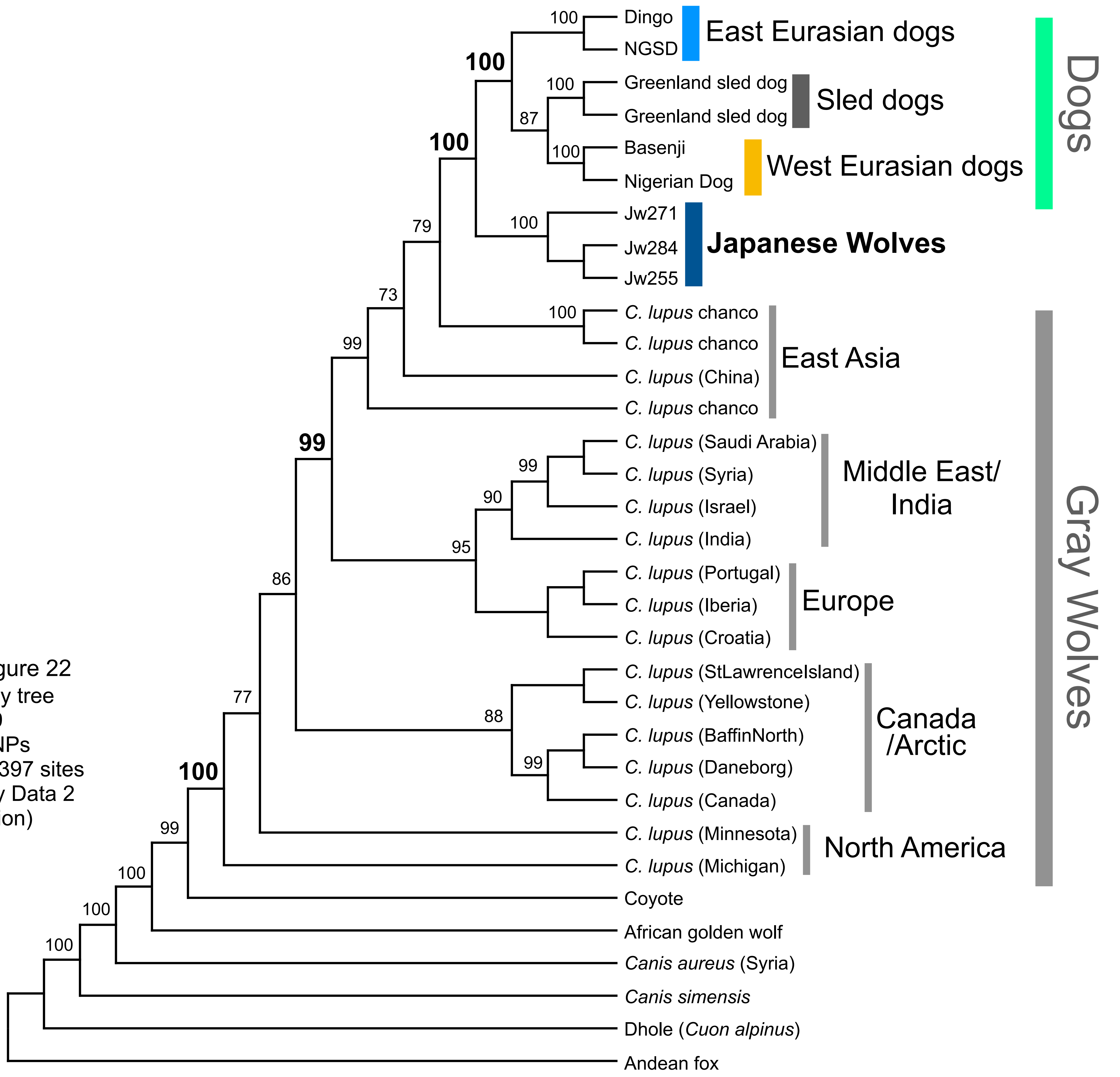

Supplementary Figure 22
Maximum parsimony tree based on on 17,489 unlinked biallelic SNPs extracted from 179,397 sites (see Supplementary Data 2 for sample information)

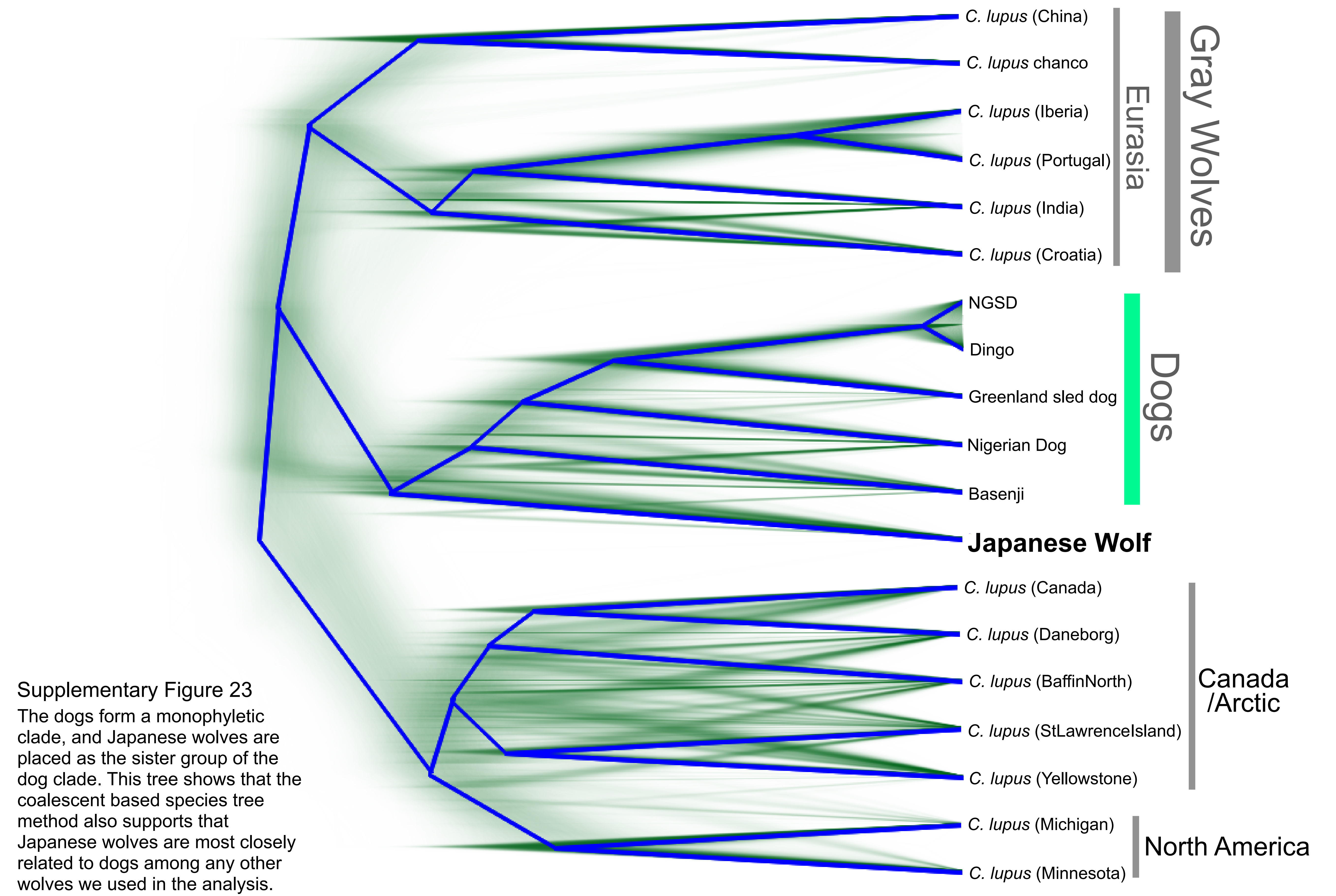

Supplementary Figure 23
The dogs form a monophyletic clade, and Japanese wolves are placed as the sister group of the dog clade. This tree shows that the coalescent based species tree method also supports that Japanese wolves are most closely related to dogs among any other wolves we used in the analysis.

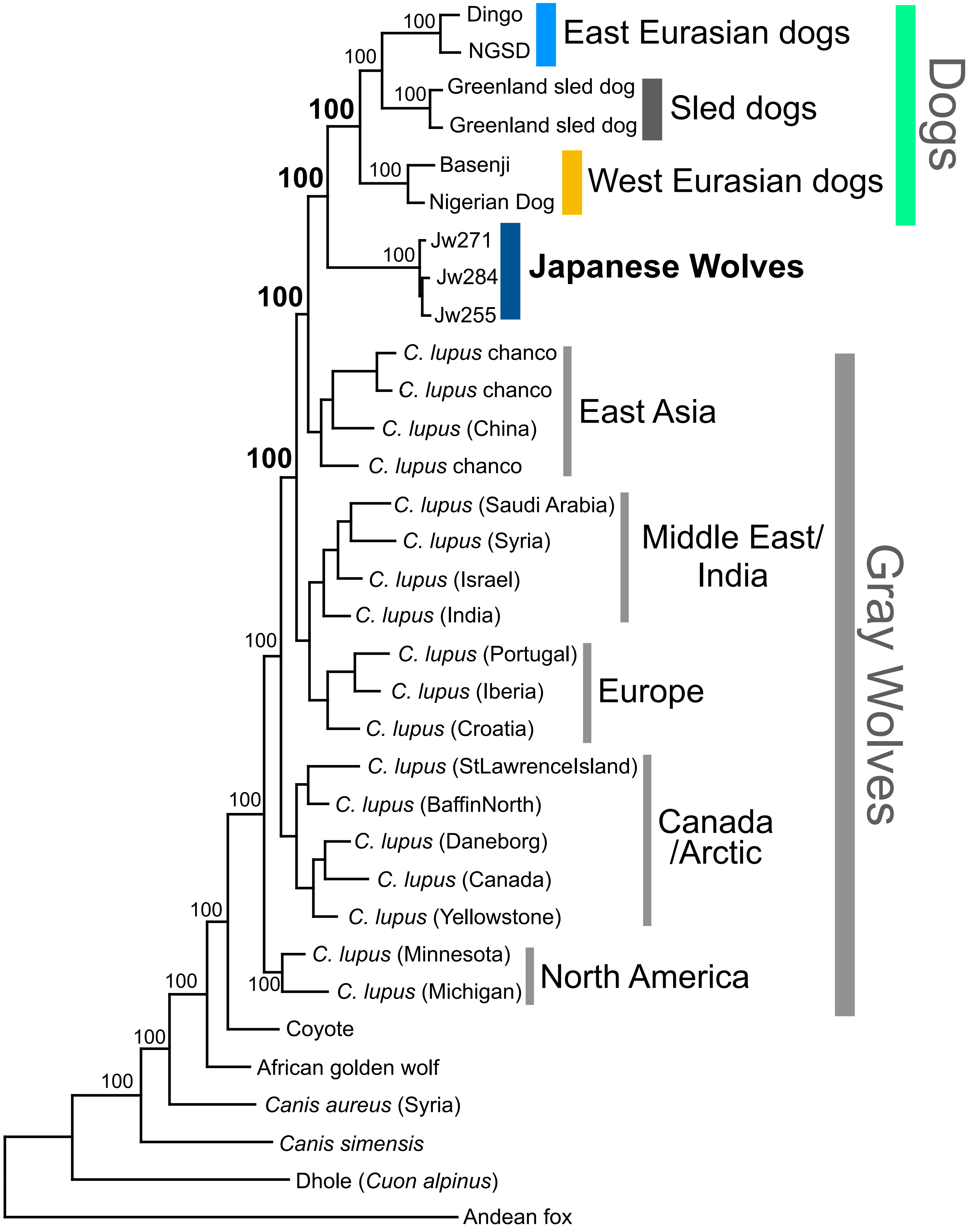

Supplementary Figure 24
Maximum likelihood tree based on on 4,864,601 biallelic SNPs (see Supplementary Data 2 for sample information)

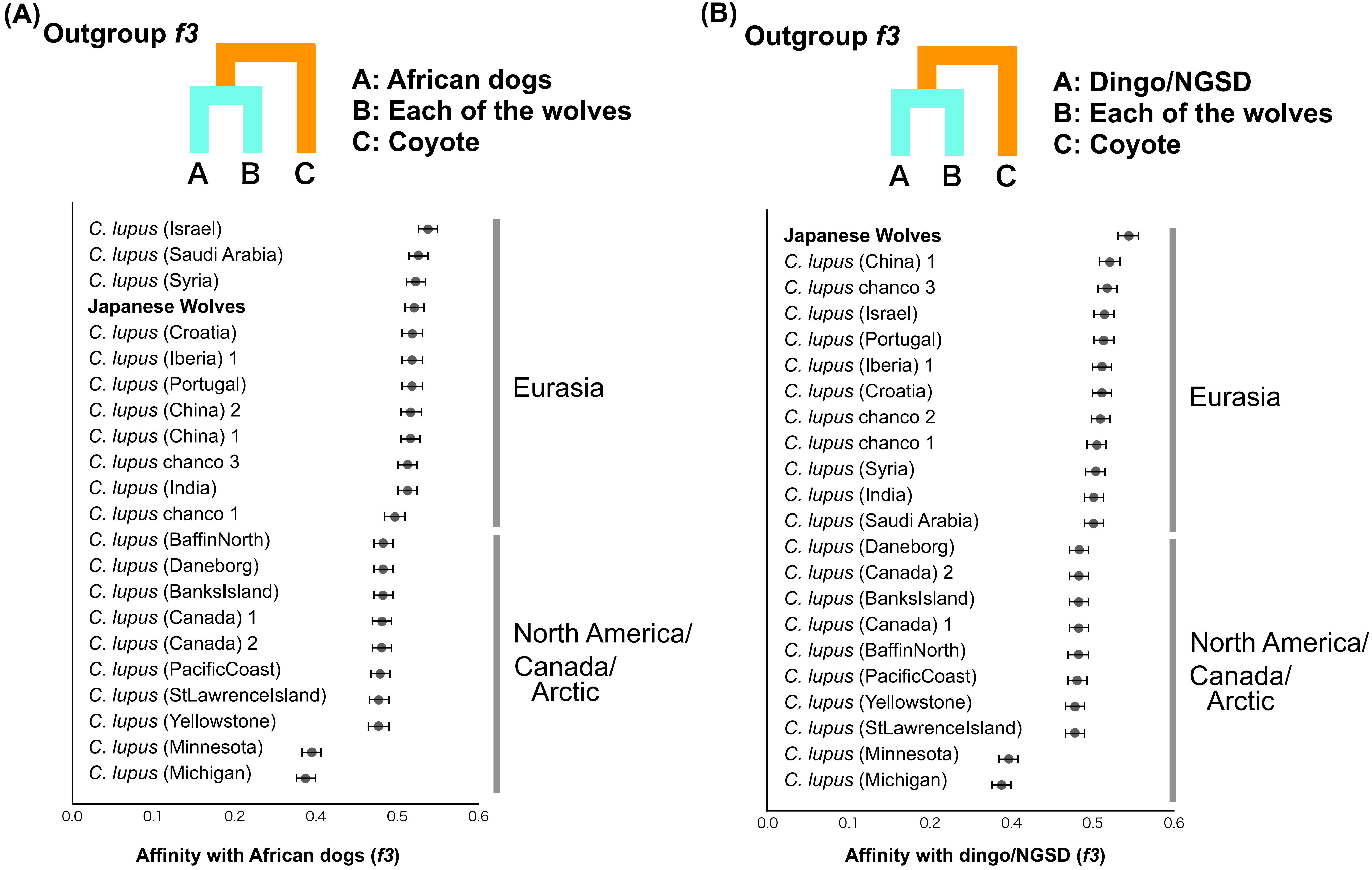

Supplementary Figure 25

Shared genetic drift between African dogs (A) and Dingo/NGSD (B) and gray wolves measured by outgroup *f*3 statistics. Each of the African dogs and Dingo/NGSD individuals were used as populations. Each *f3* statistical value is plotted in order of highest to lowest value from the top, and the names of the wolves are shown on the left side of each panel. Error bars represent standard errors (n = 12 for A, n = 8 for B). Source data are provided as a Source Data file.

Supplementary Figure 26

f4 statistics testing the genetic affinity of the Japanese wolf with all other dogs. All Japanese wolves were used as a population. Z scores for each combination are plotted. We computed *f4* statistics in the schematic representation (A) and (B), and X represented any possible other dogs. Each Z score is listed in order of highest to lowest value from the top. The names of the dogs are shown on the left or right sides of the panels. Source data are provided as a Source Data file (n = 10).

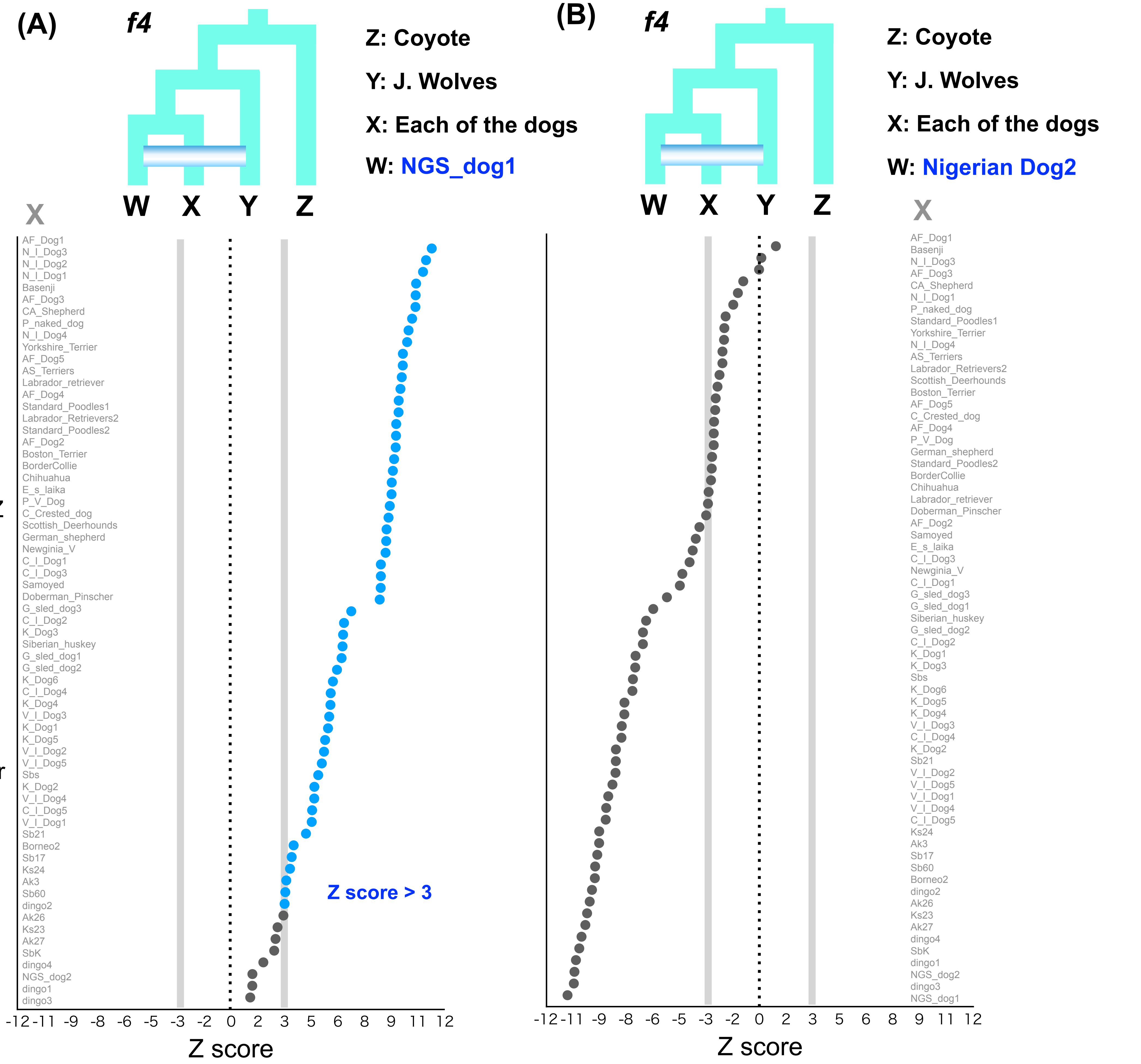

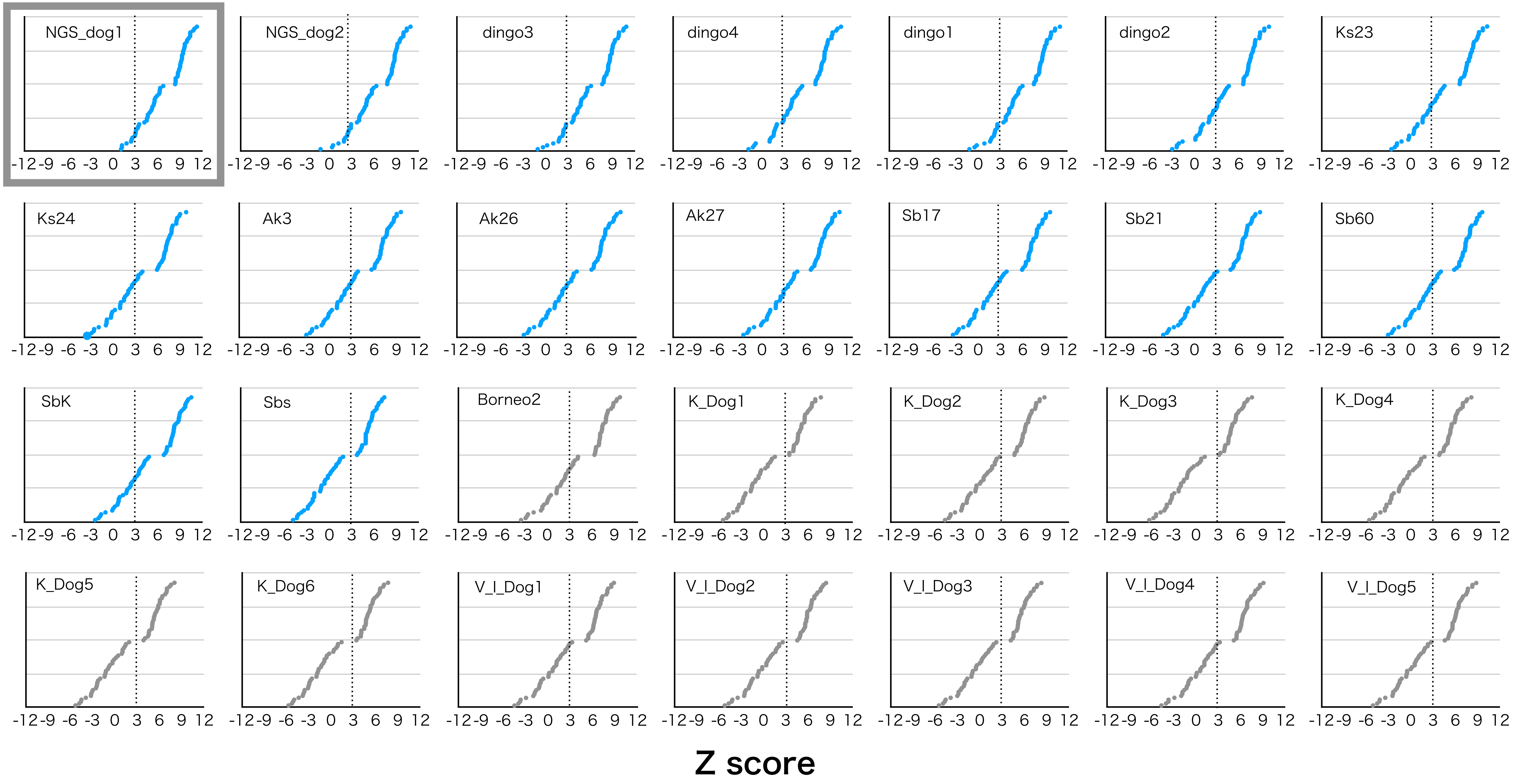

Supplementary Figure 27

f4 statistics testing the genetic affinity of the Japanese wolf with all other dogs. All Japanese wolves were used as a population. Z scores for each combination are plotted. We computed *f4* statistics where W in the schematic representation is shown and fixed in each panel and X represented any possible other dogs. Each Z score is listed in order of highest to lowest value from the top. Dotted line shows the Z score 3. The highest (NGS_dog1, surrounded by gray square) affinity to the Japanese wolf is show in Supplementary Figure 26A. Source data are provided as a Source Data file (n = 10).

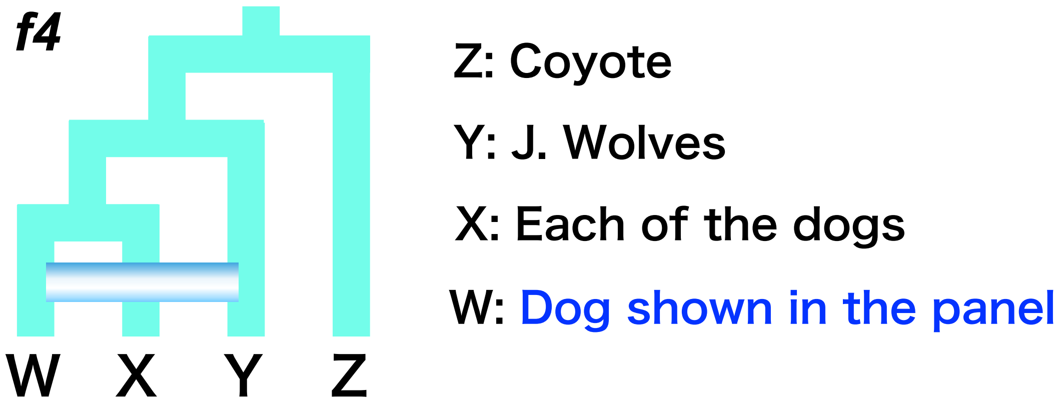

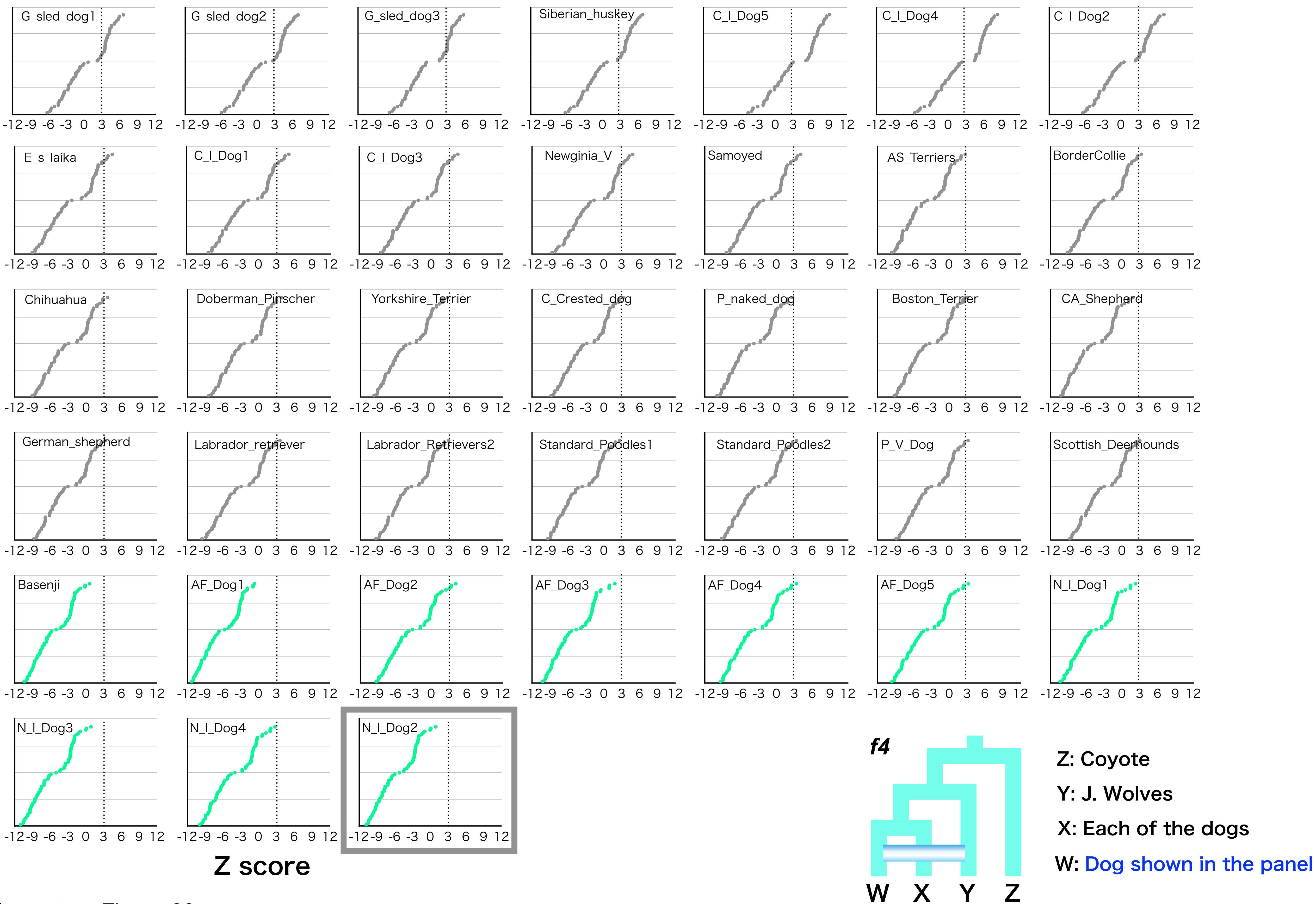

Supplementary Figure 28

f4 statistics testing the genetic affinity of the Japanese wolf with all other dogs. All Japanese wolves were used as a population. Z scores for each combination are plotted. We computed *f4* statistics where W in the schematic representation is shown and fixed in each panel and X represented any possible other dogs. Each Z score is listed in order of highest to lowest value from the top. Dotted line shows the Z score 3. The lowest (Nigerian Dog2, surrounded by gray square) affinity to the Japanese wolf is show in Supplementary Figure 26B. Source data are provided as a Source Data file (n = 10).

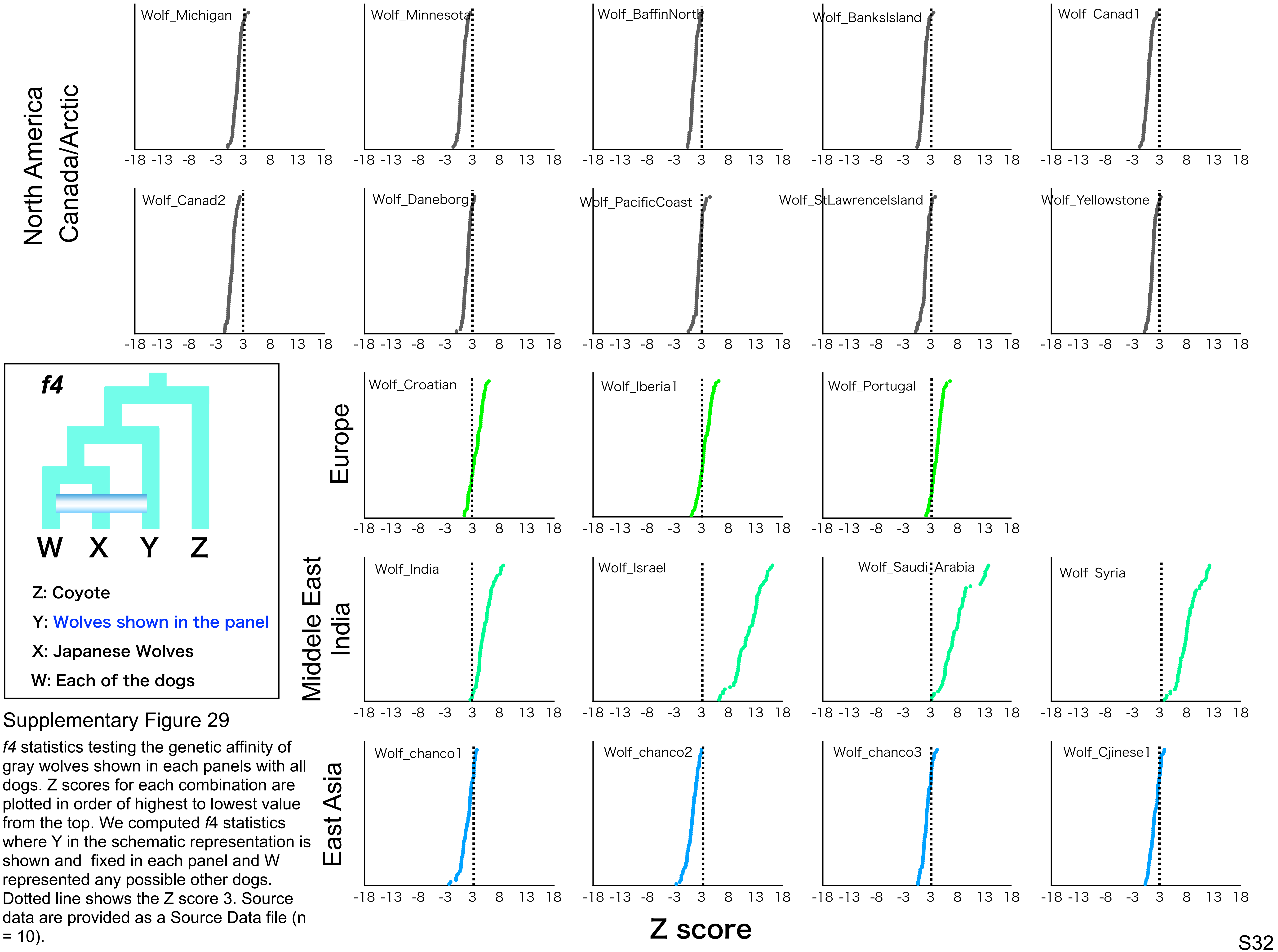

Supplementary Figure 29

f4 statistics testing the genetic affinity of gray wolves shown in each panels with all dogs. Z scores for each combination are plotted in order of highest to lowest value from the top. We computed *f4* statistics where Y in the schematic representation is shown and fixed in each panel and W represented any possible other dogs. Dotted line shows the Z score 3. Source data are provided as a Source Data file (n = 10).

Supplementary Figure 30
Maximum likelihood trees based on 157,906 unlinked biallelic SNPs extracted from 1,916,277 sites. Node labels indicate bootstrap replicates.

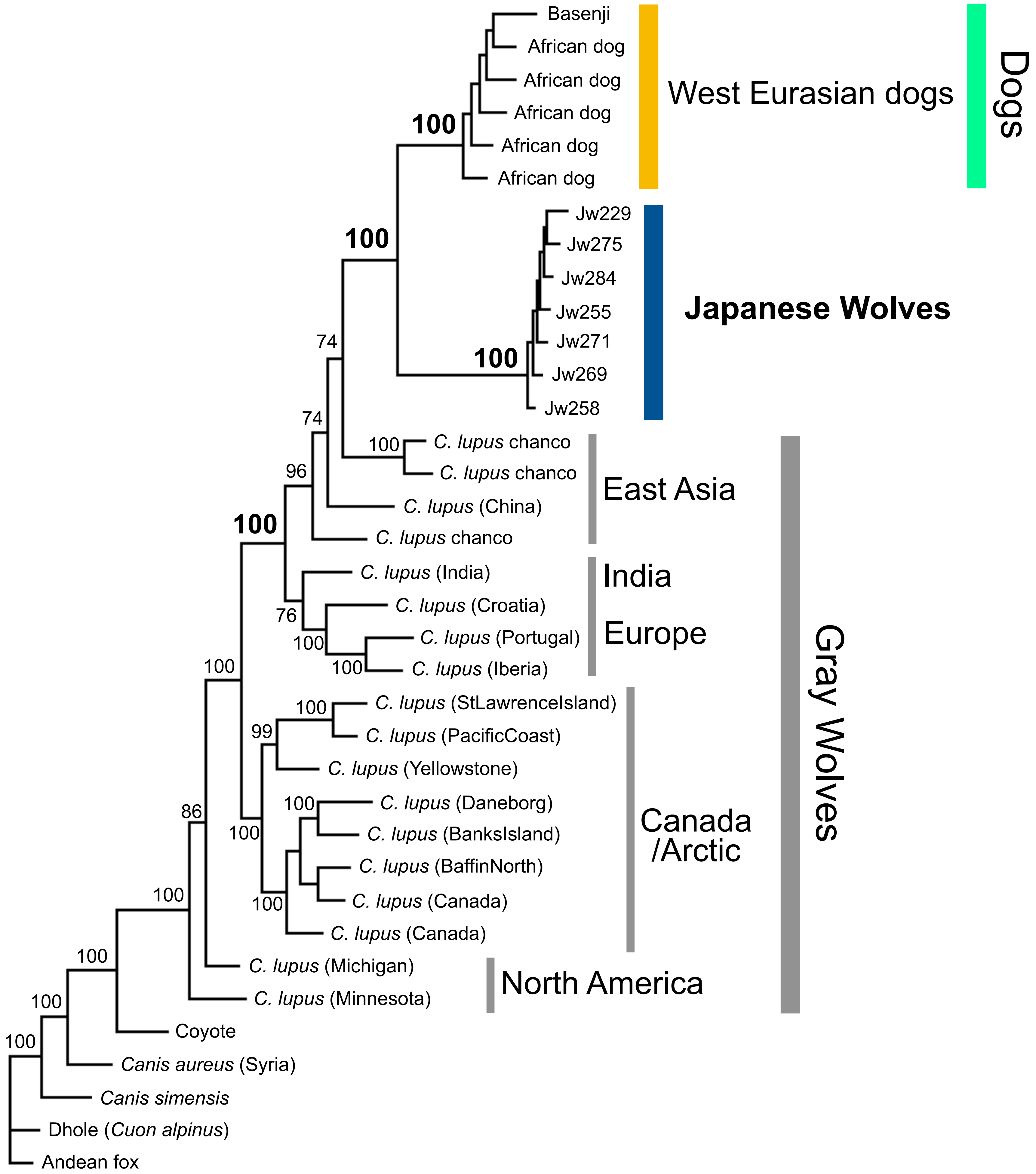

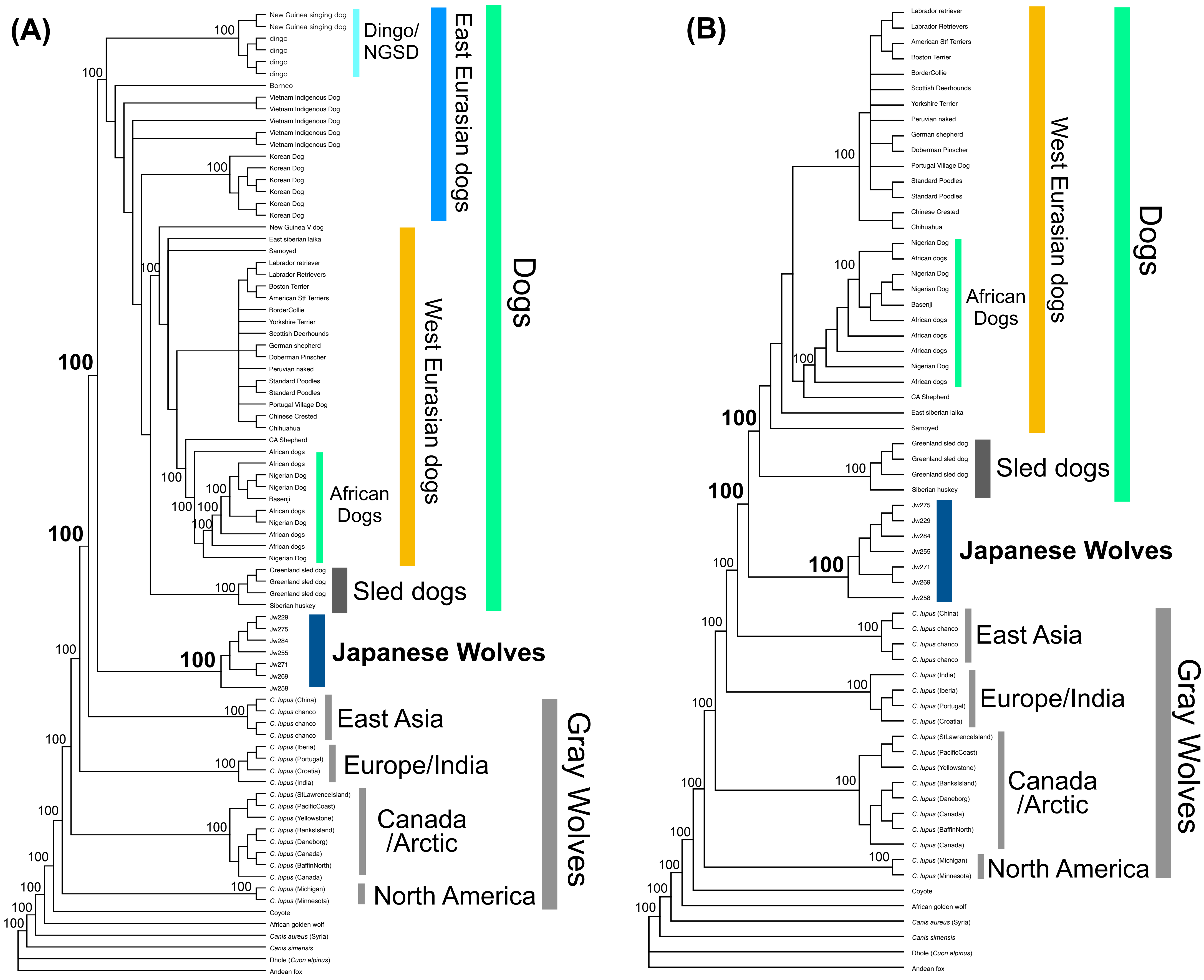

Supplementary Figure 31

Maximum likelihood trees based on (A) 327,402 unlinked biallelic SNPs extracted from 2,065,002 sites (the Japanese dogs were excluded), and (B) 327,402 unlinked biallelic SNPs extracted from 2,065,002 sites (the East Eurasian dogs were excluded). Node labels indicate bootstrap replicates.

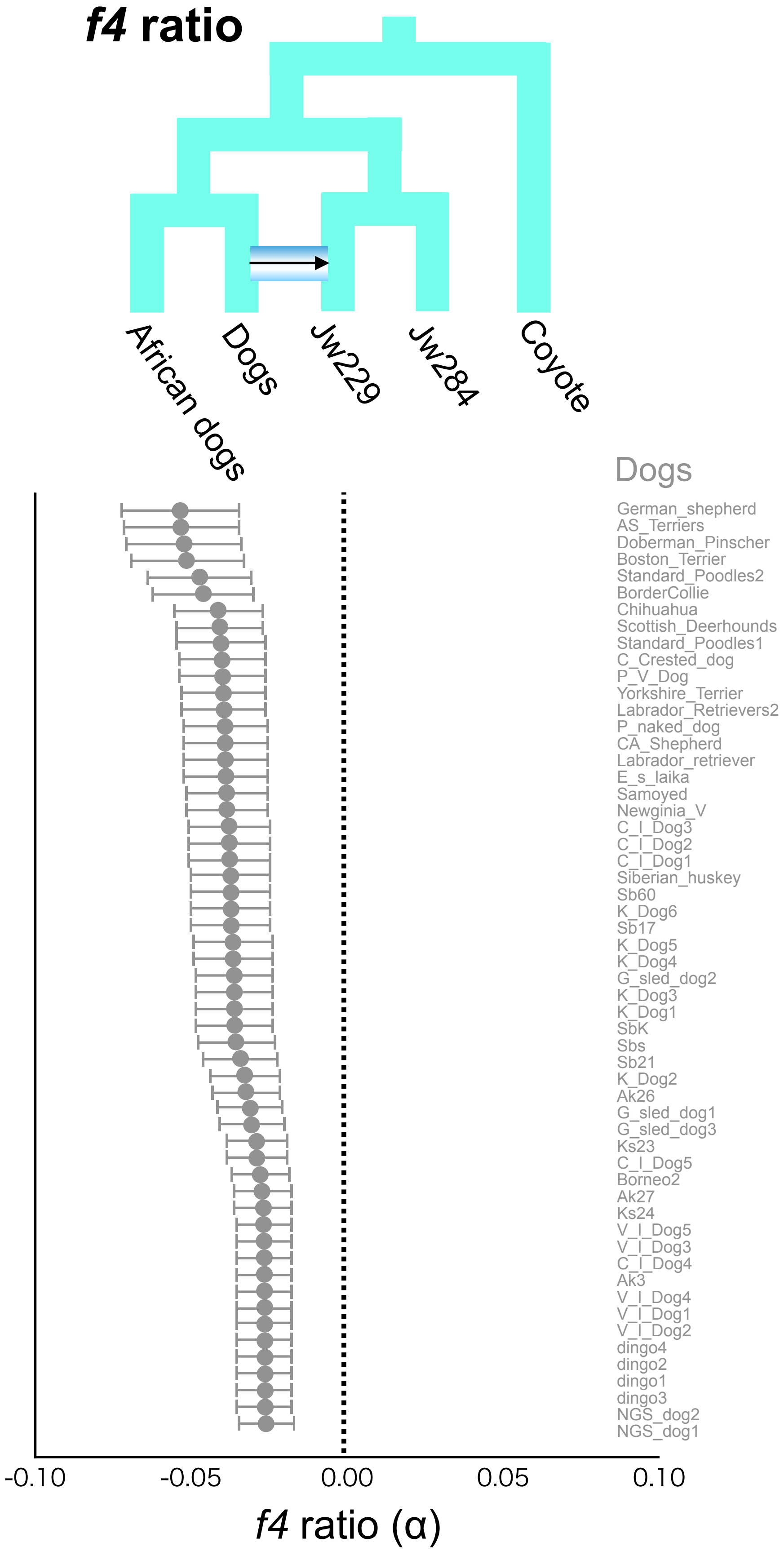

Supplementary Figure 32

f4-ratio test to estimate proportion of genome introgression from dogs to the Japanese wolf. Each *f4*-ratio α value is plotted in order of lowest to highest value from the top, and the names of the dogs are shown on the right side of the panel. Error bars represent standard errors (n = 14). Source data are provided as a Source Data file.

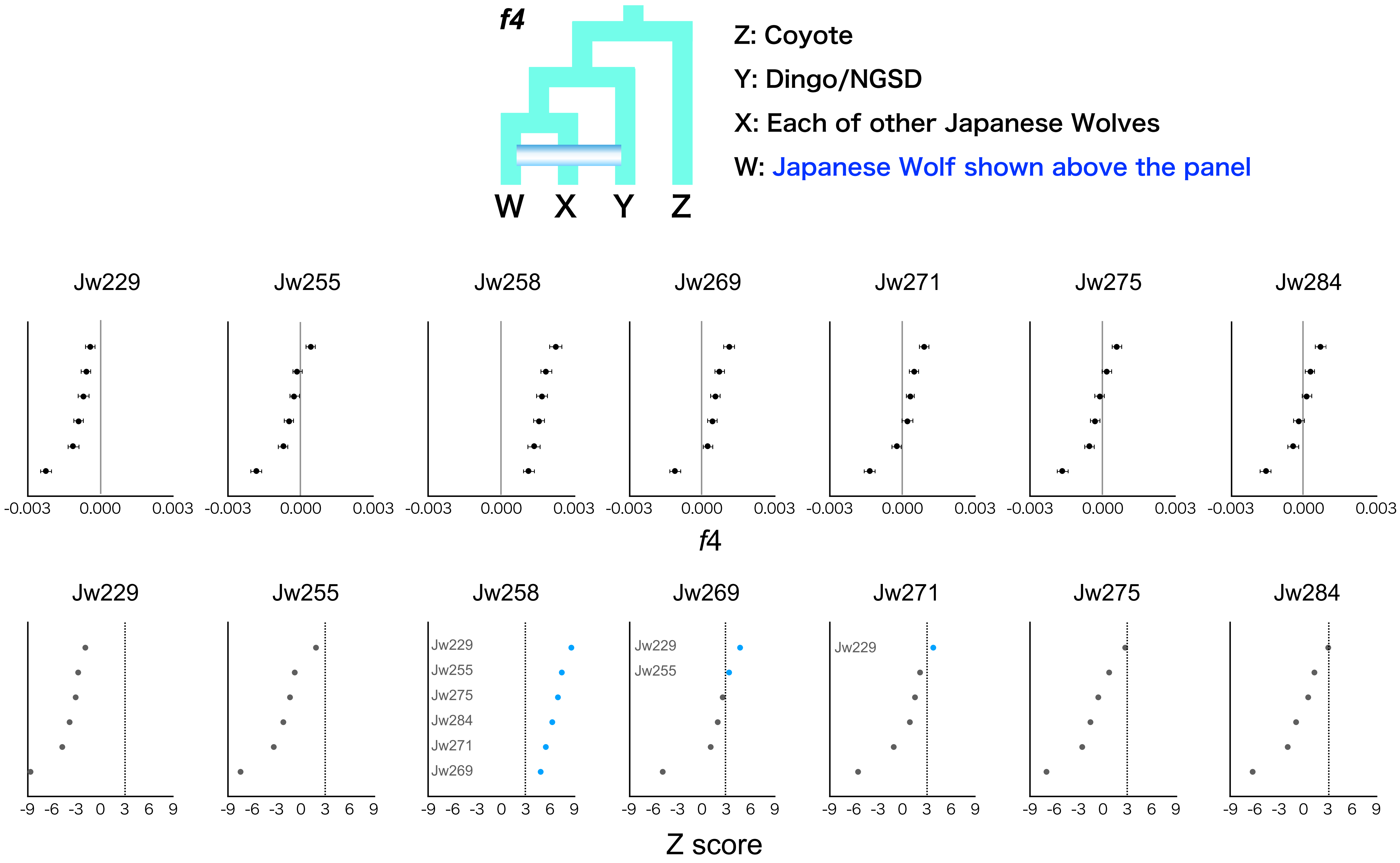

Supplementary Figure 33

(A) *f4* statistics testing the difference of the affinity to dingo/NGSD between the Japanese wolf individuals. *f4* statistics (upper panels) and Z score (lower panels) value is plotted in order of highest to lowest value from the top. Z score above 3 is colored in blue. When the Japanese wolf individual showing a significant affinity to dingo/NGSD, the names of the Japanese wolf individual at the position X in the schematic representation are shown on the left side of Z score panel. Source data are provided as a Source Data file. Error bars represent standard errors (n = 9).

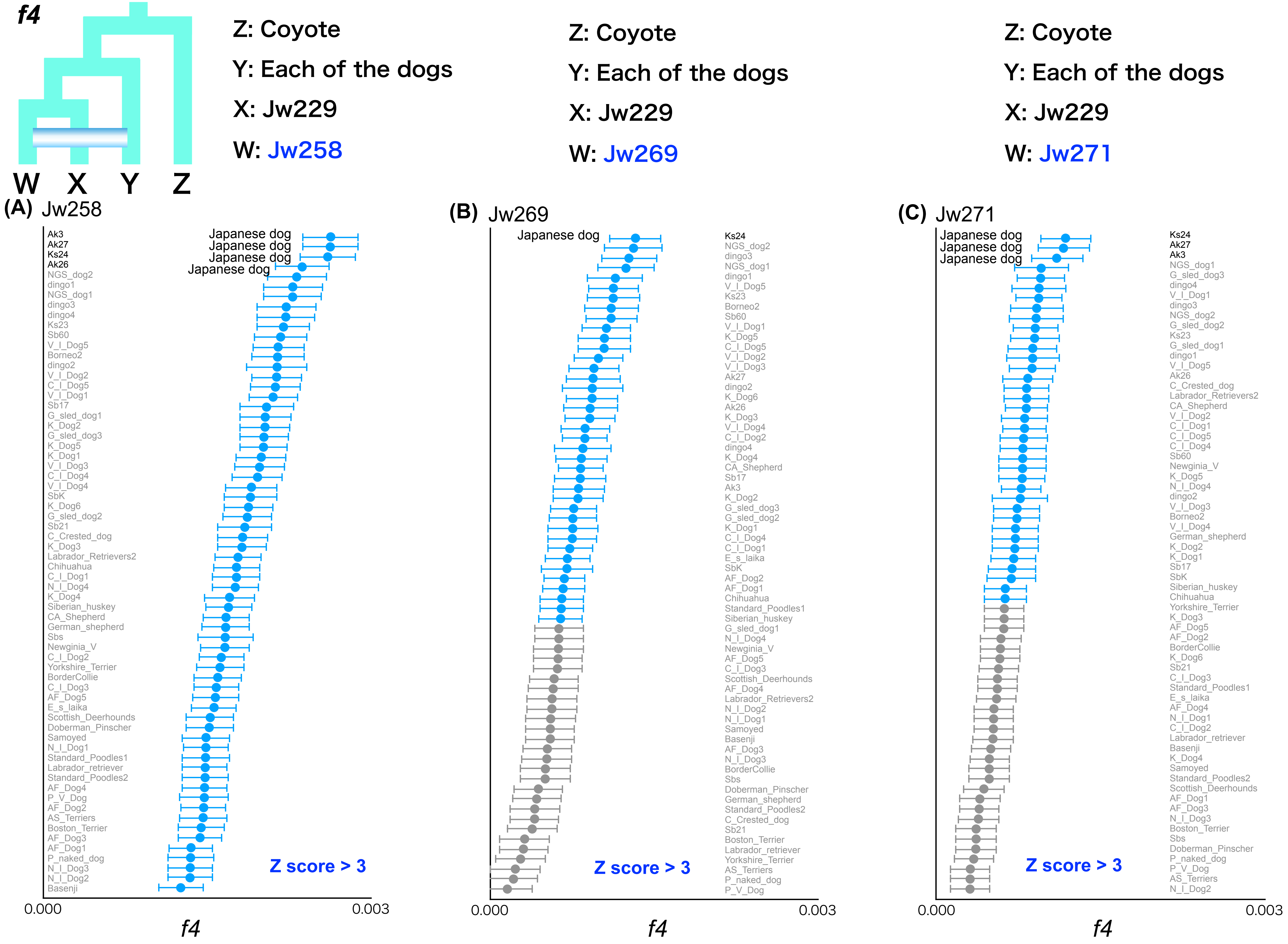

Supplementary Figure 34

(A) *f4* statistics testing the relationships between (A) Jw258, (B) Jw269, and (C) Jw271 and all dog individuals. Each Z score is plotted in order of highest to lowest value from the top, and the names of dogs are shown on theft or right sides of each panel (see Supplementary Data 2). Z score above 3 is colored in blue. Error bars represent standard errors (n = 4). Source data are provided as a Source Data file.

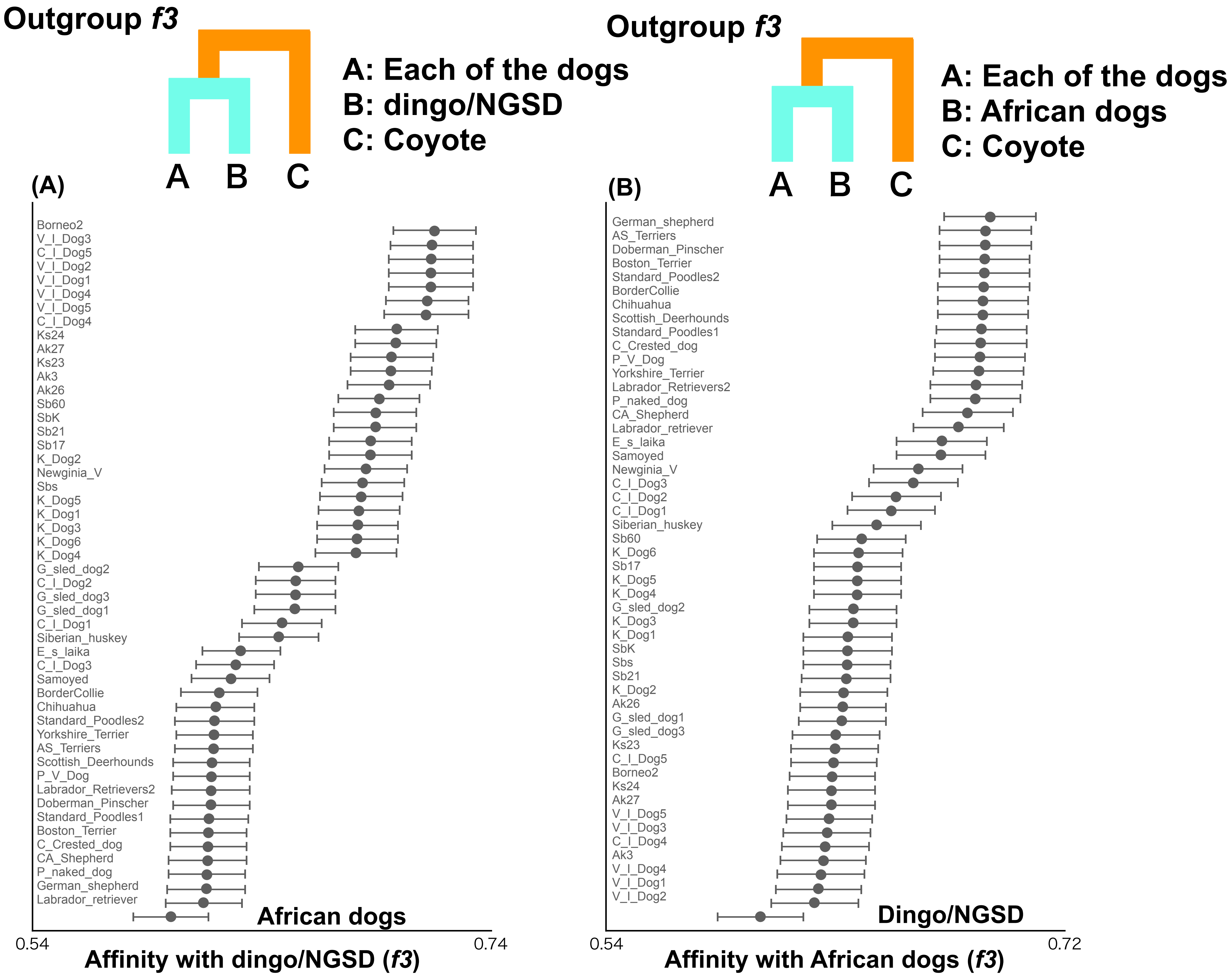

Supplementary Figure 35

Shared genetic drift between (A) dingo/NGSD and (B) African dogs and all dogs measured by outgroup *f*3 statistics. Each of the African dogs and dingo/NGSD individuals were used as populations. Each *f3* value is plotted in order of highest to lowest value from the top, and the names of the dogs are shown on the left side of the panel. Error bars represent standard errors (n = 8 for A, n = 12 for B). Source data are provided as a Source Data file.

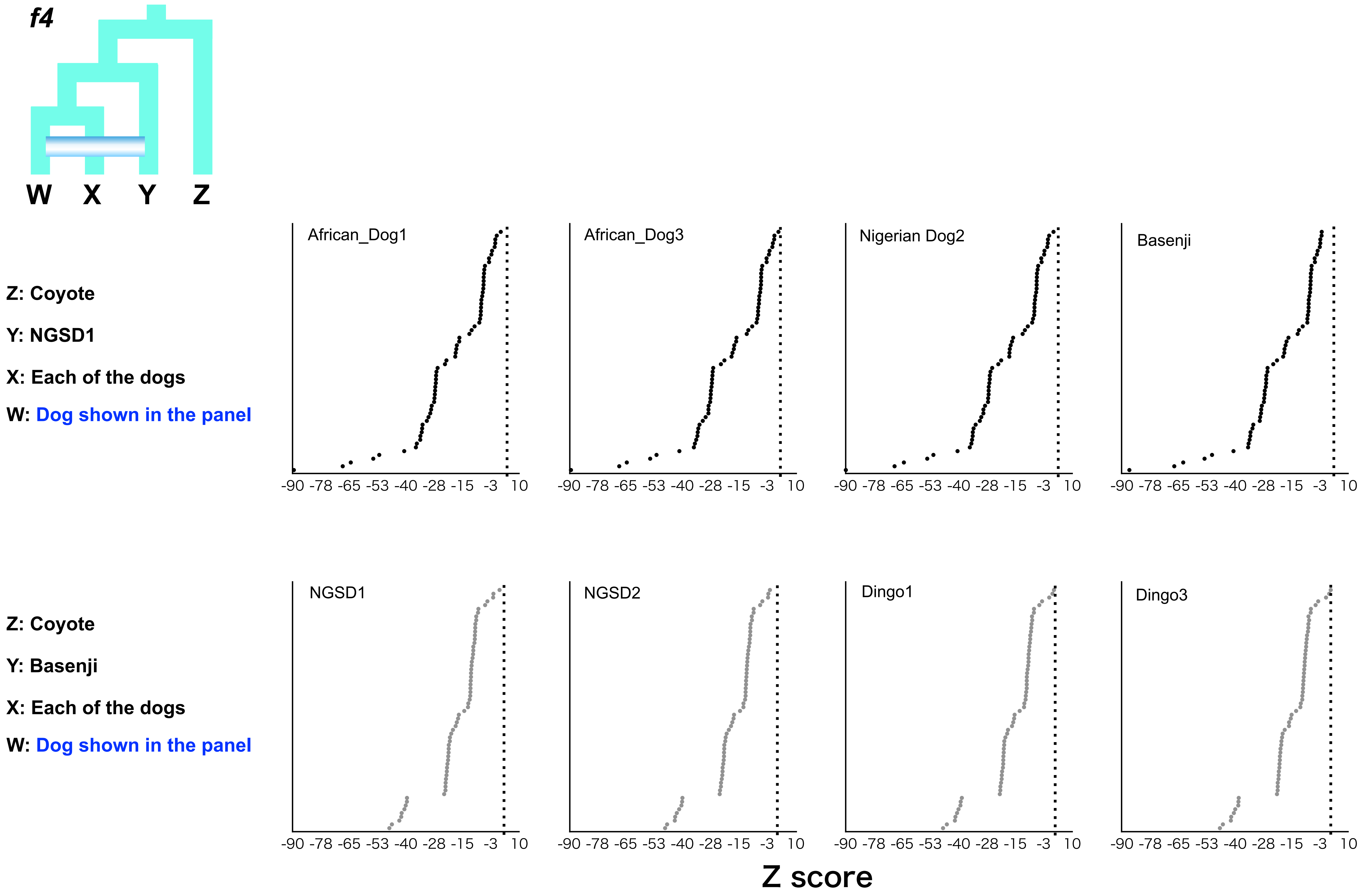

Supplementary Figure 36

f4 statistics testing the affinity of NGSD1 with African dogs (upper panels) and that of Basenji with dingo/NGSD dogs (lower panels). All Z scores were under 3. Source data are provided as a Source Data file (n = 4).

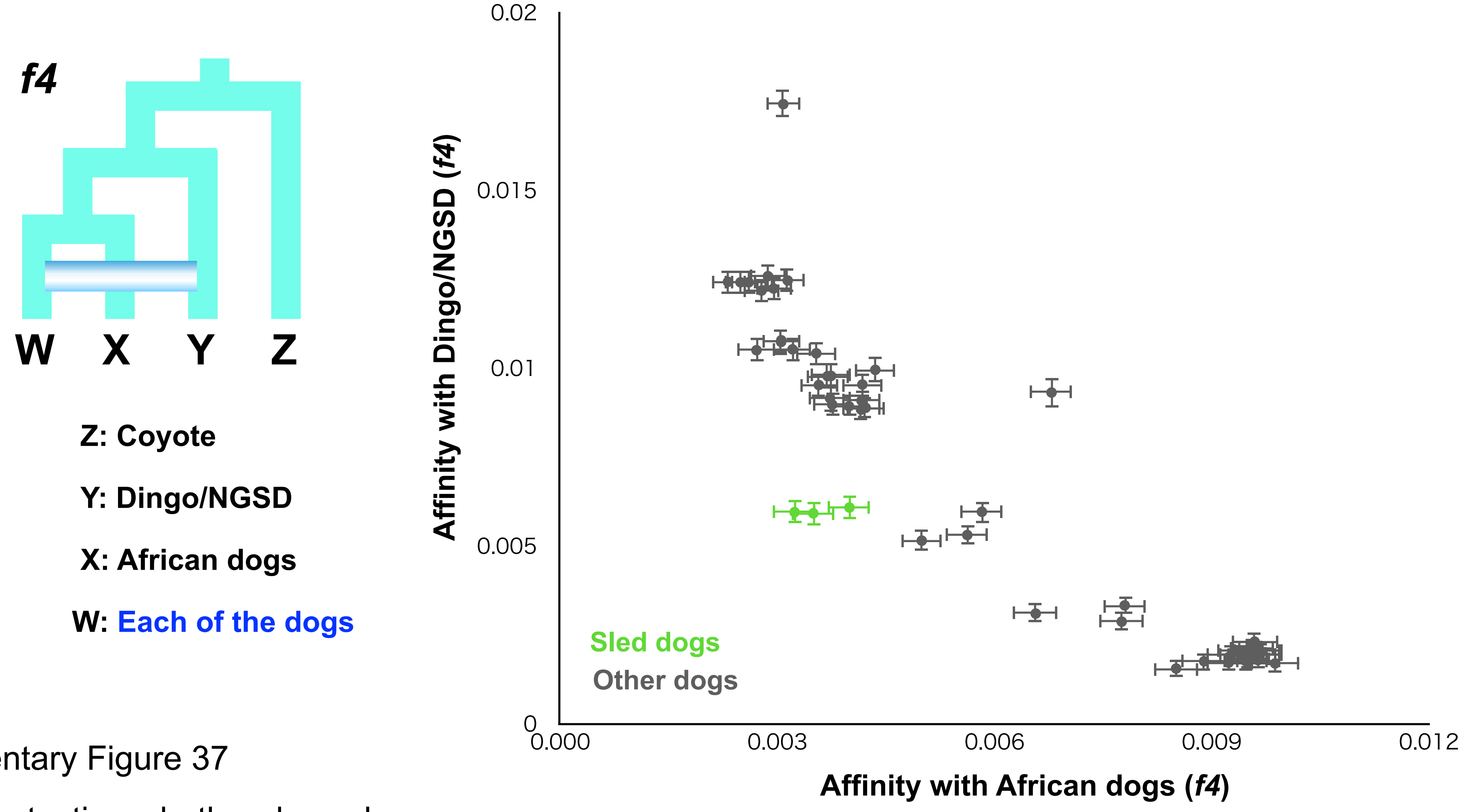

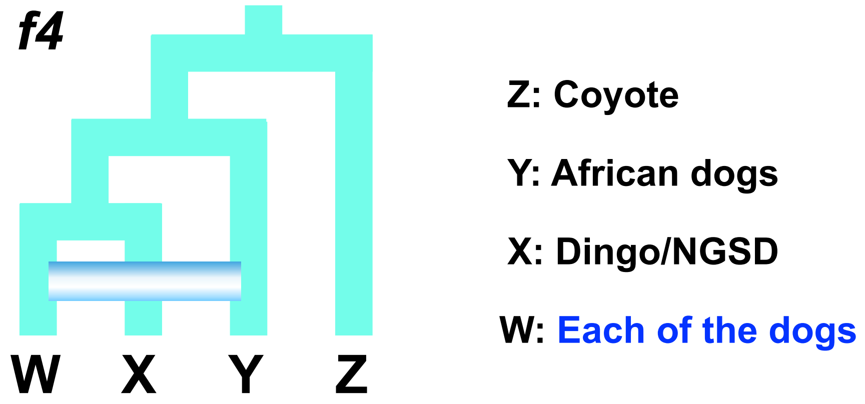

Supplementary Figure 37

f4 statistics testing whether dogs share more alleles with African dogs (x-axis) or dingo/NGSD (y-axis) compared with dingo/NGSD and African dogs, respectively. Dots show the *f4* statistics, and horizontal and vertical error bars represent standard errors for the test with the African dogs (x-axis, n = 18) and dingo/NGSD (y-axis, n = 18), respectively. Each of the African dogs and dingo/NGSD individuals were used as populations. Source data are provided as a Source Data file.

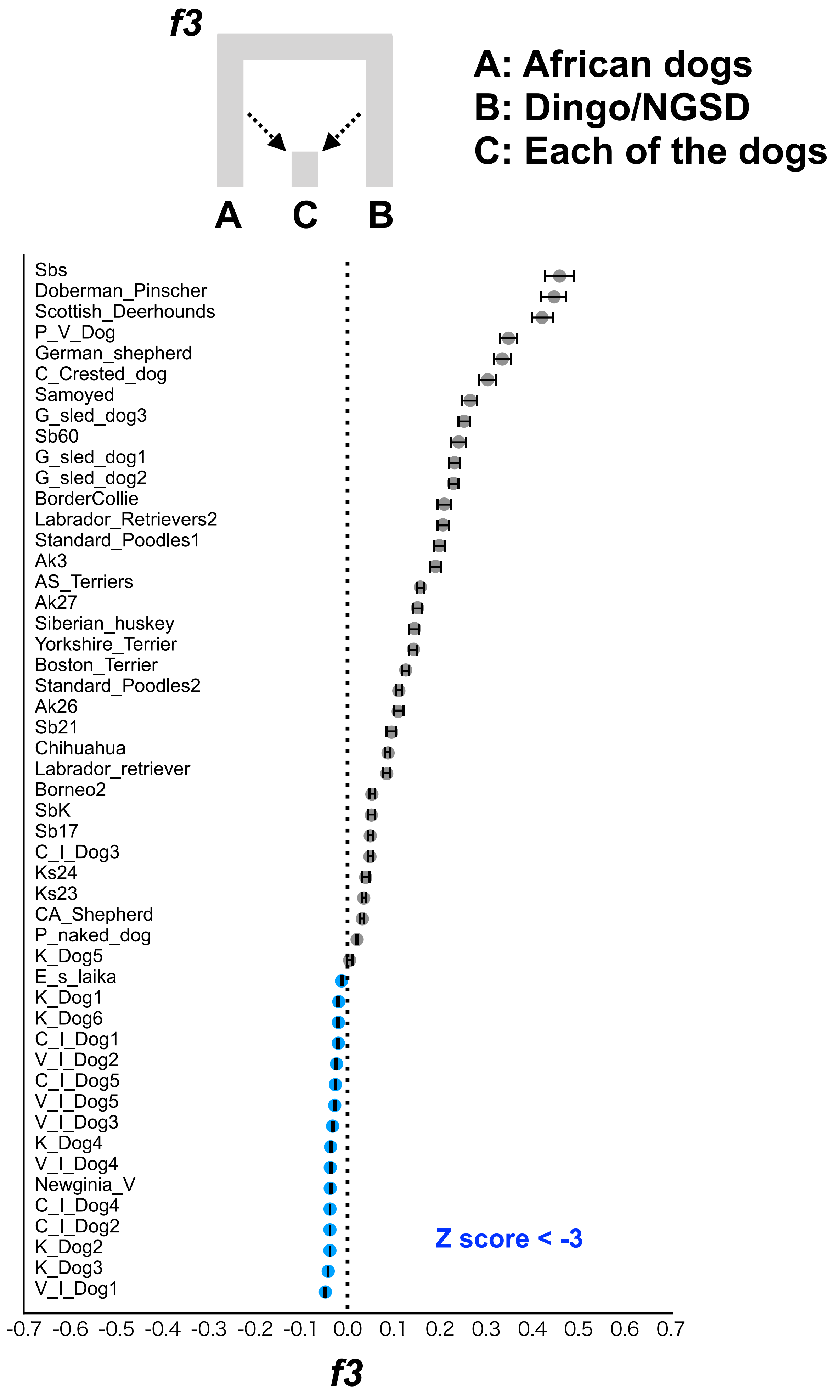

Supplementary Figure 38

f3 statistics testing the genomic mixture of African and Dingo/NGSD dogs in all dogs. Z score under -3 is colored in blue. Each of the African dogs and Dingo/NGSD individuals were used as populations. Each *f3* value is plotted in order of highest to lowest value from the top, and the names of the dogs are shown on the left side of the panel. Error bars represent standard errors (n = 17). Source data are provided as a Source Data file.

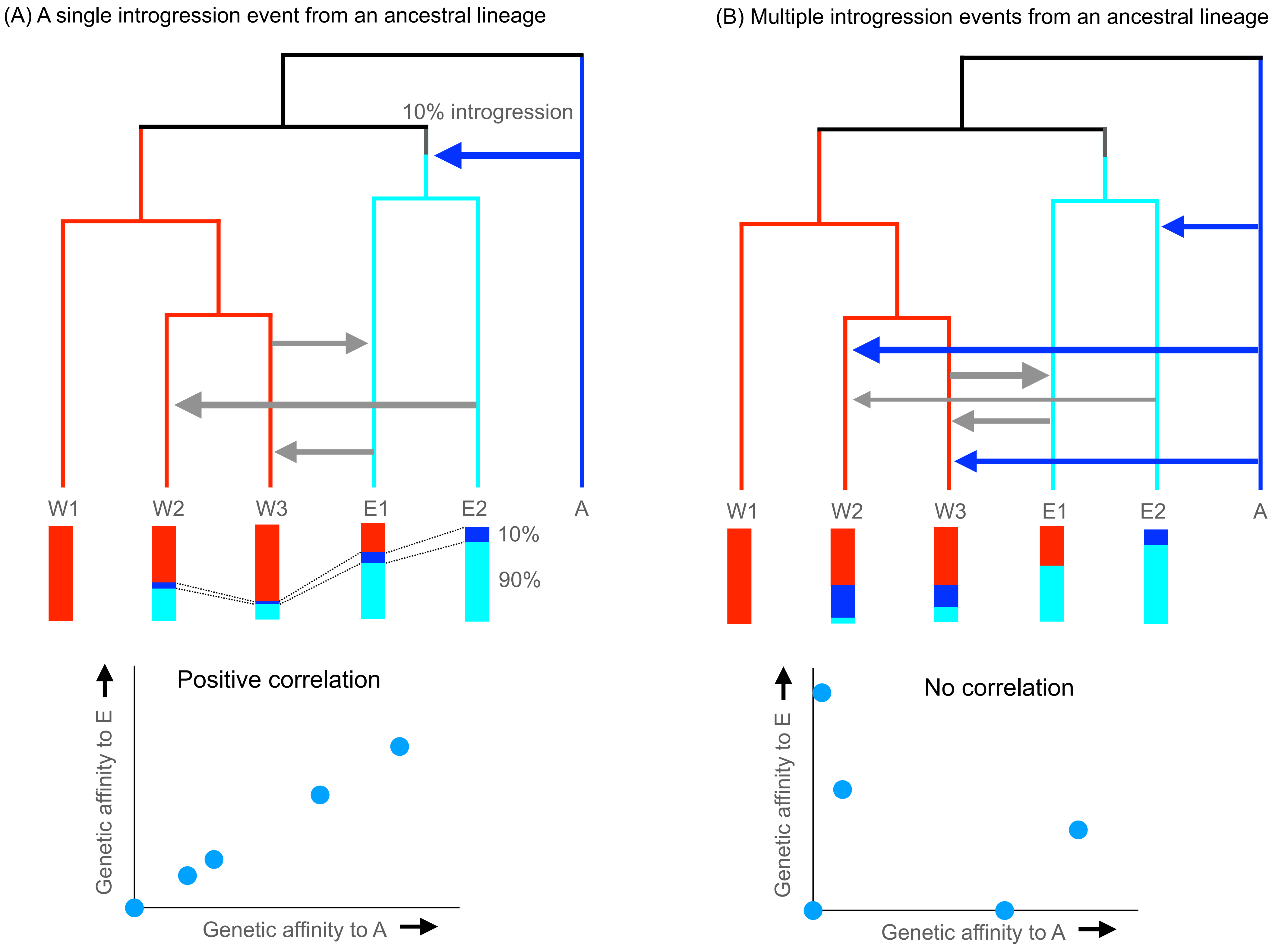

Supplementary Figure 39

Models of a single and multiple introgression events and diffusion of the introgressed genome

If there was a single event of introgression from ancestral lineage A (shown in blue) to lineage E (shown in light blue), for example, 10% of the genome introgressed from lineage A to lineage E (A). After the first introgression, the genome of lineage E contains 10% of the genome of lineage A. When the genome of lineage E introgresses to lineage W, the ratio of the genomes of E to A (E: 90%, A: 10%) is maintained in the genome of lineage W.

Conversely, when the genome of lineage W introgresses to lineage E, the ratio of the genomes of lineage E and A is maintained. Therefore, genetic affinity with A positively correlates with genetic affinity with E in all individuals.

Next, assuming that there were multiple introgression events from ancestral lineage A (blue) to lineage E (shown in light blue) (B). In this case, the ratio of the genomes of lineage E to A is not consistent, and genetic affinity with A show no correlation with genetic affinity with E.

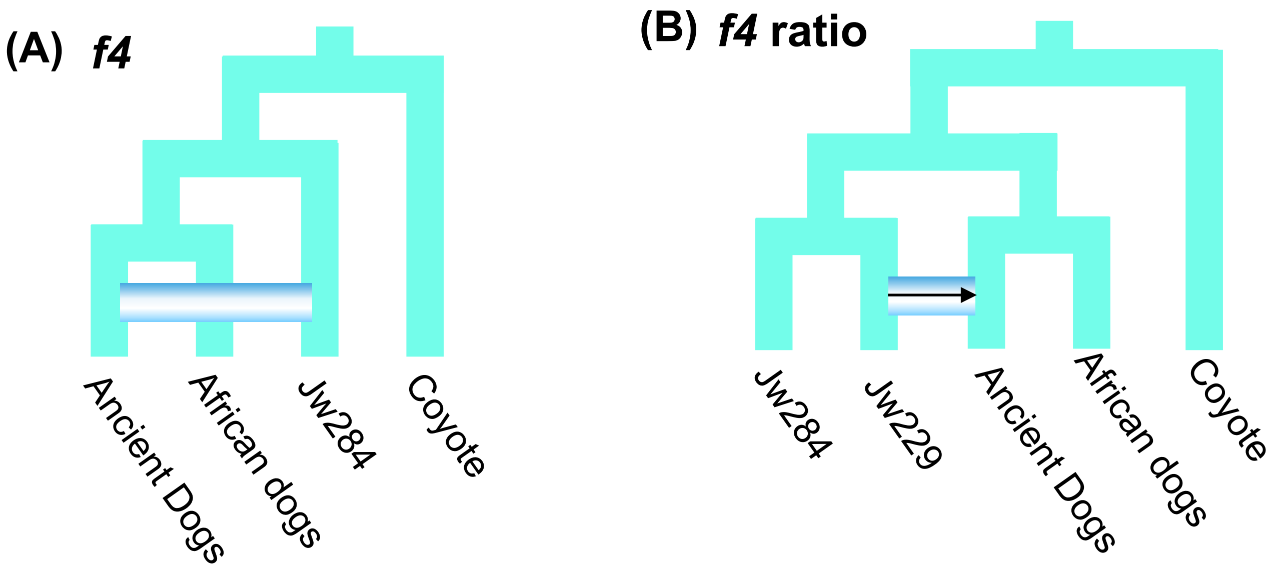

Supplementary Figure 40

The calculation of *f4* and f*4-ratio* in Supplementary Data 4 are based on the schematic representation (A) and (B), respectively.

Supplementary References

Niemann J*, et al.* Extended survival of Pleistocene Siberian wolves into the early 20th century on the island of Honshū. *Iscience* **24**, 101904 (2021).

Segawa T*, et al.* Paleogenomics reveals independent and hybrid origins of two morphologically distinct wolf lineages endemic to Japan. *Curr Biol* **32**, 2494-2504. e2495 (2022).
