# Supplementary material for: Japanese wolves are most closely related to dogs and share DNA with East Eurasian dogs
Source: Nat Commun. 2024 Feb 23;15:1680. doi: 10.1038/s41467-024-46124-y (PMC10891106; doi:10.1038/s41467-024-46124-y)
Supplement: Supplementary file 2 — Description of Additional Supplementary Files [file 41467_2024_46124_MOESM2_ESM.pdf]

Description of Additional Supplementary Files

Supplementary Data 1

Determined sequences in this study

Supplementary Data 2

Sample information

Supplementary Data 3

Genomic regions for a phylogenetic tree based on coalescent

Supplementary Data 4

Gene flow between Japanese Wolf and ancient dogs

Supplementary Data 5

Mapping rate (%) of reads with Japanese Wolf specific substitutions in the mitochondria DNA
