# Supplementary material for: Japanese wolves are most closely related to dogs and share DNA with East Eurasian dogs
Source: Nat Commun. 2024 Feb 23;15:1680. doi: 10.1038/s41467-024-46124-y (PMC10891106; doi:10.1038/s41467-024-46124-y)
Supplement: Supplementary file 8 — Reporting Summary [file 41467_2024_46124_MOESM8_ESM.pdf]

nature portfolio

Corresponding author(s): Naotaka Ishiguro, Yohey Terai

Last updated by author(s): Dec 15, 2023

Reporting Summary

Nature Portfolio wishes to improve the reproducibility of the work that we publish. This form provides structure for consistency and transparency in reporting. For further information on Nature Portfolio policies, see our Editorial Policies and the Editorial Policy Checklist.

Statistics

For all statistical analyses, confirm that the following items are present in the figure legend, table legend, main text, or Methods section.

n/a	Confirmed	
☐	☒	The exact sample size (*n*) for each experimental group/condition, given as a discrete number and unit of measurement
☐	☒	A statement on whether measurements were taken from distinct samples or whether the same sample was measured repeatedly
☐	☒	The statistical test(s) used AND whether they are one- or two-sided<br>*Only common tests should be described solely by name; describe more complex techniques in the Methods section.*
☒	☐	A description of all covariates tested
☐	☒	A description of any assumptions or corrections, such as tests of normality and adjustment for multiple comparisons
☐	☒	A full description of the statistical parameters including central tendency (e.g. means) or other basic estimates (e.g. regression coefficient) AND variation (e.g. standard deviation) or associated estimates of uncertainty (e.g. confidence intervals)
☒	☐	For null hypothesis testing, the test statistic (e.g. *F*, *t*, *r*) with confidence intervals, effect sizes, degrees of freedom and *P* value noted<br>*Give P values as exact values whenever suitable.*
☐	☒	For Bayesian analysis, information on the choice of priors and Markov chain Monte Carlo settings
☒	☐	For hierarchical and complex designs, identification of the appropriate level for tests and full reporting of outcomes
☒	☐	Estimates of effect sizes (e.g. Cohen's *d*, Pearson's *r*), indicating how they were calculated

Our web collection on statistics for biologists contains articles on many of the points above.

Software and code

Policy information about availability of computer code

Data collection	No software was used for data collection.
Data analysis	We used these tools in data analyses: GATK v4.2, vcftools 0.1.16, ADMIXTURE ver. 1.3, ADMIXTOOLS ver. 7.0.1, MEGA ver. X, PhyML ver. 3.2, PAUP* ver. 4a, PLINK ver. 1.9, TreeMix ver. 1.13, BEAST ver. 2.7.4, starbeast2 ver 1.0.0, Densitree ver 2.7.4, Tracer v1.7.2, mapDamage ver. 2.2.0, RStudio version (1.4.1106), Dsuite, and CLC Genomics Workbench ver. 11.

For manuscripts utilizing custom algorithms or software that are central to the research but not yet described in published literature, software must be made available to editors and reviewers. We strongly encourage code deposition in a community repository (e.g. GitHub). See the Nature Portfolio guidelines for submitting code & software for further information.

Data

Policy information about availability of data

All manuscripts must include a data availability statement. This statement should provide the following information, where applicable:

- Accession codes, unique identifiers, or web links for publicly available datasets
- A description of any restrictions on data availability
- For clinical datasets or third party data, please ensure that the statement adheres to our policy

This project has been deposited at the DDBJ (DNA Data Bank of Japan) Sequenced Read Archive under accession numbers SAMD00434011-SAMD00434030. We have used downloaded data from published articles, and all accession numbers are listed in Table S2. We used CanFam 3.1, the reference genome of a dog.

Human research participants

Policy information about studies involving human research participants and Sex and Gender in Research.

Reporting on sex and gender	n/a
Population characteristics	n/a
Recruitment	n/a
Ethics oversight	n/a

Note that full information on the approval of the study protocol must also be provided in the manuscript.

Field-specific reporting

Please select the one below that is the best fit for your research. If you are not sure, read the appropriate sections before making your selection.

☐ Life sciences ☐ Behavioural & social sciences ☒ Ecological, evolutionary & environmental sciences

For a reference copy of the document with all sections, see nature.com/documents/nr-reporting-summary-flat.pdf

Ecological, evolutionary & environmental sciences study design

All studies must disclose on these points even when the disclosure is negative.

Study description	In this study, the genomes of nine Japanese wolves and 11 Japanese dogs were newly determined and analyzed. The analyses showed that 1) the Japanese wolf was a unique subspecies of the gray wolf that is genetically distinct from both extant and ancient gray wolves known to date, 2) the Japanese wolf is most closely related to a monophyletic group of dogs, and 3) Japanese wolf ancestry has introgressed into the ancestor of East Eurasian dogs.
Research sample	Nine Japanese wolves and 11 Japanese dogs
Sampling strategy	Nine Japanese wolves and 11 Japanese dogs were sequenced for this study. Japanese Wolf DNAs were extracted and used in our previous studies. Bone powder (0.1 to 0.3 g) was obtained from the mandible (Jw229, Jw255, Jw258, Jw269) and Cranium (Jw271 and Jw275) specimens by using an electric drill after removal of the outer layers of bone by scraping with a sterile razor blade. Powders were also obtained from ventral nasal concha specimens (Jw284, Leiden b, and Leiden c) with a Multi-beads Shocker. For the eight modern dogs, DNA extracted 26 years ago was used to avoid the effects of admixture between Japanese dogs and other dog breeds. Blood samples for these individuals were provided by the veterinary clinics with the permission of the owners. Blood samples for two individuals of Shiba were provided by the veterinary clinics with the permission of the owners. The saliva of an individuals of Shiba was scrubbed by the owner with cotton swabs.<br>We did not calculate the sample size because the number of available Japanese wolves sample is limited. It is also because the number of high-quality genome sequences for canine samples is limited. We tried to collect as many genomic data as possible.
Data collection	Dr. Ishiguro collected the Japanese wovf and the Japanese dog samples. We determined the sequences of nine Japanese wolves and 11 Japanese dogs. Paired-end (2 × 150 bp) sequencing was performed on the Illumina HiSeq X or NovaSeq 6000 platforms.
Timing and spatial scale	The Edo to Maiji periods Japanese wolf specimens are collected from Naturalis Biodiversity Center, Leiden, the Netherlands (originated from Japan), Naturalis Biodiversity Center, Leiden (originated from Japan), Yamanashi, Iwate, Shimane, Kochi, and Nagano Prefectures, Japan. We sequenced the genome DNA in September 2018 to March 2021.
Data exclusions	No data was excluded from our analyses.
Reproducibility	For the Bayesian analysis, we conduct at least two iterations to ensure that the estimations converge to a similar state.
Randomization	In our study, we have not conducted experiments that require randomization.
Blinding	Investigators were not blinded to the origin of the samples. Because Japanese wolf samples are ancient, we need special care or treatment to ensure any unwanted contamination when extracting DNA or sequencing their genome. For the analysis, we included all the samples together, and we labeled the samples after we got the results. Then, there was no risk that investigators' prior knowledge of sample origin would influence the results.

Did the study involve field work? ☐ Yes ☒ No

Reporting for specific materials, systems and methods

We require information from authors about some types of materials, experimental systems and methods used in many studies. Here, indicate whether each material, system or method listed is relevant to your study. If you are not sure if a list item applies to your research, read the appropriate section before selecting a response.

Materials & experimental systems

n/a	Involved in the study
☒	☐ Antibodies
☒	☐ Eukaryotic cell lines
☐	☒ Palaeontology and archaeology
☐	☒ Animals and other organisms
☒	☐ Clinical data
☒	☐ Dual use research of concern

Methods

n/a	Involved in the study
☒	☐ ChIP-seq
☒	☐ Flow cytometry
☒	☐ MRI-based neuroimaging

Palaeontology and Archaeology

Specimen provenance	Japanese wolf specimen provenances are as follows; Leiden_b (Jentink 1887 b RMNH.MAM.39183): Naturalis Biodiversity Center, Leiden, the Netherlands; Leiden_c (Jentink 1887 c RMNH.MAM.39181): Naturalis Biodiversity Center, Leiden, the Netherlands; Jw255: Yamanashi Prefectural Museum; Jw271: Iwate Prefectural Museum; Jw284 (ZMB_Mam_048817): Museum für Naturkunde, Berlin, Germany; Jw275: Kurashiki Museum of Natural History; Jw229: Kataoka Personal Collection, Kochi; Jw258: Ueda High School, Nagano; Jw269: Personal collection.
Specimen deposition	Permission is required to use all specimens.
Dating methods	No new dates are provided.

☒ Tick this box to confirm that the raw and calibrated dates are available in the paper or in Supplementary Information.

Ethics oversight	No ethical approval was required for DNA extraction of Japanese wolf specimens. Only permission of the Museum or the owner was required.

Note that full information on the approval of the study protocol must also be provided in the manuscript.

Animals and other research organisms

Policy information about studies involving animals; ARRIVE guidelines recommended for reporting animal research, and Sex and Gender in Research

Laboratory animals	This study did not involve laboratory animals.
Wild animals	This study did not involve wild animals.
Reporting on sex	This study did not report sex of animals.
Field-collected samples	This study did not involve field-collected samples.
Ethics oversight	The Institutional Animal Care and Use Committee of Anicom Specialty Medical Institute approved the animal protocols and procedures (No. 2020-02).

Note that full information on the approval of the study protocol must also be provided in the manuscript.
